# Brain temperature and its role in physiology and pathophysiology: Lessons from 20 years of thermorecording

**DOI:** 10.1080/23328940.2019.1691896

**Published:** 2019-12-03

**Authors:** Eugene A. Kiyatkin

**Affiliations:** Behavioral Neuroscience Branch, National Institute on Drug Abuse – Intramural Research Program, National Institutes of Health, Baltimore, MD, USA

**Keywords:** Brain metabolism, neural activation, vascular tone, motivated behavior, reward, cocaine, heroin, rats

## Abstract

It is well known that temperature affects the dynamics of all physicochemical processes governing neural activity. It is also known that the brain has high levels of metabolic activity, and all energy used for brain metabolism is finally transformed into heat. However, the issue of brain temperature as a factor reflecting neural activity and affecting various neural functions remains in the shadow and is usually ignored by most physiologists and neuroscientists. Data presented in this review demonstrate that brain temperature is not stable, showing relatively large fluctuations (2-4°C) within the normal physiological and behavioral continuum. I consider the mechanisms underlying these fluctuations and discuss brain thermorecording as an important tool to assess basic changes in neural activity associated with different natural (sexual, drinking, eating) and drug-induced motivated behaviors. I also consider how naturally occurring changes in brain temperature affect neural activity, various homeostatic parameters, and the structural integrity of brain cells as well as the results of neurochemical evaluations conducted in awake animals. While physiological hyperthermia appears to be adaptive, enhancing the efficiency of neural functions, under specific environmental conditions and following exposure to certain psychoactive drugs, brain temperature could exceed its upper limits, resulting in multiple brain abnormalities and life-threatening health complications.

## Introduction

Temperature is traditionally viewed as one of the basic homeostatic parameters of living organisms and its regulation is an essential topic of physiology, which considers the mechanisms determining relative stability of core body temperature following highly variable changes in environmental temperatures. Aristotle (384–322 BC) was probably the first to define temperature as one of five basic biological processes and provided a logical account to what we now call temperature regulation. Although it is known that temperature affects neural activity and neural functions, we have limited knowledge of normal and pathological fluctuations of brain temperature and the mechanisms underlying brain thermal homeostasis and its alterations. These issues are usually ignored by thermophysiologists, who examine neural mechanisms underlying *stability* of body temperature and the development of fever, a stable increase in body temperature usually associated with viral and bacterial infections [1–4]. While not clearly stated in most conceptualizations describing body temperature regulation, brain temperature is usually viewed either as a stable, tightly regulated homeostatic parameter or a parameter that passively follows body temperature changes.

It is well known that the brain plays a crucial role in the regulation of body temperature by detecting changes in environmental temperatures, integrating sensory information, and adjusting, via effector mechanisms, heat production and/or heat loss to the external environment [4,5]. However, the brain itself has high levels of metabolic activity, generating a significant amount of heat. Being only a fraction of the human body’s mass, the brain accounts for ~20% of the organism’s total oxygen consumption in resting conditions [6]. Most energy used for neuronal metabolism is expended during restoration of membrane potentials after electrical discharges [7–10], but significant energy is also used for neural processes not directly related to neuronal electrical activity, particularly for the synthesis of macromolecules as well as for the functioning of glial, endothelial and epithelial cells, which greatly outnumber the amount of neurons. Since all energy used for brain metabolism is finally transformed into heat [6], intense heat production appears to be an essential feature of brain metabolic activity. The brain tissue is also exceptionally well vascularized, receiving 15–20% of total cardiac output [11]. Therefore, intense cerebral blood flow is not only the means to deliver nutrients and oxygen essential for brain metabolic activity but also the means to remove from brain tissue unnecessary products of metabolic degradation, including heat continuously generated through brain metabolism.

The primary goal of this work is to consider brain temperature as an important physiological parameter, which fluctuates within relatively large limits (3–4°C) within the normal physiological continuum at stable ambient temperatures. I will begin with a short historical overview of brain thermorecording, which was used by early physiologists as a tool to study and understand brain functions. Then, I will present data on changes in brain temperature induced by natural arousing stimuli and discuss the mechanisms underlying these physiological fluctuations, particularly the contribution of intra-brain heat production due to metabolic neural activation and heat loss from brain tissue due to changes in cerebral and peripheral vascular tone. I will provide evidence that physiological brain temperature increases (brain hyperthermia) are triggered by changes in neuronal activity and differ from fever, a tonic temperature increase occurring during viral and bacterial infections [1,12]. Then, I will consider changes in brain temperature occurring during three types of natural motivated behavior (sexual, feeding and drinking) and discuss what we can learn from these data on basic mechanisms underlying goal-directed behavior.

After establishing the limits of physiological and pathological brain hyperthermia, I will discuss brain temperature as a factor affecting different measures of neuronal activity, permeability of the blood-brain barrier (BBB), activity of glial cells, brain ionic and water balance, and the structural integrity of different types of brain cells. Here, I will also discuss the role of naturally occurring brain temperature fluctuations as a factor significantly affecting microdialysis and electrochemical evaluations of changes in brain neurochemicals in awake, freely moving animals.

In contrast to physiological fluctuations that are triggered by neuronal activation, brain temperature also can “passively” increase during excessive bodily heat production and/or insufficiency of heat dissipation. These brain temperature increases may exceed upper physiological limits, resulting in pathological brain hyperthermia that significantly alters neural functions and may induce structural damage to brain cells and lethality.

In the final chapters of this review, I will consider brain temperature changes induced by neuroactive drugs, focusing on three popular drugs of abuse (e.g. cocaine, heroin, and 3,4-methylenedioxymethamphetamine or MDMA). In contrast to physiological increases in brain temperature, which are triggered by either direct activation of somatosensory pathways or by simple sensory stimuli that gained salience due to their previous association with positive or negative reinforcers, neuroactive drugs induce their temperature effects through their direct actions on specific central and peripheral neural substrates as well as cerebral and peripheral blood vessels, thus affecting brain metabolic activity and heat dissipation to the external environment. In contrast to relatively modest, transient and uniform physiological brain temperature fluctuations, the effects of drugs are distinct, dose-dependent, and are strongly modulated by activity state and environmental conditions. Furthermore, these effects can be quite different depending on whether the drug is received passively or is self-administered as a part of motivated drug-seeking behavior. This issue will be considered with respect to cocaine and heroin self-administration, two “unnatural” motivated behaviors, which are associated with robust and distinct changes in brain temperature. Despite different temperature responses induced by passively administered heroin and cocaine, the pattern of brain temperature fluctuations occurring during self-administration of these drugs shares many common features and similarities with other types of natural motivated behaviors.

Robust hyperthermia is a known life-threatening complication of overdose of psychomotor stimulants such as MDMA and methamphetamine (METH). I will demonstrate that the brain temperature effects of these drugs are strongly modulated by high activity states and adverse environmental conditions that limit heat dissipation, thus leading to pathological brain hyperthermia that induce multiple functional and structural brain changes that could lead to lethality.

This review paper is primarily focused on our own studies conducted during the last 20 years and presented in multiple experimental reports. While my goal is to provide an overview and discuss the results of multiple studies and stress important discoveries related to brain temperature, this work differs from traditional comprehensive reviews, which present a massive amount of data and can often be boring. In contrast, this work is assembled as a personal journey in the very exciting area of brain thermorecording, which has exciting history and important implications for quite different aspects of physiology and medicine. This journey never had a clearly defined goal and it was not a straight and linear one, having multiple appendices, realizations of our own mistakes, and the appearances of new goals and directions. The best reward for the author will be attention to his work and substantive discussion and critiques, which could result in new ideas, new experiments to test these ideas, and an overall better understanding of the brain and its functions.

## Brain thermorecordining: Historical backgrounds

The middle of the XIX century was evidently the birth time of brain physiology as an experimental science. Several important discoveries in physics and chemistry made at that time catalyzed an intense search for objective parameters to characterize neural and psychic functions. Some of these paths failed but others resulted in groundbreaking discoveries that helped build the foundation of modern neuroscience.

After the discovery of the first law of thermodynamics by Hermann von Helmholtz [13] and wide popularization of thermodynamic ideas within the scientific community, several groups of scientists in France, Germany, Italy, and the United States became focused on brain temperature as an objective parameter that could characterize brain functions and mental activity. They believed that the brain, somewhat like a muscle, manifests its dynamic power by a local increase in heat production, which could be detected by laboratory instruments. Pierre Paul Broca, French physician and anatomist, was obviously the first to explore this approach by using miniature surface thermometers strapped onto the scalps of human subjects [14]. While he was able to detect certain changes in temperature associated with intellectual and emotional activity, these changes were minor and the question remained whether these changes actually reflected true brain temperature or skin temperature affected by skin blood flow. In an attempt to eliminate the influences of blood vessels, Josiah Lombard used a device called a thermoelectric apparatus, which allowed to conduct local temperature measurements when part of the scalp was compressed, leaving it pale and bloodless [15,16]. However, despite extensive trials, Lombard was unable to obtain any consistent results.

One important step beyond Broca and Lombard was already done by German physiologist Moritz Schiff, who conducted the first direct brain temperature recordings in awake animals in the late 1860s [17], long before the first recordings of the brain’s electrical activity or even the realization that neural cells have electrical activity. Schiff believed that brain activation induced by various sensory, motor, and “psychic” events should be accompanied by heat production that could be detected by local temperature change. Although the thermocouple needles of Bacquerel and Blanchet (1835; see [18]) utilized by Schiff were a unique tool used to detect the temperature changes, Schiff’s experiments did not hold modern scientific criteria because the thermocouple produced too small currents, recording galvanometers were primitive, most readings were subjective, with no statistical techniques to assert the validity of results. Despite all these shortcomings, the experiments of Schiff resulted in many important discoveries. Particularly, Schiff was able to detect brain temperature increases induced by various sensory (light, sound, smell) and motivational (food) stimuli. He also described the differences in brain temperature responses to the presentation of food to hungry and satiated dogs, showing that brain response depended on the animal’s motivational state (i.e. hunger). Among other pioneering findings was the observation of gradual diminishment of brain temperature responses following repeated presentation of sensory stimuli. He also observed a temperature response to sensory stimuli which was previously associated with food. These findings clearly predated Ivan Pavlov’s conditioning experiments initiated 20–30 years after Schiff’s temperature work. The work of Moritz Schiff was highly valued by Claude Bernard, another great mind of early physiology, who mentioned that “the results of recent experiments (of Schiff) do not leave doubts. “Each time the spinal cord or a nerve exhibit sensitivity or movement, each time the brain performs intellectual work, a corresponding amount of heat is produced” [19]. These findings as well as thermal recordings from the human scalp [15,16,20] allowed William James, the “father of American psychology”, to conclude that “brain-activity seems accompanied by a local disengagement of heat.” He also speculated that cerebral thermometry may be a valuable tool in experimental psychology, allowing one to correlate brain activity with psychic functions [21]. While clearly pioneering, further work in attempting to measure brain temperature non-invasively failed, but the work initiated by Schiff and his pupils can be now viewed as the early roots of functional brain imaging. Hans Berger, through Schiffs’s pupil Angelo Mosso, was able in 1924 to substitute thermocouple needles with electrodes, giving rise to electroencephalography (EEG) that became the leading technique to study brain activity for several decades. While Broca, Lombard, and Schiff strongly believed that changes in brain temperature reflect brain activity, Charles-Emile Francois-Frank was possibly the first to suggest that brain temperature changes observed in response to sensory stimuli are due to changes in cerebral circulation [22]. He also was the first to detect a dorso-ventral brain temperature gradient, with lower basal values in superficially located structures compared to deeply located structures. While these early findings were obviously significantly affected by heat outflow from thermocouple sensors, and the explanation of this gradient by the warming of deep structures by presumably warm arterial blood appears incorrect (see below), these studies underscore the importance of cerebral circulation as a critical contributor to physiological brain temperature fluctuations.

## Physiological brain temperature fluctuations: Causes and mechanisms

… When you can measure what you are speaking about, and express it in numbers, you know something about it; but when you cannot measure it, when you cannot express it in numbers, your knowledge is of an eager and unsatisfactory kind; it may be the beginning of knowledge, but you have scarcely in your thoughts advanced to the stage of Science, whatever the matter may be William Thompson, 1^st^ Baron Kelvin (1883) [23].

Despite the tight links between temperature and metabolism and temperature dependence of multiple neural processes, the pioneering experiments of Moritz Schiff and the ideas of William James were not reinforced at that time, although important reports utilizing brain thermorecordings sporadically appeared in the literature [24–30]. This is surprising because brain temperature recording in animals is not technically difficult and chronically implanted sensors allow for temperature monitoring with high temporal and spatial resolution in freely moving, behaving animals in multi-session experiments. The lack of interest in brain thermorecording could be related to the complexity in relating temperature as a basic physical parameter with different forms and manifestations of neural activity and conceptual mechanisms used to explain behavior (i.e. motivation, reinforcement, reward, punishment, etc.). The issue of brain temperature and its physiological fluctuations also stands at odds with the traditional ideas of thermoregulation, which view brain temperature as a stable or passively changing parameter. Furthermore, due to heat exchange both within the brain tissue and between the brain and rest of the body, brain temperature changes differ depending on temporal resolution; they show strong correlations when these parameters are determined with low temporal time resolution and important differences when analyzed with high, second-scale resolution.

Despite the general belief that brain temperature is a strictly regulated parameter which is maintained at constant levels, thermorecording studies conducted in rats, cats, dogs, and monkeys revealed relatively large brain temperature fluctuations (±2–3°C) that occur at stable ambient temperatures following exposure to various sensory stimuli, spontaneous changes in activity states, and during different motivated behaviors [25–31]. As shown in these studies, brain temperature fluctuations correlated with modality and biological significance (salience) of the environmental challenges, spontaneous and stimuli-induced changes in EEG and motor activity, and they had some structural specificity. While these findings suggest neural activity as the primary cause of brain temperature fluctuations, these fluctuations correlated in different brain structures and were typically associated with similar changes in core body temperature. Therefore, changes in brain temperature represent a physiological reality, which is dependent on metabolic neural activity but is quite different from other traditional measures of neural activity.

Our initial interest in brain thermorecording was initiated by three reasons. First, we thought that brain temperature could be an unexpected factor affecting the results of our early electrochemical studies, in which we tried to assess changes in extracellular dopamine (DA) levels associated with arousing stimuli [32], operant feeding behavior [33] and self-administration of heroin and cocaine [34,35]. While in these experiments we used highly DA-selective carbon-fiber sensors, changes in DA-dependent electrochemical signals showed relatively large tonic increases, which could not be explained by true increases in extracellular DA levels. Second, we know that psychostimulant drugs such as METH and MDMA induce robust body hyperthermia in both rats and humans. Therefore, we were interested in clarifying how these drugs affect brain temperature and in determining the relationships between changes in brain and body temperatures. Finally, we thought that brain thermorecording could provide a simple, sensitive, and integral index for the characterization of alterations in neural activity during motivated behavior. As an initial step to address these issues, we examined whether and how arousing stimuli of different modalities and salience affect brain temperature, what the differences in temperature responses in different brain structures and body locations are, and we sought to clarify the source of these temperature responses.

### Neural activation as a source of physiological brain hyperthermia

Since circulation is the primary means of heat exchange between the brain and the rest of the body, it was important to examine the relationships between temperature changes occurring in the brain and in its arterial blood supply. To accomplish this task, rats were chronically implanted with miniature thermocouple sensors in several brain structures (dorsal and ventral striatum, cerebellum) and in the abdominal aorta accessed via the caudal artery [36]. While the carotid artery seems to be the best measurement location, a catheter in this location significantly or fully blocks blood flow, thus resulting in undervalued measurements due to a cooler neck area. In contrast, the presence of a small-diameter catheter with a thermocouple sensor in the abdominal aorta does not result in an evident decrease in blood flow and the measured temperature could only be overvalued because of heat inflow from warmer surroundings (body core). Temperatures were recorded with high temporal resolutions in freely moving rats exposed to several physiologically relevant arousing stimuli (i.e. placement in the experimental cage, an auditory stimulus, social interaction of the recorded male with either a male or female companion, and tail-pinch). All measurements were conducted at stable ambient temperatures (22–23°C) and they were repeated during 5–6 daily sessions.

**Figure 1.**
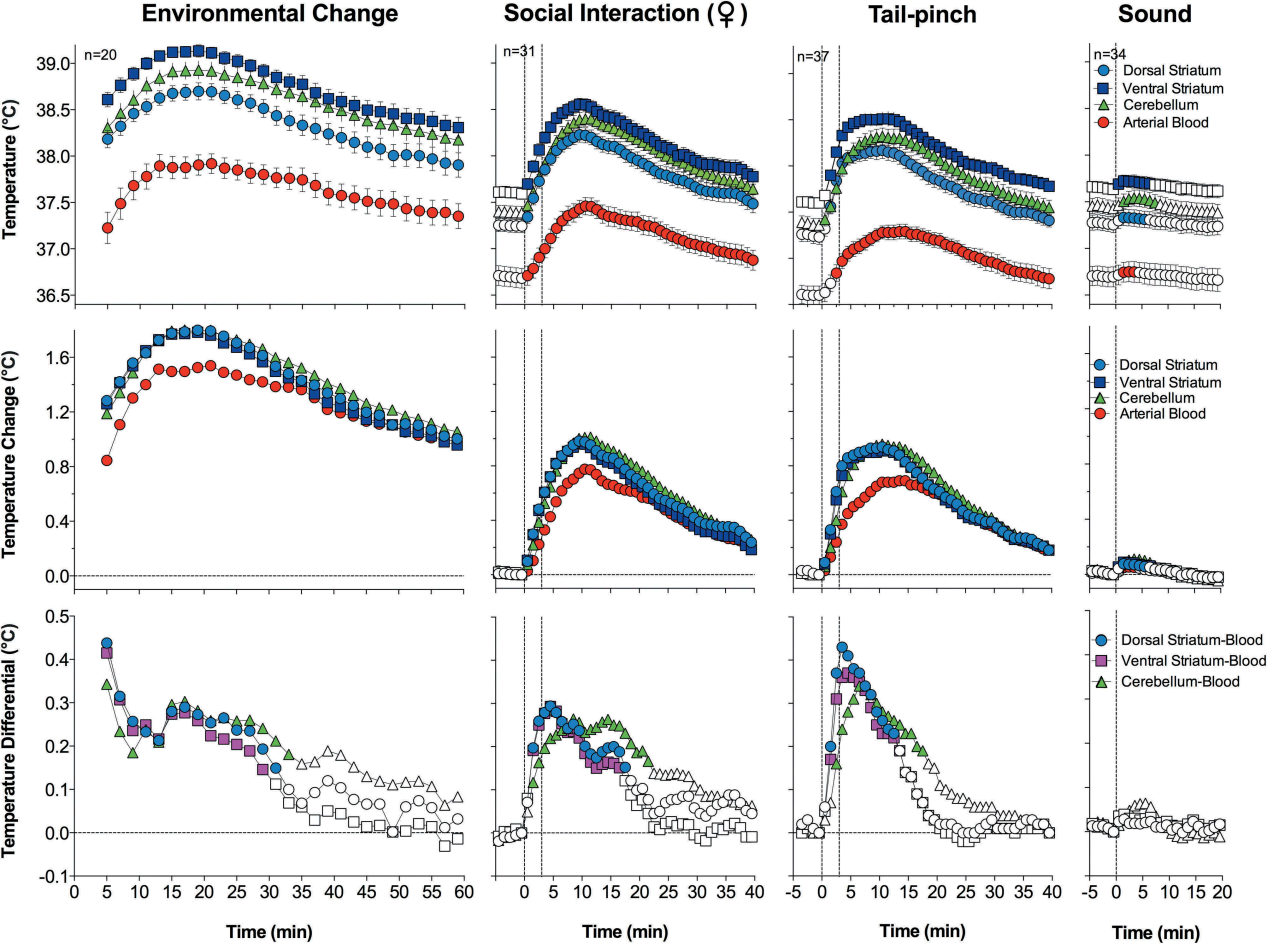
Mean (±SEM) changes in brain (ventral striatum, dorsal striatum, and cerebellum) and arterial blood temperatures induced by environmental change (animal transfer from the vivarium to the recording chamber), social interaction (3-min placement of a female rat into the chamber with recorded male), tail-pinch (3-min placement of wooden clamp on the tail base), and unexpected auditory stimulus (20-s tone) in freely moving male rats. A = absolute temperatures; B = relative temperature changes; C = brain-arterial blood temperature differences. Filled symbols indicate values significantly higher (Scheffe’s test following one-way ANOVA with repeated measures, p < 0.05) than baseline. Duration of stimuli is shown by two vertical hatched lines. The minimal value within each session, when the animal was at rest, was taken as baseline for the environmental change. Data were replotted from [36].

**Figure 2.**
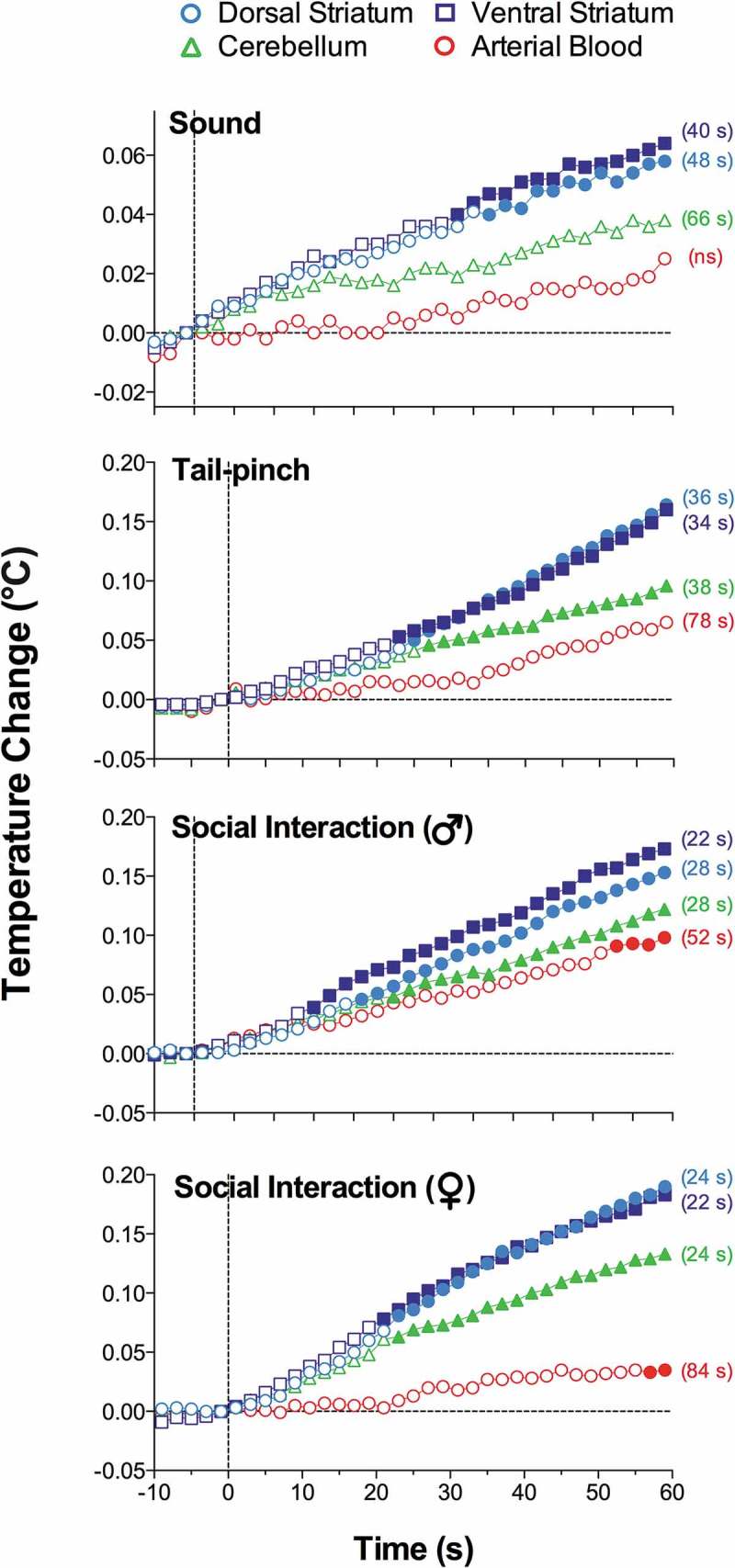
Onset latencies of temperature responses to different environmental challenges in brain structures and arterial blood in freely moving rats. Mean values are shown at 2-s intervals for 10 s before and 60 s after the onset of each stimulus. Filled symbols indicate values significantly (one-way ANOVA followed by Scheffe test, p < 0.05) higher than the last pre-stimulus value. Response latencies were defined as the first significant value vs. baseline and shown in brackets. Data were replotted from reference [36].

**Figure 3.**
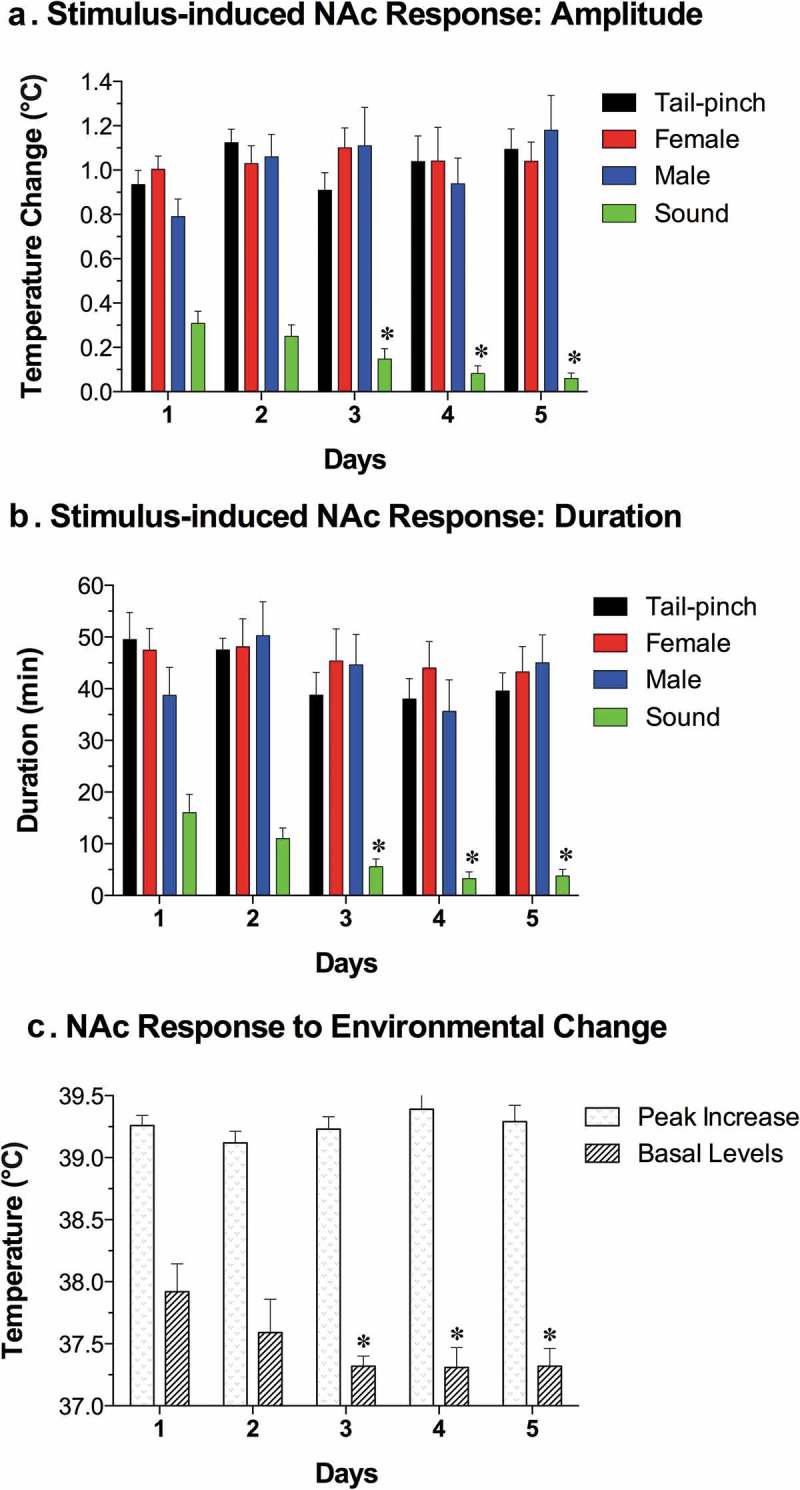
Day-to-day changes in NAc temperature increases induced by different environmental stimuli (tail-pinch, male-male interaction, male-female interaction, auditory stimulus). A = mean (±SEM) increases from baseline for each challenge condition on each day, B = mean (±SEM) duration of statistically significant increase evoked by each challenge on each day. C = mean (±SEM) values of maximal temperature increases during environmental change and mean values of minimal temperature (baseline) on each day. Asterisks mark values significantly different vs. Day 1 (Student’s t-test, p < 0.05). Data were replotted from reference [36].

As shown in this study, basal temperatures in all of the brain structures were significantly higher than that in the arterial blood (Figure 1). All stimuli increased temperature in each brain structure and in the arterial blood, and these increases greatly exceeded the duration of stimulation. While the response pattern was similar in all locations, temperature increases in all brain structures occurred significantly faster and were greater than those in arterial blood, resulting in a significant increase. When analyzed with high, 2-s time resolution, changes in brain temperature were surprisingly rapid in each structure and for each stimulus, showing increases within the first 10 s and significant levels within 20–30 s from the stimulus onset (Figure 2). In contrast, increases in arterial blood temperature were more delayed. Despite differences in basal temperatures, the temperature response was virtually identical in both the dorsal and ventral striatal sub-divisions, but temperature increases in the cerebellum were more delayed and prolonged following each stimulus. Environmental change – the transfer of the rat from the animal facility and its connection to the recording instruments–induced the largest temperature elevation, ~1.7°C vs. the lowest point occurring later within the session (Figure 1). When analyzed for five repeated sessions, brain temperature increases induced by tail-pinch, male-male, and male-female interactions were relatively stable, but the responses to a short auditory stimulus gradually weakened and completely disappeared by the fifth recording session (Figure 3(a–b)). Following repeated sessions, basal temperature decreased in each of the four recording locations both within a session and between sessions, reflecting animal habituation to the experimental environment. However, relative temperature increases elicited by salient stimuli (as shown for tail-pinch) remained relatively stable and differences between each brain structure and arterial blood always remained positive and relatively similar (Figure 3(c)).

Since the blood supply to the brain is cooler than the brain itself, and brain temperatures rise more quickly and to a greater extent than do arterial blood temperatures in response to all arousing stimuli, intra-brain heat production appears to be the primary cause of functional brain hyperthermia elicited by these stimuli. Intra-brain heat accumulation also depends on heat exchange with arterial blood. Therefore, the increased cerebral blood flow that occurs during neural activation (see below) not only brings oxygen and nutritive substances to the brain tissue, but also removes heat from it. As shown previously, tail-pinch induces an almost two-fold increase in striatal blood flow [37] and this increase is also rapid and greatly exceeds the duration of stimulation. Similar increases in striatal blood flow also occurred during grooming and eating [38], activities consistently associated with brain temperature increases [27,28,39]. Since arterial blood temperature also increases following arousing stimuli, the efficiency of heat outflow from the brain slowly decreases, resulting in more prolonged changes in brain temperature. Hence, brain circulation plays a significant role in the re-distribution of locally released heat within brain tissue and in its removal from the brain, thereby contributing to brain temperature fluctuations occurring under physiological conditions.

When we initiated our work with measurements of arterial blood temperature, we thought that these measurements were never conducted before and it appears that they were never done in rats. Further search of literature, however, revealed that arterial blood temperature using carotid artery access was previously recorded in dogs [24], cats [28,29], monkeys [40,41], and free-ranging ostriches and onyx [42,43].

While it seems mechanistic, brain-body temperature exchange could be analogous to an internal combustion engine. Similar to the coolant that continuously circulates and removes heat from a working engine, cooler arterial blood removes heat from the brain and delivers the now warmed venous blood to the heart and lungs to be cooled and oxygenated again in the lungs. Therefore, cerebral blood flow is critical for brain temperature homeostasis, and temperatures in the brain and the rest of the body are interdependent. While metabolism-related intra-brain heat production tends to increase brain temperature, temperature also rises when the brain-generated heat cannot be properly dissipated to the body and then to the external environment. Similarly, a decrease in cerebral metabolism tends to lower brain temperature, and this effect can be enhanced by peripheral vasodilatation that promotes heat dissipation to a cooler environment.

**Figure 4.**
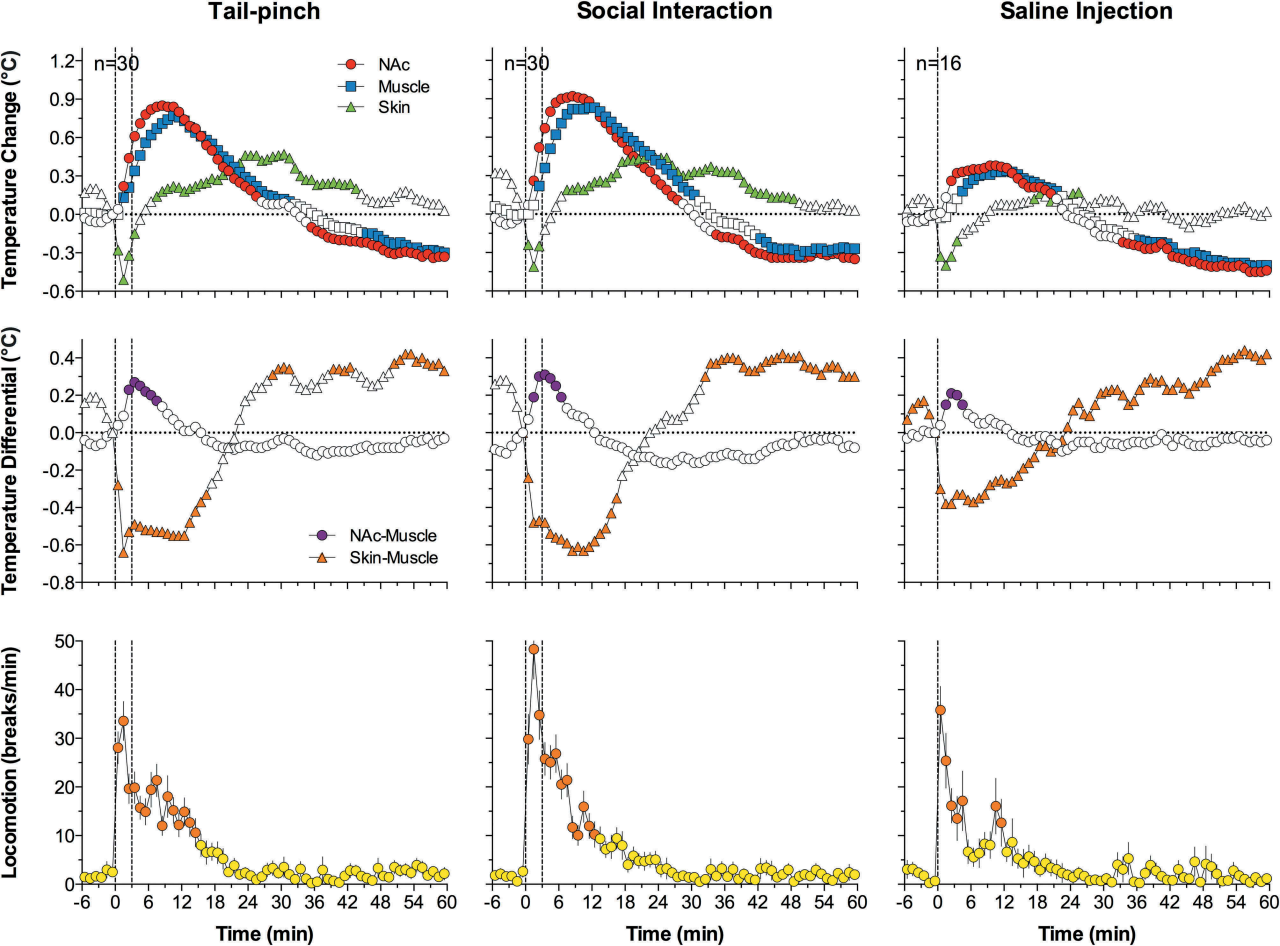
Changes in brain (nucleus accumbens or NAc), temporal muscle, and skin temperatures during tail-pinch, social interaction, and subcutaneous saline injection. A = relative temperature changes; B = brain-muscle and skin-muscle temperature differentials; C = locomotor activity. Duration of stimuli is shown by vertical hatched lines. Filled symbols indicate values significantly (one-way ANOVA followed by Fisher test, p < 0.05) higher than the last pre-stimulus value. Compiled from original data published in [57].

Due to technical complexities of temperature recording in arterial blood, in our later studies, in addition to brain sites, we recorded temperature in the temporal muscle, a non-locomotor head muscle that receives the same arterial blood supply as the brain from the carotid artery and thus is exposed to a similar temperature influence of arterial blood. By calculating the difference between temperature changes in a brain site and temporal muscle (brain-muscle differential), the latter influence from the periphery can be excluded, thus revealing the component related to intra-brain heat production. Figure 4 shows that temperature increases in the nucleus accumbens (NAc), a ventrally located brain structure critical for sensorimotor integration and a part of motivation-reinforcement circuit [44–46] induced by tail-pinch, social interaction and intraperitoneal (ip) saline injection were consistently more rapid and stronger than those in the temporal muscle resembling the changes seen in arterial blood (compare with Figure 1). Similarly, the NAc-muscle temperature differential rapidly increased at the start of arousing stimulation, paralleling the ascending portion of the temperature curve.

While the rise of temperature differential between the brain site and arterial blood or temporal muscle consistently induced by arousing stimuli could provide a measure of brain metabolic activation, a similar change occurs in the brown fat, another factor contributing to body thermogenesis [47,48]. Under normothermic conditions, temperature in brain fat tissue is slightly lower than in body core, but it is consistently increased during environmental cooling and exposure to pyrogens, becoming up to one degree higher than in body core ([49,50]. While heat production in brown fat tissue is an important factor in body hyperthermia during environmental cooling and inflammation-induced fever, its contribution to physiological brain and body hyperthermia appears limited due to small mass of brown fat tissue (0.1–1% in humans [51,52] and less than 1% in rats [53]).

Since brain temperature also depends on peripheral heat loss, to assess the contribution of this factor, we recorded skin temperature by a sensor chronically implanted in the subcutaneous space in frontal facial area, a densely vascularized area with no or minimal metabolic activity of its own. In contrast to brain and muscle sites, skin temperature during tail-pinch and social interaction showed a biphasic fluctuation, with an initial rapid drop followed by a more prolonged tonic increase (Figure 4). Since skin temperature depends on two factors – the tone of blood vessels and temperature of incoming arterial blood – we calculated skin-muscle temperature differentials that allowed us to exclude the contribution of the latter factor and provide an accurate measure of changes in skin vascular tone. Skin-muscle differentials rapidly decreased after the start of arousing stimulation (Figure 4), reflecting peripheral vasoconstriction – a known adaptive response triggered by various stressful stimuli [54–56] and aimed to retain heat under conditions of potential danger. Skin temperature response resulting from vasoconstriction was the most rapid, typically appearing during the first 10–20 s after the start of stimulation and correlating with locomotor activation (Figure 4). It was also the most transient, and it typically inverted into rebound-like skin warming, suggesting enhanced heat dissipation that follows heat retention induced by arousing stimuli. Therefore, skin vasoconstriction that diminishes heat loss to the external environment is another essential contributor to physiological brain hyperthermia.

As shown in Figure 4, ip injection of saline conducted in wake, quietly resting rats induced a transient hyperthermic response, which was weaker, but qualitatively similar to those elicited by arousing stimuli such as tail-pinch and social interaction. Similar data were also obtained for subcutaneous (sc) saline injection. These findings suggest that the procedure of injection is an arousing stimulus and these temperature changes should be considered when interpreting temperature responses induced by drugs, which are often administered via ip or sc injection.

**Figure 5.**
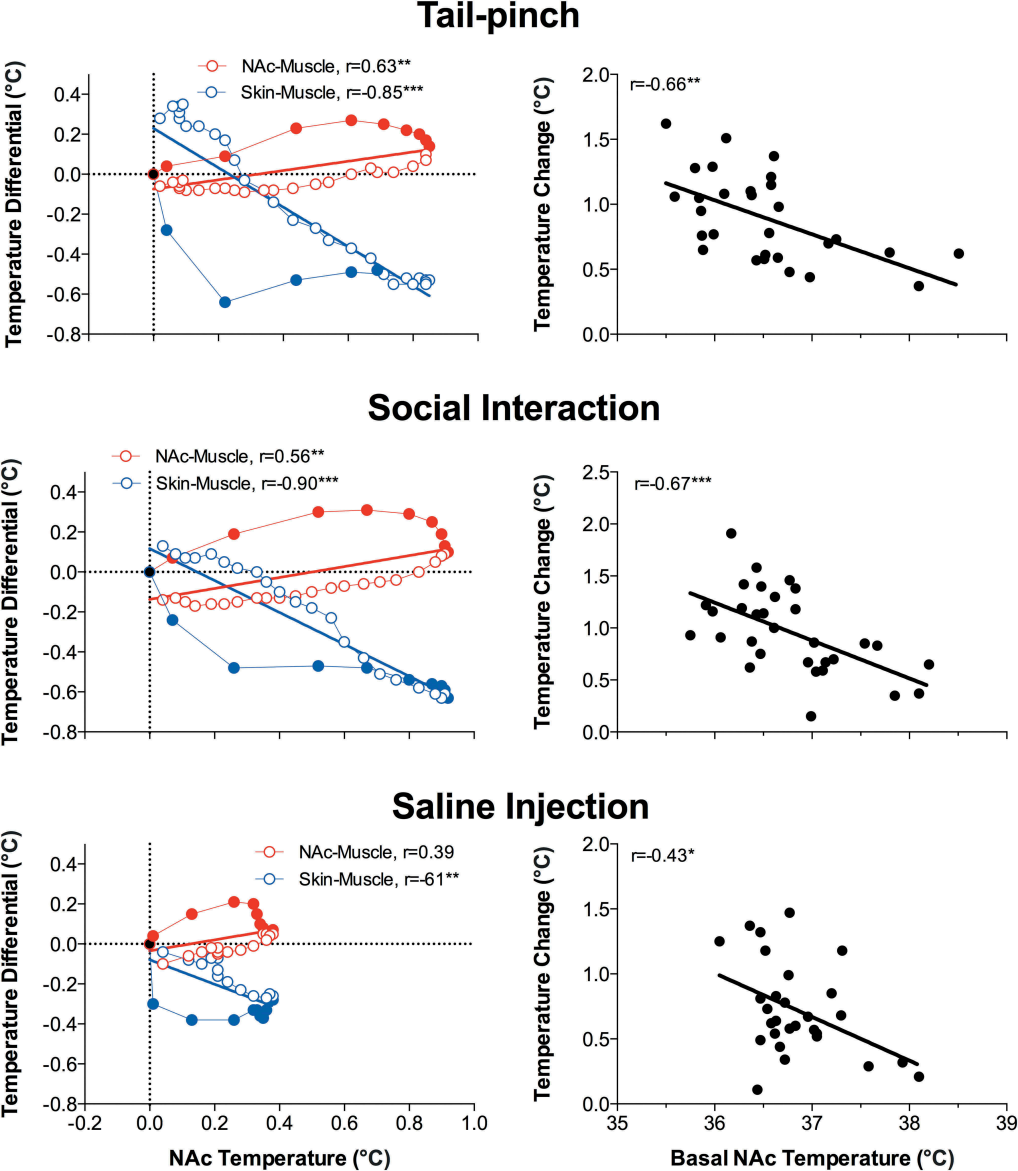
Relationships between changes in NAc temperature and two temperature differentials (NAc-muscle and skin-muscle) during tail-pinch, social interaction and subcutaneous saline injection in freely moving rats. Graphs on the left side show time-dependent correlative relationships between changes in NAc temperature and two temperature differentials. In each case, increases in NAc temperature correlated with increases in NAc-muscle differentials and decreases in skin-muscle differentials. Graphs on the right side show that NAc temperature increases induced by each arousing stimulus were dependent on basal NAc temperatures; these responses were strong at low basal temperatures and progressively weaker at higher basal temperatures. Regression line crossed the line of no effect at ~39.0–39.5°C, suggesting upper limits for physiological brain temperature increase. Compiled from original data published in [57] and re-analyzed for this report.

Since two factors – an increase in intra-brain heat production and decrease in heat loss – determine brain temperature increases induced by arousing stimuli, we used time-dependent correlation to examine relationships between changes in brain temperature and changes in both brain-muscle and skin-muscle differentials. As shown in Figure 5 (left panel), for all three stimuli analyzed, the NAc-muscle differential positively correlated with brain temperature. A significant but much stronger negative correlation was found for brain temperature and the skin-muscle differential, which rapidly decreased within 2–3 min after stimulus onset. Therefore, decreased heat dissipation due to skin vasoconstriction is a stronger factor for brain temperature increases induced by arousing stimuli.

An important feature of brain and body temperature responses elicited by arousing stimuli is their dependence on basal temperatures (Figure 5, right panel). In each case, temperature increase was large when basal temperature was low, and the increase became gradually weaker when basal temperature was higher. In each case, correlation was significant and regression lines crossed the line of no effect at 38–39°C, suggesting that the response should disappear at high basal temperatures. However, such high temperatures are within the range of physiological fluctuations, occurring, for example, during the rats’ placement in novel environments [58] or during natural copulatory behavior [59] (see below). Importantly, this correlation appears to be valid for any arousing stimulus, reflecting a basic relationship between basal activity state (basal arousal) and its changes induced by environmental stimuli. However, this correlation is quantitatively weaker for stimuli with low activating potential and smaller temperature responses and stronger for powerful stimuli that induce larger temperature elevations. These observations may be viewed as an example of Joseph Wilder’s “law of initial values” that postulates that the magnitude and even direction of autonomic response to an activating stimulus depend on the pre-stimulus basal values [60,61].

Although different brain structures show a generally similar pattern of temperature fluctuations following exposure to arousing stimuli, their basal temperatures differ significantly. Specifically, more dorsally located structures have been found to be consistently cooler than more ventrally located structures. While these findings support a dorso-ventral temperature gradient first described in animal brains [26,28,62] and later demonstrated in humans [63–65], the underlying mechanisms of this gradient are still a matter of discussion. It was suggested that higher temperature in more ventrally located structures reflects their farther location from the colder environment and greater heating by warm blood from the body [12,66,67]. However, this assumption, inferred from rectal or core body measurements, was challenged by direct measurements of arterial blood temperatures [29,36,68], which revealed that the brain is always warmer than the arterial blood supply and thus cannot be warmed by arterial blood. Alternatively, a dorso-ventral temperature gradient could be related to differences in metabolic activity, particularly in structure-specific expression of brain uncoupling proteins that regulate uncoupling in mitochondria and local heat production [62]. This temperature differential could also be related to neuronal activity. The great majority of neural cells in the “colder” cortex (>95–98%) are silent at rest, but they are phasically excited following sensory stimulation [69,70]. The same is true for dorsal striatal cells, most of which (at least 90–95%) are electrically inactive in awake, freely moving rats at rest but show phasic stimuli-induced excitations [71,72]. In contrast, most neurons of the “warm” hypothalamus and ventral tegmental area of midbrain are spontaneously active at rest [73,74]. While these data suggest that structure-specific differences in neuronal activity can be a factor determining structure-specific differences in brain temperature, direct neuronal data from different structures in awake animals are very limited and this link cannot be yet substantiated. Finally, it could not be excluded that dorso-ventral brain temperature differential, especially in small animals, may be partially dependent on technical aspects of thermorecording. When the tip of a temperature-sensitive metal probe is not in tight contact with brain tissue or is implanted superficially, heat dissipation from the probe to the external environment may be greater and measured temperatures will be undervalued. Even greater recording errors could occur when temperatures are measured from an open skull.

Neural activation associated with intra-brain heat production appears to be the primary cause for physiological increases in brain temperature, but neural activation also results in increases in cerebral circulation (see [75,76] for review) that enhance heat dissipation from brain tissue. While temperature is usually either omitted from equations relating to metabolism and blood flow or viewed as a passive parameter [77,78], it can also actively affect cerebral blood flow. Direct correlation between temperature and local blood flow was shown in both multiple peripheral tissues (i.e. skin, muscle, intestine, and liver [79–83]; and in the brains of rats, monkeys, and humans [84–86]. Therefore, increases in local brain temperature can be viewed as a factor that enhances cerebral vasodilation and increases local blood flow. This inter-dependent interaction can explain, at least partially, the well-known but not clearly explained phenomenon of greater blood flow increases that exceed the metabolic activity of brain tissue [75]. Due to this mechanism, more oxygen and nutrients are delivered to areas of potential metabolic demand and more potentially dangerous heat is removed from active brain tissue, thus providing a crucial advantage for successful goal-directed behavior and the organism’s adaptation to energy demands.

### Brain temperature fluctuations during motivated behaviors

After establishing the general pattern of changes in brain temperature elicited by salient sensory stimuli and clarifying their basic mechanisms, we applied multi-point thermorecording to three types of natural motivated behavior (male-female sexual interaction, feeding and drinking behaviors). While our experimental goal was to examine temperature fluctuations associated with critical events and manifestations of each behavior, our general goal was to use these data to understand basic changes in neural activity underlying motivated behaviors.

#### Brain hyperthermia and phasic temperature fluctuations during male and female sexual behavior

Sexual behavior is perhaps the most important type of social interaction and the most coordinated and energy-consuming form of natural goal-directed behavior. It is also a unique behavior that involves interaction of two subjects of the opposite sex. In our experiments, we used multi-site thermorecording to determine common features and between-sex differences in temperature dynamics in sexually experienced male and female rats during exposure to sexually arousing stimuli (smell of an opposite-sex partner and emitted sounds) and subsequent copulatory behavior [59,87]. Recordings were taken from three brain sites: medial preoptic area of hypothalamus (mPOA), a structure implicated in central regulation of both sexual functions [88,89] and thermoregulation [2,4], the NAc, a structure crucial for any motivated behavior [44,90], and the hippocampus (Hippo), a brain structure usually not implicated in central organization of either sexual behavior or thermoregulation. Deep temporal muscle was the fourth recording location, thus enabling us to determine brain-muscle temperature differentials, a means by which metabolic brain activation can be represented (see above). Since this study was historically our first behavioral work with thermorecording and we were limited to the four recording channels, we did not employ skin recordings in this study. We used an identical protocol, which involved the exposure of the recorded, sexually experienced animal (either male or receptive female) to the sexual partner (either receptive female or male). First, the partner was placed into one compartment of a two-compartment chamber with an opaque barrier separating two animals. Then, the opaque barrier was removed but rats remained physically separated from each other by a transparent barrier with holes in it. This allowed limited interaction with the opposite sex partner. Finally, the last barrier was removed and rats initiated sexual behavior, which consisted of 3–5 relatively uniform copulatory cycles. One hour after full cessation of copulatory behavior, the partner was removed from the cage.

**Figure 6.**
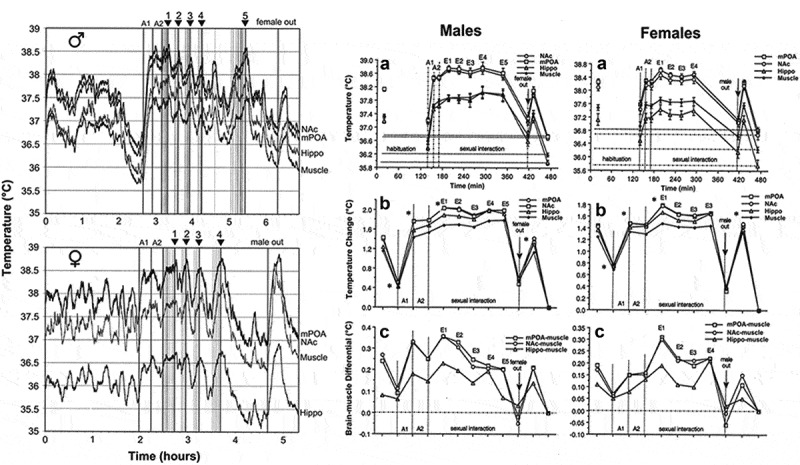
Temperature changes in three brain structures (medial-preoptic hypothalamus or mPOA, nucleus accumbens or NAc, and hippocampus or Hippo) and temporal muscle during sexual interaction session in male and female rats. Left panel shows original records of temperature fluctuations in each recording area in representative male and female rat. A1 and A2 are arousal I and II, respectively. Vertical lines show mounts and intromissions, black triangles with numbers show moments of ejaculation, “female out” depicts the moment when sexual companion was removed from the cage. Right panel shows mean values of absolute temperatures in each recording location (A), relative temperature changes (B), and brain-muscle temperature differentials (C) during sexual interaction session in male and female rats. Asterisks on B show values significantly different (ANOVA with repeated measures followed by Scheffe F-test, p < 0.05) from the previous value. Original data were published in [59,87] and replotted for this article.

Figure 6 shows representative original examples of temperature changes recorded in male and female rats during the entire experiment (left panel) and mean values of temperatures in each recording location during each experimental event of sexual interaction: placement of the rat in the cage at the start of experimentation, stabilization of temperatures 2 h later, presentation of an opposite-sex rat in the neighboring compartment (arousal A1), removal of the opaque barrier separating the animals (arousal A2), each subsequent male’s ejaculation during sexual interaction (E), and removal of the partner from the cage (right panel).

As can be seen in these figures, sexual interaction in both males and females was accompanied by robust tonic increases and phasic fluctuations in brain and muscle temperature. Relative to the baseline established 2 h after the recorded animal was placed in the cage (habituation), temperatures rapidly increased (+1.5–2.0°C) when the opposite-sex animal was placed into the neighboring compartment, remained tonically elevated (38–39°C) during subsequent sexual interaction, and decreased after its termination. Temperature changes in males and females were surprisingly similar in each of the four recording sites, but elevations in brain structures were larger than those in the temporal muscle, resulting in significant increases in brain-muscle differentials that persisted during the entire period of sexual interaction (Figure 6(c)). The increases in this parameter were strong and almost identical in the mPOA and NAc and lower in the Hippo, and they were maximal at the moment of the male’s first ejaculation (E1 in Figure 6(c)). Similar to the introduction of an opposite-sex partner (A1), removal of the partner was associated with a new temperature increase and a significant rise in the brain-muscle differential (male and female out in Figure 6). Although males and females showed the same between-site differences in temperatures, females had higher basal temperatures in each location after their placement in the cage of previous sexual interaction, and higher basal values than males at the start of arousing stimulation. Because of these differences, female rats showed a weaker relative temperature increases during arousing stimulation and subsequent copulatory behavior than males. Finally, in animals of both sexes, temperature values established after complete cessation of sexual behavior were the minimal points within a session, obviously representing a true quiet resting baseline (zero in Figure 6(b)). With respect to this baseline, temperatures before the start of arousing stimulation were significantly elevated in both males and females and this elevation was larger in females.

**Figure 7.**
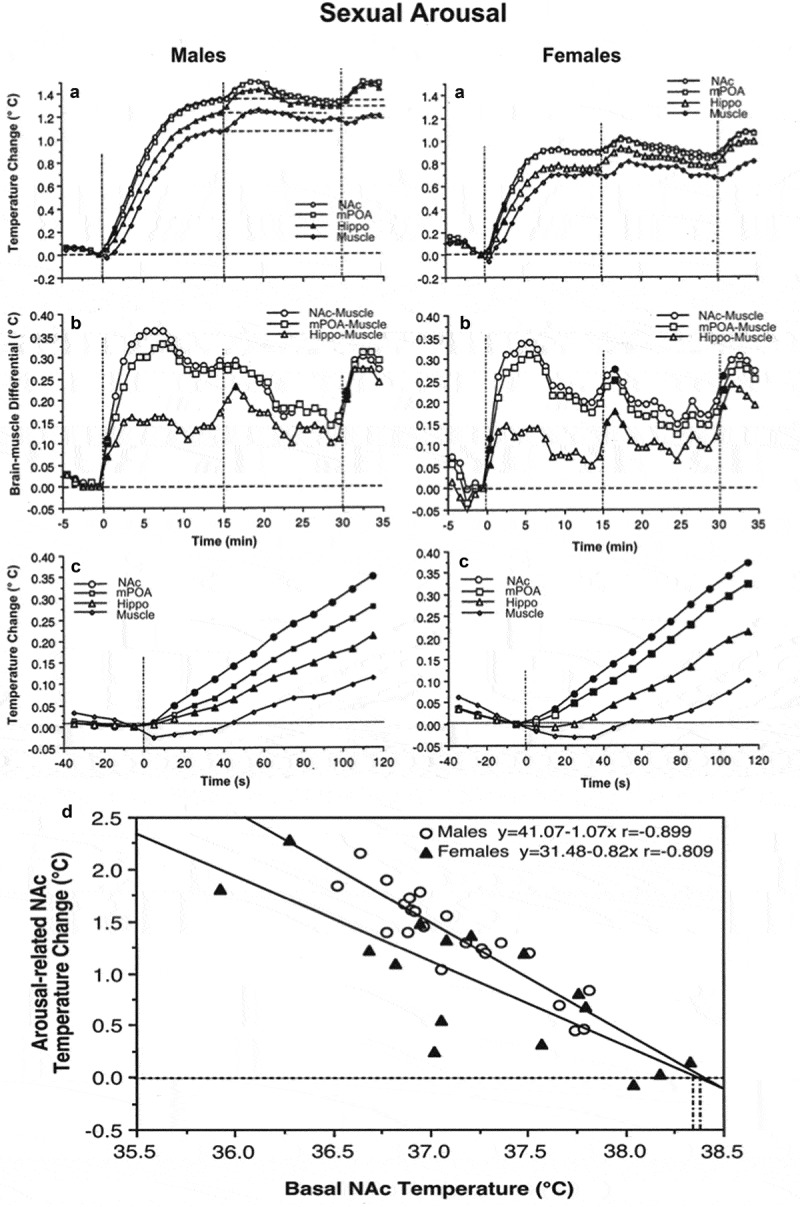
Phasic changes in brain (NAc, mPOA, and Hippo) and muscle temperatures in male and female rats associated with sexual arousal preceding copulatory behavior. A and B = changes in relative temperatures and brain-muscle differentials for the 35 min after the start of sexual stimulation (vertical hatched lines show arousal 1, 2, and time of free interaction). Filled symbols show the first value significantly different (ANOVA with repeated measures followed by Scheffe test, p < 0.05) from baseline for each of three comparisons (arousal 1, arousal 2, and free interaction). C shows the initial period of stimulation (10-s bins for 120 s). In this case, filled symbols indicate all values significantly different from baseline. Bottom graph shows correlative relationships between the magnitude of NAc temperature increase induced by sexually arousing stimuli in male and female rats and basal temperatures. In both cases, correlation was highly significant (p < 0.01). Regression lines for both males and females cross the line of no effect at ~38.4°C, suggesting that the arousal-related brain temperature increase has its natural limits and disappears at high activity state. Data were obtained in 32 (seven rats) and 17 sessions (five rats) in males and females, respectively. Original data were published in [59,87] and replotted for this article.

Rapid time-course analysis provided a more precise picture of rapid temperature fluctuations (Figure 7). In both males and females, temperature rapidly and strongly rose and brain-muscle differentials significantly increased in each recording location after a partner was placed behind a nontransparent barrier, transiently increased again when the first barrier was removed, and slightly increased again after the rats began to interact freely. The NAc showed the shortest onset latencies (10–20 s) and most rapid acceleration of the initial temperature increase, mPOA was slightly less active, and Hippo showed the smallest change (Figure 7C). The longest latency and a minimal increase were seen in the temporal muscle; this increase was preceded by a transient decrease (presumably because of acute vasoconstriction (see [56,91] immediately after the onset of stimulation. The time-course of arousal-related temperature elevation was similar in both sexes, but the magnitude of elevation was larger in males (~1.4°C) than females (~0.9°C), because of higher baseline temperatures in females (see Figure 6).

Similar to other sensory stimuli, the magnitude of brain temperature elevation elicited by sexually arousing stimuli in both males and females was strongly dependent on basal temperatures (Figure 7(d)). When the temperatures at the start of arousing stimulation were low, the increase was large (1.6–2.2°C), while it was smaller (0.6–0.8°C) when the rat was more active and basal temperatures were higher. This dependence of the response on pre-stimulus baselines may be responsible for between-sex differences in relative amplitude of arousal response (Figure 6), which was stronger in males and weaker in females, which had higher temperatures than the males at the start of arousing stimulation. In animals of both sex regression lines cross the line of no effect at ~38.4°C (Figure 7(d)), suggesting that arousal-related temperature elevation has its natural limits; these values were virtually identical to the tonic temperature plateau that was maintained during copulatory behavior (see Figure 6). This relationship again is consistent with Wilder’s law of initial values [60,61] and appears to be common for any arousing stimulus.

**Figure 8.**
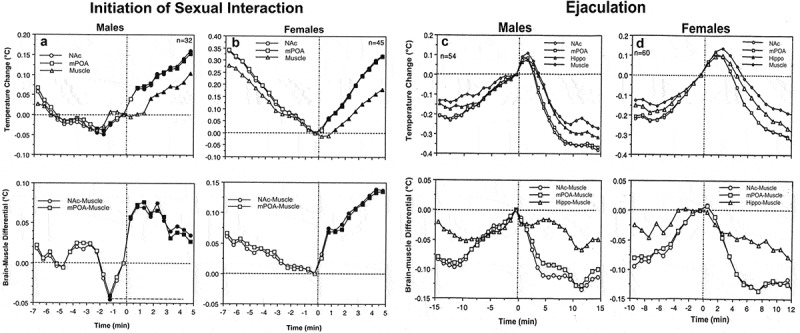
Changes in brain (NAc and MPAH) and temporal muscle temperatures in male and female rats associated with the initiation of copulatory behavior (A and B) and ejaculation (C and D). Left-side graphs show changes in temperature and brain-muscle differentials preceding and following the first mount/intromission of a session (= 0°C). Filled symbols show values significantly larger (ANOVA with repeated measuresfollowed by Fisher test, p < 0.05) from the pre-mount baseline. In males, temperature increase and rise of brain-muscle differentials began ~2 min before the mount/intromission, but in females, it occurred after this event. Right-side graphs show mean changes in temperature and brain-muscle differentials preceding and following ejaculation (= 0°C) he first mount/intromission of a session (= 0°C). Filled symbols indicate values significantly different (ANOVA with repeated measures followed by Fisher test) from the last pre-ejaculation value. n = the number of averaged events. Original data were published in [59,87] and replotted for this article.

In contrast to a similar pattern of temperature increases induced by sexually arousing stimuli in males and females, there were between-sex differences in rapid temperature fluctuations associated with the initiation of copulatory behavior (Figure 8(a-b)). In males, temperature increases and a rise in brain-muscle differentials began about 2 min *before* the first mount or intromission, peaked at the time of this event, and steadily continued during subsequent copulatory behavior. In contrast, brain temperature in females began to increase only *after* the first sexual contact, sharply accelerating after this event. Interestingly, in males, there were no differences in absolute temperatures and their relative changes between the first mount and intromission, but in females that allowed intromission as the first sexual contact, brain temperatures were significantly higher and brain-muscle differences were larger than those for cases when the copulatory cycle began with a mount. Although an anticipatory temperature elevation that precedes the initial sexual contact supports the known active role of the male rat in sexual interaction, the female’s activity state appears to be an important “permissive” factor determining the type of sexual contact occurring in the males. When females are at lower activity states they allow males to mount, but when their activity state increases they allow intromission. Importantly, successful sexual interaction in rats is possible only when the female is in a state of sexual receptivity, which is associated with higher behavioral activity, higher brain temperatures, and larger brain-muscle differentials than those in a non-receptive state.

In contrast to monophasic temperature increases triggered by sexually arousing stimuli and occurring following the initial mount/intromission, subsequent copulatory cycles were associated with biphasic temperature fluctuations. These fluctuations were smaller in magnitude (~0.3–0.6°C), peaked around ejaculations, and were superimposed on a tonic elevation that persisted during the entire period of sexual interaction (Figure 8(c–d)). In both males and females, temperature gradually increased during repeated mounts and intromissions, peaked within 1–3 min after ejaculation (zero time), and abruptly decreased to a lower point during post-ejaculatory inactivity from which temperature increased again during the next copulatory cycle (Figure 8(c–d)). Males and females also showed similar structural differences, with maximal and similar temperature fluctuations in the NAc and mPOA, smaller fluctuations in the Hippo, and minimal changes in the muscle. In males, the moment of ejaculation was associated with a dramatic temperature rise that was followed by a strong temperature fall. A similar biphasic change occurred in females, but the increase was more gradual peaking at 2–3 min following male ejaculation (Figure 8(d)). Between-sex differences were also seen in dynamics of brain-muscle differentials (Figure 8(c)). This parameter in males gradually increased and peaked within the last minute *preceding* ejaculation, but in females, it peaked during the first minute *following* the male’s ejaculation. While the post-ejaculatory decrease in brain-muscle differentials suggests an abrupt cessation of previous activation, brain temperature continued to increase for about 1 min after ejaculation, during the period when the male rat was in deep hypoactivity (refractory period). Since muscle temperature, in this case, continued to increase for about one more minute after brain temperature began to decline, the brain-muscle differential offered us a view of the actual peak of neuronal activation, which in male rats occurred immediately prior to ejaculation. The first-in-session ejaculation was the point of maximal temperature increase and maximal brain-muscle difference in both males and females. At this point, mPOA temperature ranged between 38.1 and 39.9°C in males and 37.8 and 39.4°C in females. During subsequent copulatory cycles, these values slightly decreased and the post-ejaculatory temperature decrease became more pronounced until the last ejaculation, after which the animals ceased any further sexual activity and temperature decreased toward baseline. Similar biphasic brain temperature fluctuations associated with male ejaculation were also found in the study of Mark Blumberg and collaborates [92]. In this study, mPOA temperature consistently increased during mounts and intromissions, peaked at the minute of ejaculation (38.5–39.5°C), and rapidly dropped within a minute following ejaculation.

These dynamics of temperature fluctuations found in rats match the results of human physiological studies, which have revealed the most dramatic increases in cardiovascular activity, breathing, and oxygen consumption as occurring preceding male’s ejaculation and rapid decreases in these parameters during post-ejaculatory inactivity [93–101]. Our findings are also consistent with single-unit data obtained in male rats. In the mPOA, >90% of neurons showed sustained excitation, which began after the introduction of a female rat, persisted during the entire period of sexual behavior, and abruptly terminated immediately after ejaculation [102]. In addition, many mPOA neurons showed phasic activations associated with pursuit of the female and rapid transient activations associated with mounts and intromissions.

Despite different roles attributed to each brain structure, the NAc and mPOA showed very similar temperature changes associated with critical events of sexual behavior. In contrast, the Hippo was less active during copulatory behavior, but showed the strongest activation during the second stage of sexual arousal, when rats were engaged in nose-to-nose interactions via holes in the dividing barrier. While these differences could reflect specific functional roles of each structure, intra-brain heat exchange would significantly contribute to a generally similar pattern of between-structure temperature fluctuations. A basic similarity of brain temperature fluctuations found in male and female rats is also consistent with electrophysiological findings. For example, recordings of mPOA neurons in male rats revealed that many of these cells showed mount- and intromission-related excitations, which were superimposed over a profound tonic activation [103,104]. Similar highly phasic changes in neuronal activity at different events of precopulatory and copulatory behavior were obtained in medium spiny NAc neurons [105]. Importantly, a similar pattern of neuronal activity was found in other brain structures, particularly in the lateral midbrain tegmentum [104] – a distinct area with an unclear role in sexual behavior. Microdialysis assessments of NAc dopamine during sexual behavior in male and female rats also mimicked this pattern [106,107] with robust increases during sexual arousal and decreases following ejaculation.

The pattern of brain temperature fluctuations during copulatory behavior was generally similar during repeated sessions. However, there were also some day-to-day differences. While in control animals placed repeatedly in the same chamber, NAc temperatures stabilized at relatively low baseline levels with a tendency to slightly decrease across days, these changes were slowed and then inverted in sexually experienced rats placed in the cage of previous sexual interaction. Both in males and especially in females, basal NAc temperature before the start of sexual stimulation was higher, with an almost 1.0°C differences from intact control animals. These changes obviously result from conditioned brain activation developed following association of the specific environment with the previous successful copulatory behavior (learning). While in males the diminished habituation to the environment of previous sexual interaction may be explained exclusively by a result of associative learning [108], in females hormonal stimulation used to induce sexual receptivity may be an important contributor to behavioral hyperactivity and higher temperatures at the start of sexual interaction [109].

#### Feeding behavior

Early thermorecording studies revealed that feeding behavior is accompanied by an increase in brain temperature, which is evident in different structures and generally correlates with core body temperature [27,28]. Since feeding behavior involves several prerequisites and multiple components (deprivation state, food availability, food-seeking activity, eating), it remains unclear which components of this behavior are associated with brain temperature increases and decreases, what the mechanisms of these changes are, and what the functional significance of these temperature fluctuations is.

To answer these questions, we developed a simple paradigm of feeding behavior, in which we conducted repeated high-resolution three-point thermorecordings (NAc, temporal muscle, and skin) in the same rats, which were either food-deprived (for 24 h) or satiated, and which exhibited different behaviors depending on food accessibility [39]. Before sensor implantation, rats were pre-trained to consume food from a presented plastic container (that allowed rats to see and smell the food) and only rats that showed consistent eating behavior were used in thermorecording experiments. During temperature recordings, we presented rats with the same food container, which could be either open, allowing the rats to obtain the food (1.5 g of regular food), or closed, not allowing direct contact with the food despite their continuous food-seeking activity. Our goal was to answer the following questions: (1) Whether and how is food deprivation reflected in basal locomotor activity and basal temperatures and how does it affect an animal’s behavioral response to food-related stimuli? (2) What changes in temperature and general locomotor activity occur during eating?(3) How do these changes differ from those occurring during food-seeking activity when the food is inaccessible? Finally, (4) What clues can brain thermorecording data provide to understand the organization and regulation of motivated behavior?

If an animal is deprived of food, it may be assumed that it differs from a non-deprived animal by its motivational state, which should be reflected in physiological and behavioral parameters. However, comparison of basal temperature and locomotion data from food-deprived and non-deprived rats did not reveal any significant differences, although all values were lower in food-deprived rats. While this may seem surprising, this finding is consistent with other data suggesting that food deprivation (starvation, fasting) slightly decreases whole-body metabolism, resulting in lower spontaneous locomotion and lower brain and muscle temperatures [110–112]. Under these conditions, peripheral vessels are to some extent constricted, resulting in lower skin temperature, thus retaining heat inside of the body by limiting its dissipation to the external environment.

When rats were satiated, they usually did not show any specific interest to the presented food container, but food-deprived rats consistently demonstrated food-seeking activity, manifesting as active interaction with the container. When the container was open, rats removed food from the container and consumed it, but when the container was closed, they continued their food-seeking behavior, trying to obtain food from it.

**Figure 9.**
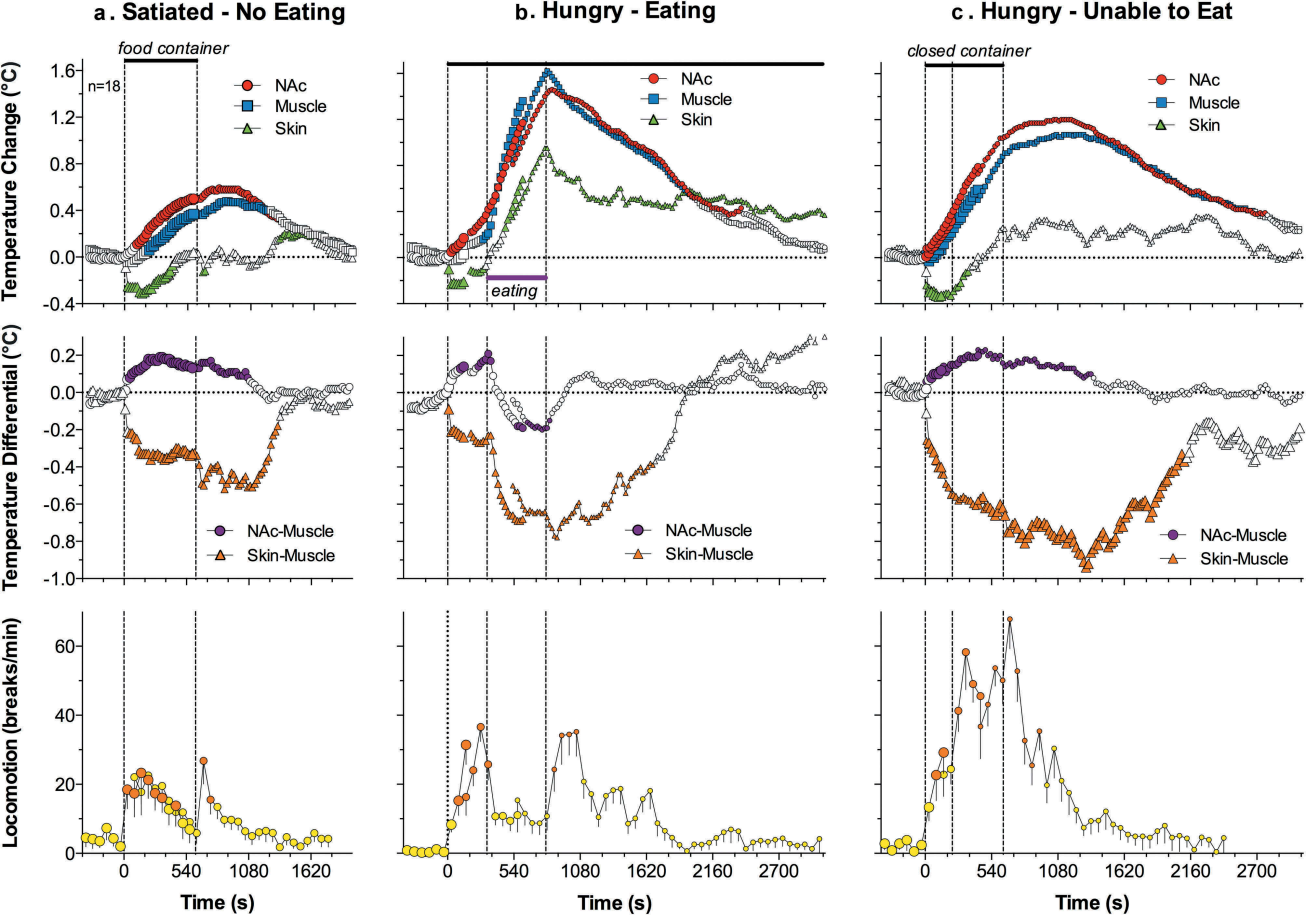
Mean changes in temperatures (NAc, temporal muscle, and skin; top row), temperature differentials (middle row) and locomotion (bottom row) during three feeding experiments in trained rats. In the first experiment (A), the rat was satiated and did not show eating during the presentation of food container (two vertical lines = presentation and removal of food container). In the second experiment, the rat was food-deprived (hungry) and it reached food container, removed food sample, and consumed it. In the third experiment, the rat was food-deprived (hungry) but the food container was closed and the rat was unable to retrieve food from the container. Original data were reported in [39] and replotted for this article.

Although *satiated rats did not actively interact with the presented container*, its placement in the cage moderately increased NAc and muscle temperatures, decreased skin temperature, and induced modest locomotor activation (Figure 9(a)). Both locomotion and temperatures increased again when the container was removed from the cage 10 min after its presentation (second vertical line in Figure 9(a)); these secondary increases were clearly weaker than those associated with the container’s presentation. Temperature increases in the NAc were rapider and larger in amplitude than those in the muscle, resulting in a significant rise in the NAc-muscle temperature differential. In contrast, the skin-muscle differential rapidly decreased and was maintained at lower values for about 20 min after container presentation. Similar to temperatures, both differentials showed a small secondary change when the food container was removed from the cage. Therefore, temperature responses elicited by food presentation in satiated rats mimic the pattern of changes induced by other arousing stimuli.

Temperature responses elicited by the presentation of the food container to hungry rats were quite different and depended on the rat’s ability to retrieve food and consume it. While hungry rats always actively interacted with the food container, they rapidly retrieved food from an open container and began eating when container was open but continued to interact with the closed container when they were unable to retrieve food. Presentation of a food container (first vertical line in Figure 9(b,c) in both cases resulted in similar temperature responses: a gradual increase in brain and muscle temperatures, rapid decrease in skin temperature, and a gradual increase in locomotion. Temperatures further increased when the rat retrieved food from the open container and started eating. In this case, muscle temperature showed the most rapid increase, clearly exceeding that in the NAc and skin. These differential changes resulted in a robust decrease in the NAc-muscle and skin-muscle temperature differentials. After eating was completed, temperatures in all recording locations rapidly decreased and both differentials slowly returned to their baselines. Behaviorally, after eating was completed, rats exhibited intensive grooming, washing, and rearing. These changes were significantly greater than those seen in satiated rats, which showed no interest in the food (compare Figure 9(a,b)).

When the rat began to interact with the closed container trying to retrieve food, locomotor activation accelerated, and brain and muscle temperatures continued to increase at the same rate; these increases were maintained for some time after the container was removed from the cage (Figure 9(c)). While qualitatively similar, these increases as well as changes in NAc-muscle and skin-muscle differentials were significantly greater and more prolonged than those seen in satiated rats, which showed no interest in food (see Figure 9(a)).

Our data demonstrate that food-deprived animals show noticeably different behavioral and temperature responses to a presented food container than non-deprived animals. While satiated rats showed no interest toward this container along with a weak temperature response, hungry rats always initiated food-seeking activity and showed much stronger changes in temperature and locomotion. Therefore, the same “appetitive” stimulus presented to rats in deprived and non-deprived states differentially affects behavior and temperatures, suggesting that specific environmental stimulation is essential to reveal (actualize) motivational state and induce food-seeking behavior. Despite a smaller amplitude and duration, the initial phase of the temperature and locomotor response induced by the container’s presentation was similar in hungry and satiated conditions, mimicking the response induced by other salient somatosensory stimuli. Therefore, the same food container (a combination of visual and olfactory stimuli) when presented in non-deprived conditions could be defined as a stimulus inducing an arousal response. Therefore, increased arousal (or nonspecific activation) could be viewed as both a precondition for and a correlate of specific motivated behavior.

While the initial temperature responses elicited by the food container were similar in all three situations, distinct differences were seen in food-deprived rats depending on food availability. When the container was closed, rats showed sustained food-seeking activity trying to retrieve a food pellet. In this case, NAc and muscle temperature gradually increased in parallel with locomotion, and these increases slowly disappeared after the container was removed from the cage. When the container was open, food-seeking activity rapidly resulted in food consumption that was associated with the second phase of temperature increases that peaked at the time when eating was finished. The rate of temperature increase rapidly grew after the start of eating, was maintained during its entire duration, and sharply dropped when eating was completed. While the NAc-muscle differential increased after the presentation of the container and during food seeking, it began to decrease during eating. While this change suggests rapid cessation of previous metabolic neural activation, the stronger change in temporal muscle temperature could be explained at least in part by heat inflow from *mm. masseter*, which is located in a close proximity to the temporal muscle and is heavily implicated in chewing. Therefore, a strong temperature increase in the muscle and an increase in the skin temperature (due to increased arterial blood inflow to the head) could be viewed as a consequence of specific muscular activity associated with chewing. However, despite this increased motor activity associated with eating, temperature increases were not related to general locomotion, which was drastically reduced while increases in temperature were maximal. On the other hand, temperatures began to fall after cessation of eating when the rat became hyperactive again, engaging in extensive washing, grooming, and exploratory behavior.

Overall, motivated food-seeking behavior is accompanied by modest brain hyperthermia that results from sustained metabolic neural activation and retention of body heat due to peripheral vasoconstriction. This metabolic neural activation is triggered via somato-sensory pathways and maintained during seeking behavior until successful interaction with the reinforcer (reward = start of food consumption) and its detection by the CNS. While brain temperature continues to increase during food consumption and strong peripheral vasoconstriction appears to be the primary contributor to this effect, metabolic neural activation as reflected by brain-muscle temperature differentials gradually decreases. The end of consumption is related to a rapid decrease in brain temperature and slow restoration of temperatures to their baseline. In addition to rapid “eating-stopping” neural signals from the stomach during food consumption, the brain needs to receive metabolic signals from consumed food to induce satiety and stop eating behavior. If the rat is engaged in food-seeking activity but food remains unavailable, brain hyperthermia coincides with metabolic brain activation and strong, prolonged peripheral vasoconstriction. Therefore, it appears that successful interaction with the reinforcer (food for hungry animals) eventually results in cessation of metabolic neural activation and disappearance of food-seeking behavior, correlating with satisfaction (reward).

#### Drinking behavior

As a logical extension of our previous studies on sexual and feeding behavior, thermorecording was used to examine drinking behavior, established by rat’s interaction with another reinforcer – a sweet caloric solution [113]. In contrast to food that is reinforcing only for hungry animals (state-dependent reinforcer), sweet solution is a state-independent reinforcer, and rats rapidly learn to consume sweet drinks without any food or water deprivation. As a sweet substance, we used caffeine-free Coca-Cola Classic® or Coke® (11% sugar content). Before surgeries, rats were pre-trained to consume a 5-ml portion of this drunk presented in a cup and our temperature recordings were conducted in well-trained rats, which consistently consumed Coke solution. Our primary goal was to examine the pattern of central and peripheral temperature fluctuations and locomotor activity associated with different aspects of motivated Coke-drinking behavior and its variants associated with changes in the animals’ motivational state (hunger vs. satiety) and the nature of the reinforcer (water or Diet Coke vs. regular Coke). Based on these data, our general goal was to learn more about basic mechanisms underlying motivated appetitive behavior.

**Figure 10.**
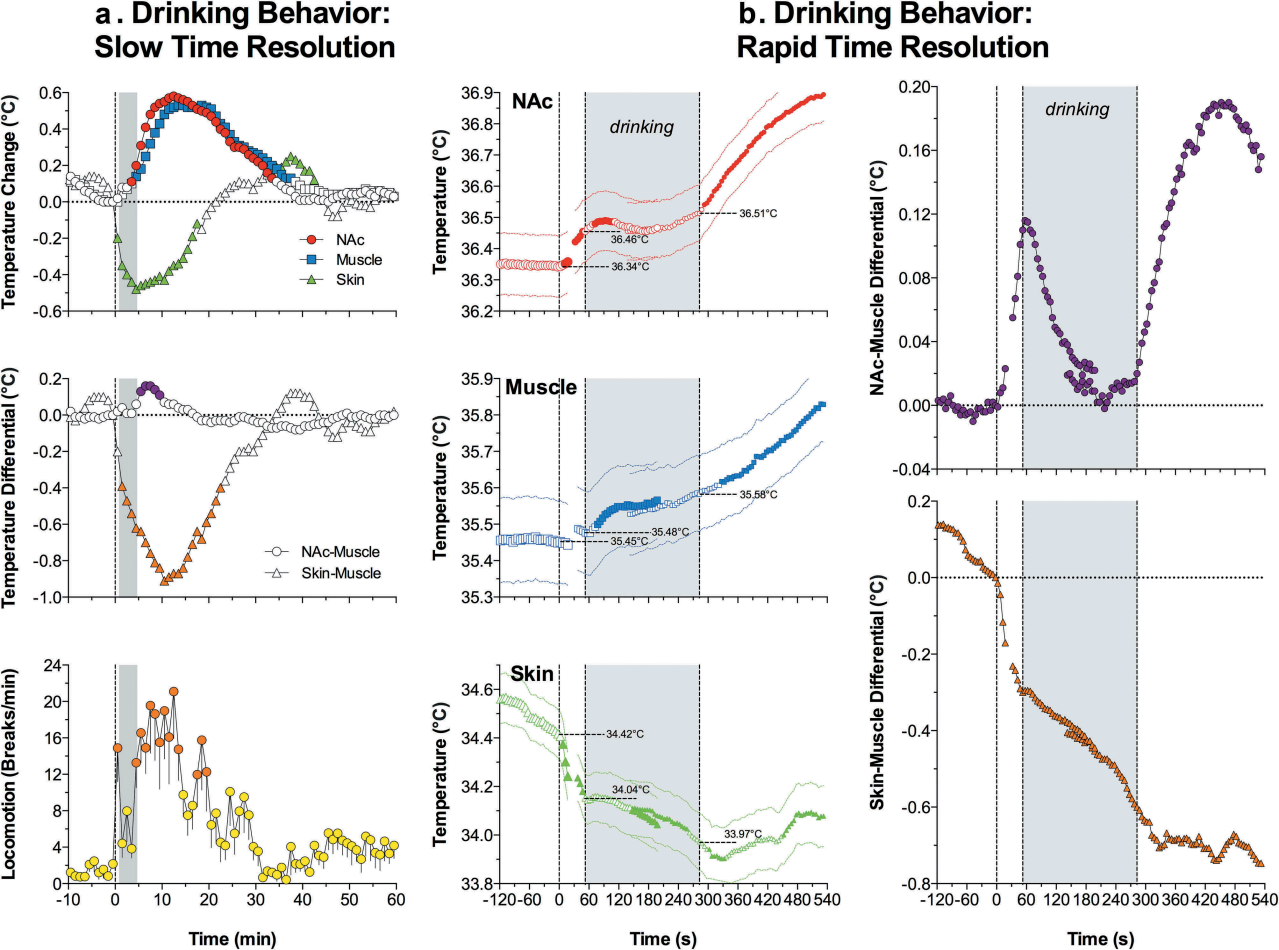
Slow (A) and rapid (B) temperature changes in the brain (NAc), temporal muscle, and skin during drinking of Coke solution. First vertical line in each graph shows the moment of presentation of glucose-containing cup, gray area shows the duration of drinking. Data analyzed with slow (1-min) time resolution shows mean changes in temperature and locomotion from the moment of cup presentation. Data analyzed with high temporal resolution (10-s) show changes with respect to three critical events (cup presentation, start of drinking, and end of drinking). Filled symbols show values significantly different from either baseline (A) or each individual event of drinking behavior. Original data were reported in [113] and replotted for this article.

When analyzed with slow (1-min) time-resolution, free-drinking behavior was associated with a typical triad of temperature changes coupled with increased locomotion (Figure 10(a)). Brain and muscle temperatures began to increase after the Coke-containing cup was presented to the rat and the rat initiated seeking behavior, further increased during drinking and showed the largest increase during the post-drinking period. In contrast, skin temperature rapidly decreased after cup presentation, reached its nadir at the end of drinking, and slowly returned to baseline thereafter. Locomotion rapidly increased after cup presentation, relatively decreased during drinking, and more tonically increased during the post-consumption period.

More complex temperature dynamics were found when data were analyzed with high (5-s) time resolution (Figure 10(b)) with respect to three key events of motivated drinking behavior: cup presentation, initiation of drinking, and its completion. As can be seen, NAc temperature began to increase rapidly following full cup presentation, becoming statistically significant at 10–15 s. This increase continued gradually until the rat initiated drinking, but began to decrease during drinking. In contrast to the NAc, muscle temperature virtually did not change during cup presentation and before the initiation of drinking, slightly increased during drinking, and continued to increase slowly but gradually during the post-drinking behavioral activation. Due to these differences, NAc-muscle differentials rapidly increased during seeking behavior, peaked at the moment when the rat began drinking, and gradually decreased during drinking. Finally, NAc temperatures and NAc-muscle differentials began to increase again after drinking was finished and continued increasing during the post-drinking behavioral activation. In contrast to the NAc and muscle, skin temperature decreased sharply after cup presentation. This rapid decrease weakened during drinking, became stronger again at the end of drinking, but then inverted into a weaker increase from 25 to 30 s after the end of drinking. Due to these differences, skin-muscle differentials decreased rapidly and strongly following cup presentation, continued to decrease at a slower rate during drinking, and remained at relatively stable low levels for some time after the end of drinking.

After establishing the basic pattern of temperature changes associated with motivated drinking behavior, we examined how this pattern would change after substitution of the reinforcer (presentation of Diet-Coke and water). When rats with previous experience of Coke drinking were presented with a cup with an equal amount of *Diet Coke*, a Coke substitute with no caloric value, they first made an attempt to drink it with the same latencies as they did with the Coke in control conditions. While the rats always tasted Diet-Coke and consumed it a little, in no case did rats consumed it entirely. These attempts continued for up to 30–40 min and they were associated with a larger brain temperature elevations and larger increases in locomotor activity than in control conditions with regular Coke. In contrast to relative decreases in NAc and muscle temperatures seen during Coke consumption, this transient effect was absent during Diet-Coke tasting and both temperatures continued to grow rapidly when the rats repeatedly tried to drink it.

Similar changes in temperature dynamics occurred when the rats with previous experience of Coke consumption were presented with an equal amount of *water*. While the rats interacted with the cup with similar latencies and made several attempts to drink water, in no case did they consume it to a significant extent. These attempts continued for up to 20–30 min and were associated with larger temperature increases and stronger locomotor activation. In contrast to temperature decreases during Coke consumption, NAc and muscle temperatures continued to increase rapidly when the rats tasted water, and the increase was stronger than the changes in the control conditions.

Finally, we examined how the pattern of temperature changes associated with Coke consumption would change in hungry rats. After a Coke-containing cup was presented to rats after 24-h food deprivation, hungry rats rapidly initiated drinking, with significantly decreased latencies but for equal duration of time. In contrast to the control conditions, Coke presentation in hungry conditions induced stronger drinking-related decrease sin NAc temperature that resulted in its weaker mean increase compared to the control.

##### Motivated drinking behavior: Appetitive stimulus, arousal, and motivation

Presentation of a Coke-containing cup to an experienced rat results, with a certain variable latency, in initiation of drinking behavior. The same appetitive stimulus also induces locomotor activation and a typical triad of changes in central and peripheral temperatures: increases in brain and muscle temperatures, quicker and stronger temperature acceleration in the brain vs. muscle, and an ultra-fast and profound decrease in skin temperature. However, the same immediate response also occurred when the presented cup contained water or Diet Coke, which resulted in tasting of these beverages with the same range of latencies. Since these changes not only followed a stimulus presentation but also preceded the initiation of drinking (see Figure 10(b)), they could be viewed as a correlate of motivation, a driving force for this seeking behavior. However, as shown above, the same temperature response coupled with locomotor activation occurs in naive rats when exposed to various arousing somatosensory stimuli. Therefore, increased arousal (or a generalized neural activation) could be viewed as both a pre-condition for and a correlate of seeking aspects of motivated behavior (motivated search).

When Coke was substituted with Diet Coke or water, rats tasted the presented drink with the same onset latencies, but did not consume it. While NAc temperatures during these tests showed the same rapid increase until the first drinking attempt, there was no subsequent temperature decrease as had occurred during drinking of regular Coke. In contrast, temperatures continue to increase gradually, peaking at ~15–30 min after cup presentation. Therefore, the inability to obtain expected Coke (or to satisfy existing motivation, i.e. no reward) is associated with sustained neural activation, enhanced intra-brain heat production, and heat accumulation in the brain, manifesting at the behavioral level as a continuous searching activity.

Searching activity coupled with strong increases in NAc temperature and NAc-muscle temperature differentials also occurred after the rats finished Coke consumption and made multiple returns to the empty cup, trying to lick out all remaining drops of liquid. Therefore, the second peak of post-consumption neural activation (which is reflected most clearly in the NAc-muscle differentials) could be related to the reappearance of a motivated search that dissipated slowly over time because of the inability to satisfy this motivation. As shown previously [114], trained rats are able to consume much larger volumes of sugary drinks (up to 30 ml of equally caloric 10% glucose), suggesting that the presented 5-ml drink is unable to satisfy them. Although the post-drinking increases in brain and muscle temperatures are larger in amplitude than those occurring before drinking, this difference results from a variation in time intervals. While trained rats began drinking within a relatively short time interval after the presentation of a Coke-filled cup, hyperactivity and search after emptying the cup continued for much longer (5–15 min), resulting in larger absolute temperature increases despite a slower acceleration (see Figure 10(b)). While the rate of NAc temperature increase immediately after emptying the cup was about the same as before the start of drinking, it weakened gradually within the next several minutes until it inverted into a gradual decrease that slowly returned temperatures from their peaks to their baseline levels. At this stage, animals ceased searching and locomotor activity.

##### Motivated drinking behavior: Consumption and reward

While NAc temperatures gradually increased during searching activity preceding the initiation of Coke drinking, temperature dynamics rapidly changed after drinking began. Although absolute temperature still increased during the first 25–30 s of Coke consumption, the rate of increase dropped within the first 5 s and then inverted into a decrease, resulting in an overall decrease in brain temperatures vs. their values at the start of drinking. Taking into account the link between neural activation and intra-brain heat production, these data suggest that Coke consumption is associated with rapid cessation of preceding neural activation. However, if Coke consumption in experienced rats is viewed as a rewarding event, this rapid change could be viewed as its neural correlate. On the other hand, this change could be viewed as a correlate of the consummatory (satisfying) phase of motivated behavior, which follows its activation-related seeking phase. Despite differences in conceptual interpretation, the surprisingly rapid inversion of NAc temperature dynamics following initiation of drinking suggests that sensory stimulation associated with Coke consumption, not the metabolic consequences of consumed sugars, is the primary factor underlying this change. As shown previously through direct monitoring of brain glucose [115], much more time is needed for sugars to be absorbed from the stomach and affect brain activity. Therefore, this change could also be viewed as a correlate of sensory or “hedonic” but not metabolic reward [116].

#### Common features of temperature dynamics during different forms of motivated behavior

Despite differences in the nature of the reinforcer (food for food-deprived animals, palatable sugary drink, sexual partner) and experimental protocol, there were some common features of brain temperature fluctuations for all three types of examined motivated behavior. In each case, brain temperature rapidly increased in trained rats following the presentation of the reinforcer (food container, Coke-containing cup, sensory signals from receptive female or male) that triggers searching activity. The rate of temperature increases was similar for each reinforcer, but their amplitudes greatly varied for each reinforcer due to differences in experimental paradigms and elicited behavior. Trained rats began drinking within ~1 min after cup presentation and absolute temperature increases were relatively small, but temperatures increased up to 1.5–2.0°C when sexually experienced males were able to see and perceive the receptive female for 20 min, but were unable to interact directly with her. Therefore, an increase in brain temperature with increased brain-muscle differentials, common for all three types of motivated behavior can be viewed as a correlate of searching behavior that is triggered in trained rats by sensory stimuli emitted by the presented reinforcing object. This searching behavior results in direct interaction with the reinforcer – either drinking, eating, or the sequence of mounts and intromissions culminating in ejaculation. In contrast to intact, untrained rats, which showed progressive habituation following repeated exposure to the same testing environment, delayed and weaker habituation was typical of trained animals, which had higher basal brain temperatures and locomotor activity at the start of the behavioral experiment. These changes were seen with each model of motivated behavior but were especially strong with sexual behavior. Therefore, conditioning appears to be another contributor to the dynamics of brain temperature fluctuations during natural motivated behaviors.

Despite certain differences in each model of motivated behavior, brain temperature and brain-muscle differentials began to decrease during interaction with the reinforcing objects, suggesting the cessation of previous metabolic neural activation. This change was rapid and most evident during consumption of a sugary drink (see Figure 10(b)), suggesting that sensory stimulation associated with Coke consumption appears to be a true rewarding event. While this decrease continued until the end of drinking, it rapidly inverted into an increase when the cup became empty and the rat reinitiated searching behavior. During sexual behavior, brain temperature was gradually increased by sexually arousing stimuli and further increased during repeated mounts and intromissions, sharply peaking at ejaculation. After this event, the male’s motor activity decreased rapidly and brain temperatures dropped sharply, with a profound decrease in brain-muscle differentials. In contrast to Coke-drinking behavior, where sensory stimulation associated with the drinking of a sugary substance appears to be a rewarding event that rapidly changes brain temperature dynamics, neural activation triggered by sexually relevant stimuli persists during the entire copulatory behavior, with ejaculation being an acute rewarding event or a “true” satisfier of this motivated behavior. Importantly, the same biphasic changes in brain temperature and brain-muscle differentials also occurred in female rats. However, peak of neural activation in females occurred within a minute after the rat received the male’s ejaculate and this appears to be a true rewarding event for the female. Although brain temperature increased during food consumption, this increase was also coupled with a gradual decrease in brain-muscle differentials, suggesting consumption *per se* as also being a true rewarding event for feeding behavior. Importantly, this reward-related change was absent when the food-container was closed and the rat was unable to retrieve the food. Therefore, it appears that different motivated behaviors, despite different manifestations, share some common basic patterns of alterations in metabolic brain activity associated with critical behavioral events.

## Brain hypothermia associated with general anesthesia: Underlying mechanisms

**Figure 11.**
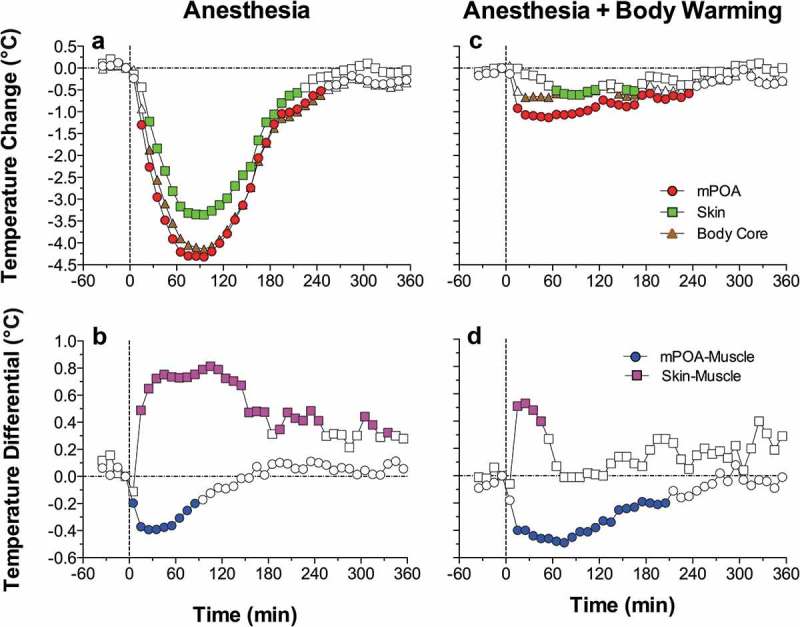
Mean changes in brain (mPOA), skin, and body core temperature and temperature differentials during general anesthesia induced by sodium pentobarbital without and with body warming. Vertical hatched line = the moment of ip drug injection. Filled symbols show values significantly different from baseline (ANOVA followed by Fisher test p < 0.05). Data were replotted from reference [119].

It is well known that general anesthesia is associated with a robust decrease in body temperature and impairment of temperature regulation, making an organism more sensitive to external temperature influences [117,118]. To counteract this impairment and prevent hypothermia, anesthesia is usually supplemented by body warming, thus opposing the temperature-depressing effects of general anesthetics and helping to maintain temperatures within their physiological limits. We found that general anesthesia induced by sodium pentobarbital (50 mg/kg, ip) administered to awake, quietly resting rats in standard ambient temperatures (22°C) causes robust brain hypothermia (>4°C below baseline) as assessed in the mPOA [119] (Figure 11(a)). Temperatures in the body core and skin also decreased, but temperature decreases in the brain were stronger than in the body core, resulting in a significant drop in mPOA-body temperature differentials (Figure 11(b)) – indicative of brain metabolic inhibition. The temperature decrease in the skin was weaker than in the body core, resulting in a robust rise in skin-body differentials (Figure 11(b)), indicating skin vasodilation. Therefore, during general anesthesia, the brain becomes relatively cooler and skin relatively warmer than the body core. While the observed brain temperature decreases reflect the known inhibiting effect of pentobarbital on brain metabolism and intra-brain heat production [120,121], skin temperature increases indicate peripheral vasodilatation that enhances heat loss to the external environment. Therefore, two basic mechanisms–diminished intra-cerebral heat production and increased heat loss to the cooler external environment–are responsible for brain hypothermia induced by pentobarbital.

We also examined how the basic pattern of central and peripheral temperature responses induced by pentobarbital changes when the drug is administered to rats that have their bodies passively warmed (Figure 11(c)). In this case, decreases in brain, body core and skin temperature were much smaller, but passive body warming was unable to reverse the decreases in brain-body core temperature differentials, which were stronger and more prolonged than in conditions with no body warming (Figure 11(d)). Although body warming effectively counteracts heat loss to the external environment and attenuates pentobarbital-induced brain hypothermia, the brain remains metabolically inhibited under these conditions and its temperature remains relatively lower than in the body core.

Selective brain cooling during general anesthesia could explain the differences in brain and body temperatures reported in different studies. While in awake monkeys and human patients brain temperatures in different locations are similar or slightly higher than body temperature [30,122–124], these relationships become inverted during anesthesia. In cats under halothane and pentobarbital anesthesia with body warming cortical tissue was 1.0 and 1.8°C colder, respectively, than the body core [68]. Similar negative brain-body differentials were found during pentobarbital anesthesia in dogs [125], urethane anesthesia in rats [67], and anesthesia induced by alpha-chloralose and chloral hydrate anesthesia in rats [126]. During urethane anesthesia without body warming, the temperature difference between the Hippo and rectum was about 4.2°C [67], and during chloralose anesthesia with body warming the difference between the cortex and body core reached 4.3°C [126]. In contrast to our study, which employed chronically implanted temperature sensors, these evaluations were assessed in experiments utilizing acutely implanted metal electrodes, which were not properly thermo-insulated and often with an open skull. These experimental conditions result in undervalued brain temperatures, and the measurement mistake could be especially large in smaller animals (i.e. mice and rats) and for superficial recording sites.

As shown above, the positive brain-arterial blood temperature difference assessed in animals is maintained under different conditions and becomes larger (because of metabolic brain activation) following exposure to various environmental challenges [36]. Temperature of arterial blood is also consistently lower than that in brain venous outflow in human volunteers tested under various conditions, including vigorous exercise in a warm, humid environment [86]. While these data suggest that heat generated in the periphery cannot be delivered to the brain under physiological conditions, the direction of heat exchange between the brain and rest of the body can be altered during general anesthesia, especially when it is combined with passive body warming. Under these conditions, temperatures in brain sites decrease to a greater extent than in the body core due to metabolic brain inhibition, while body core and arterial blood temperatures become warmer than those in brain tissue due to external body warming. As a result of these two opposing influences, during general anesthesia, the metabolically inhibited brain remains cooler than the externally warmed body, and arterial blood becomes the primary means of brain warming.

**Figure 12.**
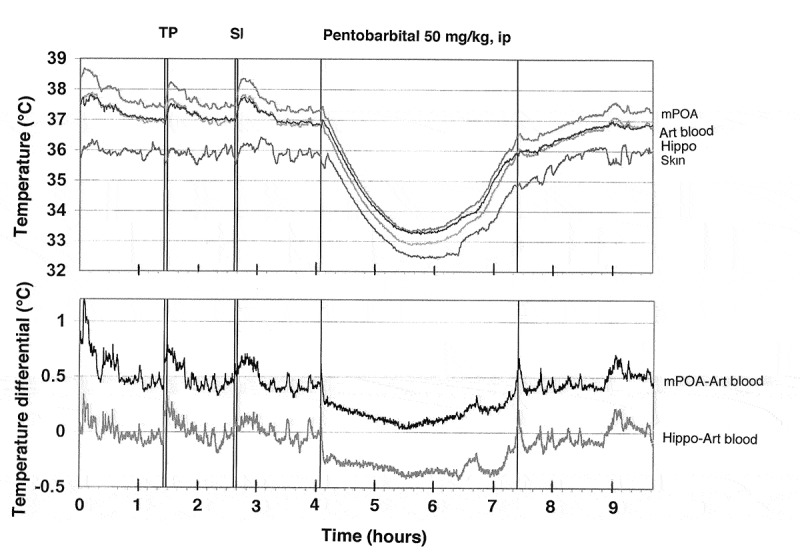
Original record of changes in temperature in the brain (mPOA, Hippo), skin, and arterial blood during arousing stimulation (TP, tail-pinch and SI, social interaction) and general anesthesia induced by sodium pentobarbital. Data were obtained in 2007 and previously not published.

To clarify this point, we conducted a similar experiment with pentobarbital, but instead of the body core, we monitored arterial blood temperature by using a miniature sensor chronically implanted into the abdominal aorta via caudal artery. As can be seen in Figure 12, mPOA temperature before the injection of pentobarbital (50 mg/kg, ip) was ~0.5°C higher than that in arterial blood. Consistent with our previous work, this difference phasically increased during tail-pinch and social interaction but strongly decreased during anesthesia. However, even at the lowest point, this difference remained positive. In contrast, the Hippo-arterial blood difference was minimal before pentobarbital injection and became negative during anesthesia. Therefore, we cannot exclude that under these physiologically unusual conditions, heat could be transferred to the brain from the periphery.

Therefore, in addition to the direct action of anesthetic drugs on neural cells and a dramatic change in afferent inputs during general anesthesia, brain hypothermia can significantly confound the results of neuronal and neurochemical studies conducted in anesthetized animal preparations. While body warming during general anesthesia could effectively compensate for drug-induced body hypothermia, it is unable to fully compensate for brain hypothermia. Furthermore, external body warming during anesthesia may invert the normal direction of heat exchange between the brain and arterial blood, thus affecting various metabolic and blood flow parameters. For this reason, work conducted in such preparations as well as in brain slices maintained at room temperatures (23°C) can produce an obscured, distorted view of the active brain.

## Brain temperature as a factor affecting neural activity, brain structure, and functions

Physiological changes in brain temperature can be considered from two different standpoints. From one standpoint, temperature changes can be viewed as transient alterations of brain temperature homeostasis that are triggered by neural activation and result from intra-brain heat production and decreased heat dissipation from brain tissue. According to this view, brain temperature is an important physiological parameter, which can provide information on changes in brain metabolic activity occurring during different motivated behaviors. However, according to another standpoint, changes in brain temperature can be viewed as a factor that directly affects different forms of neuronal activity, multiple neurophysiological and neurochemical evaluations, and structural integrity of brain cells. It is well known that most physical and chemical processes governing neural activity are temperature-dependent, with the averaged Van’t Hoff coefficient Q_10_ = 2.3 (i.e. doubling with a 10°C change [127]. However, *in vitro* studies revealed that temperature dependence greatly varies depending on the specific neural parameters, the type of cells being studied, and the nature of afferent inputs involved in mediating neural responses [128–131]. These studies also confirmed the classic view that synaptic transmission is more temperature-dependent than the generation of action potentials [132].

Classic views on regulation of body temperature consider temperature-sensitive neurons located in the preoptic/anterior hypothalamus as primary central temperature sensors [133–135]. However, cells in many other structures also exhibit a high degree of temperature sensitivity, which is often similar or greater than that in hypothalamic neurons. For example, 22% of medial thalamic neurons have a positive thermal coefficient >0.8 imp/s/°C [136], exceeding the number of temperature-sensitive cells in the anterior (8%) and posterior (11.5%) hypothalamus. In the superchiasmatic nucleus, ~18% of neurons are warm-sensitive [137], while >70% decrease their activity rate with cooling below physiological baseline (37–25°C) [138]. Finally, high temperature sensitivity was found in electrophysiologically identified substantia nigra DA neurons *in vitro* [139]. Within the physiological range (35–39°C), their discharge rate increases with warming (Q_10_ = 3.7) and dramatically decreases (Q_10_ = 8.5) with cooling below this range (34–29°C). This latter finding questions the validity of basal electrophysiological properties and responses of DA neurons studied *in vitro* at low, non-physiological temperatures. When studied in awake, freely moving conditions, this neuronal population is much more heterogeneous with multiple subgroups that are not apparent in assessments made in brain slices and anesthetized animal preparations [73].

The high temperature dependence of various neural parameters, ranging from the activity of single ionic channels to transmitter release and uptake has important implications. Although *in vitro* experiments permit individual cells to be studied and individual components of neural activity and synaptic transmission to be separated, neural cells in the living brain are interconnected and their integral changes should be different from those of individual components assessed *in vitro*. For example, temperature-dependent increases in transmitter uptake should compensate for temperature-dependent increases in transmitter release. By increasing both release and uptake, however, brain hyperthermia makes neurotransmission more efficient (but at higher energetic cost) and neural functions more effective at reaching behavioral goals. Therefore, changes in temperature may play an important integrative role, involving and uniting numerous central neurons within the brain.

However, temperature increases or decreases exceeding physiological range (35–39°C) can adversely affect brain cells and neural functions. While brain cells seem to tolerate low temperatures well [140,141], multiple *in vitro* studies suggest that high temperature (>40.0°C) has destructive effects on various types of brain cells [128,142–147]. Rapid damage to brain cells has been also documented during acute METH intoxication [148,149], which induces robust brain hyperthermia [150]. METH intoxication has also been associated with increased permeability of the BBB, resulting in vasogenic edema [148,151], which can often result in animal death. The integrity of the BBB has also been shown to be compromised during opiate withdrawal [152], intense physical exercise in a warm environment [153], and during restraint and forced-swim stress [154–156] – conditions associated with brain hyperthermia [86,157].

To clarify the role of brain temperature in modulating BBB permeability and subsequent changes in neural parameters, it is essential to delineate this physical factor from other possible contributors. To achieve this goal, we examined several brain parameters in pentobarbital-anesthetized rats, whose bodies were passively warmed to produce different levels of brain temperature, which was monitored by chronically implanted thermocouple probes. As shown above, sodium pentobarbital administered at a typical anesthetic dose (50 mg/kg, ip) under standard ambient temperatures (23°C) and without body warming induces robust brain and body hypothermia (~31–33°C). Due to the latter effect, anesthetized rats became very sensitive to environmental temperatures, becoming hypothermic at low ambient temperatures and hyperthermic when their bodies were warmed. Therefore, through changing the intensity of body warming, we were able to induce a wide range of brain temperatures (measured in the NAc) from very low, hypothermic (>32°C) to very high, hyperthermic (<42°C). This paradigm was used to evaluate the role of brain temperature as a factor affecting BBB permeability, brain tissue water balance, and development of acute morphological abnormalities of brain cells [158]. The brains were taken at the same time after the start of anesthesia (90 min) and the initiation of body warming, but at different levels of brain temperature (32–42.5°C) and analyzed for several brain parameters.

To evaluate the integrity of the BBB we utilized immunohistochemistry for endogenous albumin, a relatively large plasma protein that is normally confined to the luminal side of the endothelial cells and is not present in the brain tissue. Thus, the appearance of albumin-positive cells and albumin immunoreactivity in the neuropil indicates a breakdown of the BBB. We also examined expression of glial fibrillary acidic protein (GFAP), an index of astrocytic activation [152,159], as well as the changes in tissue water content and the presence of morphologically abnormal cells as assessed through traditional staining techniques.

**Figure 13.**
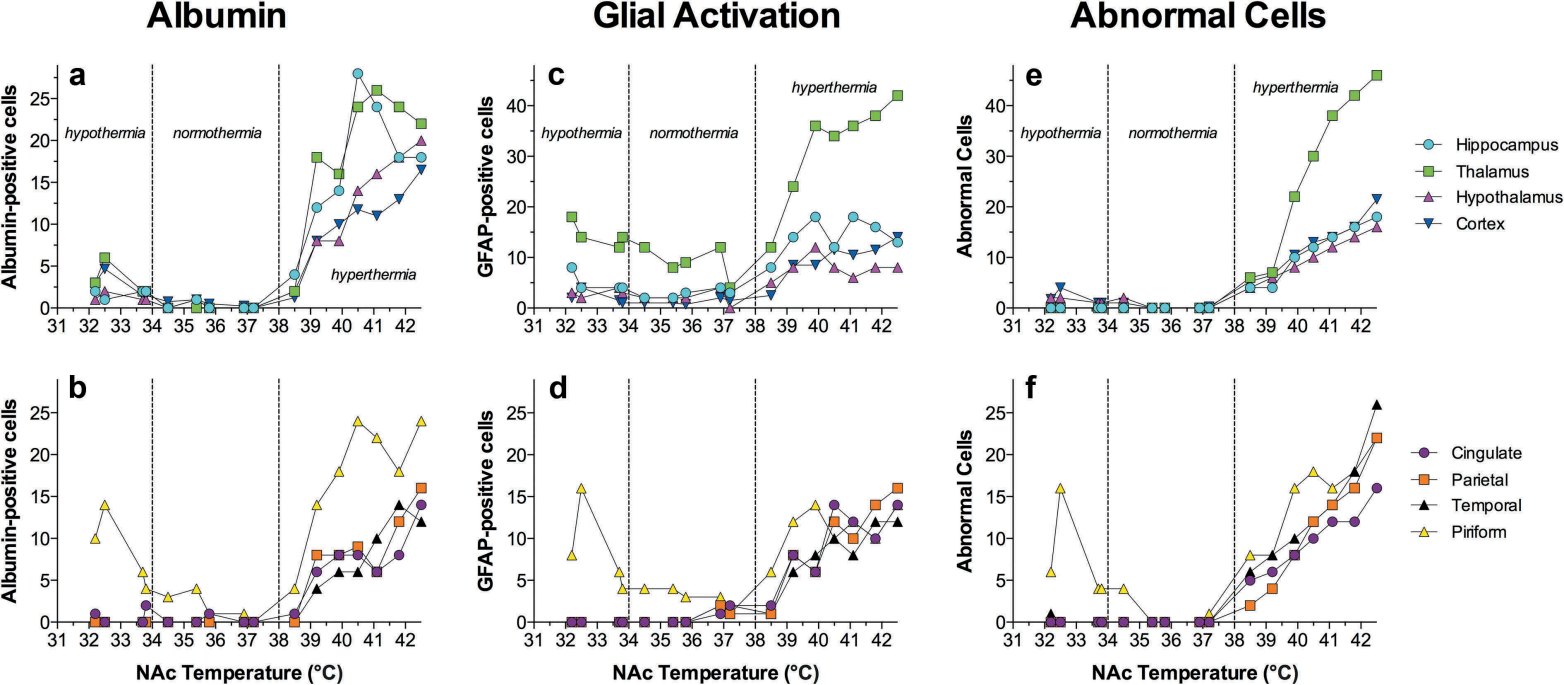
Temperature dependence of albumin immunoreactivity (Albumin), GFAP immunoreactivity (Glial Activation), and cellular brain abnormalities (Abnormal Cells) in pentobarbital-anesthetized rats passively warmed to different temperatures. Data on top graphs show values for four brain structures and data on bottom graphs show values for individual cortical areas. Values on abscissa show values of NAc temperature at which brain samples were taken. Data were replotted from reference [158].

As shown in Figure 13(a), the number of albumin-positive cells assessed in four brain structures (hippocampus, thalamus, hypothalamus, cortex) is strongly dependent on brain temperature, being minimal at normothermic values (34.2–38.0°C), slightly higher (2-4-fold) at hypothermic values (34.2–32.2°C), and dramatically higher (~26-fold) at hyperthermic values (38.0–42.5°C). The increase was evident from ~39°C, progressed at higher temperatures, and plateaued at high levels at 41–42°C. Despite a similar trend in each structure, there were some structural differences in albumin immunoreactivity, with maximal values in the thalamus and hippocampus. Certain differences in albumin immunoreactivity were also found in different cortical areas, with the strongest changes in the piriform cortex – a ventrally located cortical area with the highest temperature (Figure 13(b)). In this structure, the number of albumin-positive cells at low brain temperatures was clearly higher than those at a normal temperature range.

The number of GFAP-positive cells, indicative of acute glial activation, was also directly dependent on brain temperature (Figure 13(c,d)), but changes were less pronounced than those for albumin. Similarly, the largest number of GFAP-positive cells was found in the thalamus. Only sporadic GFAP-positive cells were found in most cortical areas at low and normal temperatures, but their numbers grew at higher temperatures. Similar to albumin, the largest number of GFAP-immunoreactive cells was found in the piriform cortex and these numbers were higher at low vs. normal temperatures. Finally, we assessed the relationship between brain temperature and the incidence of structurally abnormal cells (Figure 13(e, f)). In this case, only a few abnormal cells were found in all brain structures at low and physiological temperatures, but their numbers were clearly larger at high temperatures. Among the structures tested, the largest change was found in the thalamus, and the piriform cortex clearly differed from other cortical areas, showing an unusually high number of abnormal cells at very low temperatures.

**Figure 14.**
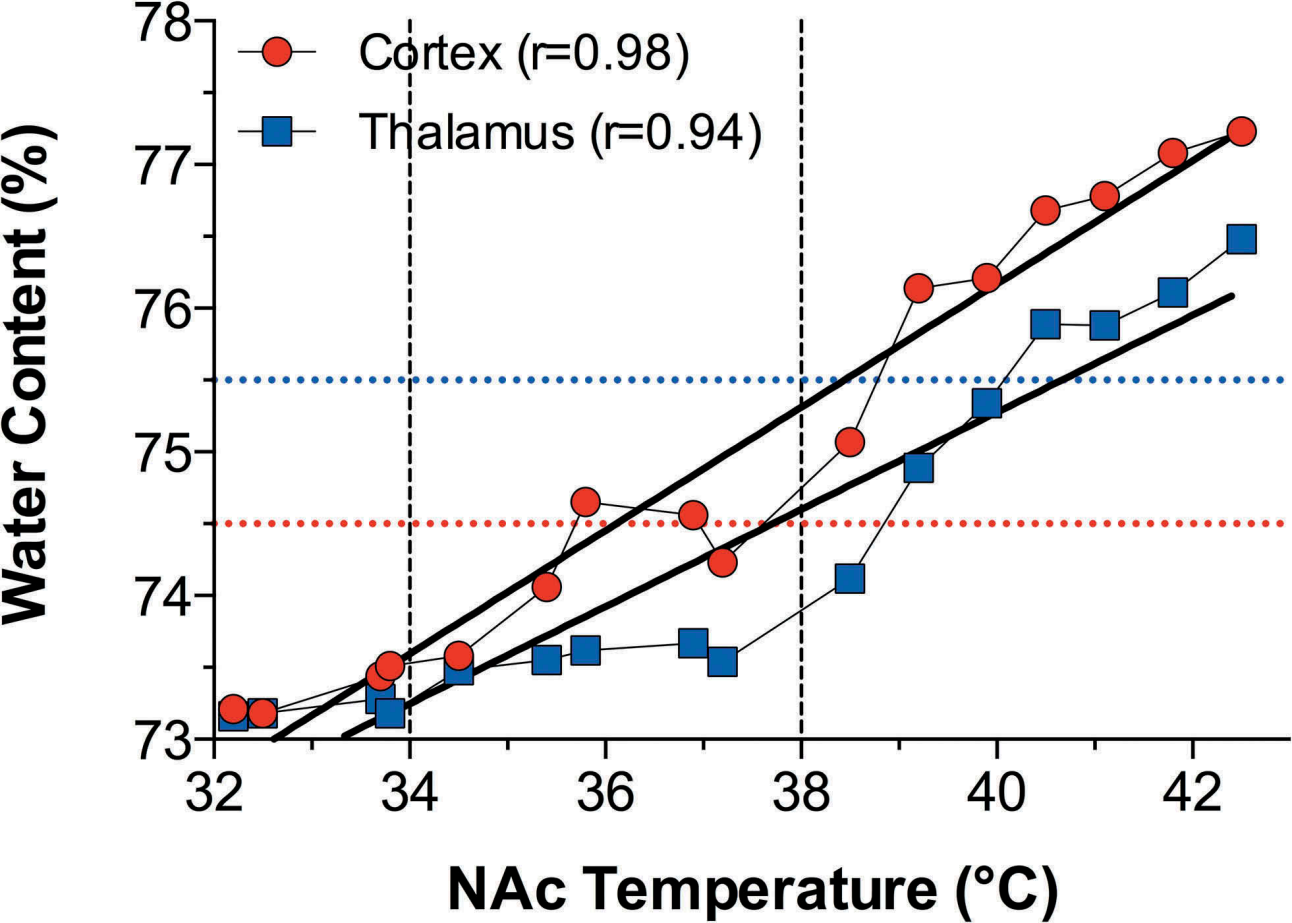
Temperature dependence of tissue water in the cortex and medial thalamus in pentobarbital-anesthetized rats passively warmed to different temperatures. Horizontal hatched lines show “normal” values evaluated in brains of awake, drug-free rats at normothermia. In each structure water tissue content tightly and linearly correlates with brain temperature (p < 0.001). Data were re-plotted from reference [158].

It is well established that leakage of the BBB results in robust alterations in brain ionic balance and increased water accumulation in brain tissue [160,161]. As shown in this study, tissue water content (evaluated in the cortex and thalamus) was also strongly dependent on brain temperature (Figure 14). Water content in cortical tissue was lowest during hypothermia, significantly higher during normothermia, and maximal during hyperthermia. Within the range of recorded temperatures, cortical water differed within ~4%. In the thalamus, water content was highest during hyperthermia, but values at low and normal temperatures were virtually identical. Cortical water content in anesthetized rats passively warmed to “normal” temperatures was similar to that in awake control conditions (see blue and red dotted lines), but significantly higher (edema) in rats warmed to hyperthermia and significantly lower (dehydration) in those with very low temperatures. A similar trend was found in the thalamus, where water content during anesthesia was lower than in awake controls at low and moderate brain temperatures and similar to control at high temperatures. Considering the entire range of brain temperature values, water content was directly and strongly dependent on brain temperature in both the thalamus and cortex; the correlation was equally strong in both locations.

**Figure 15.**
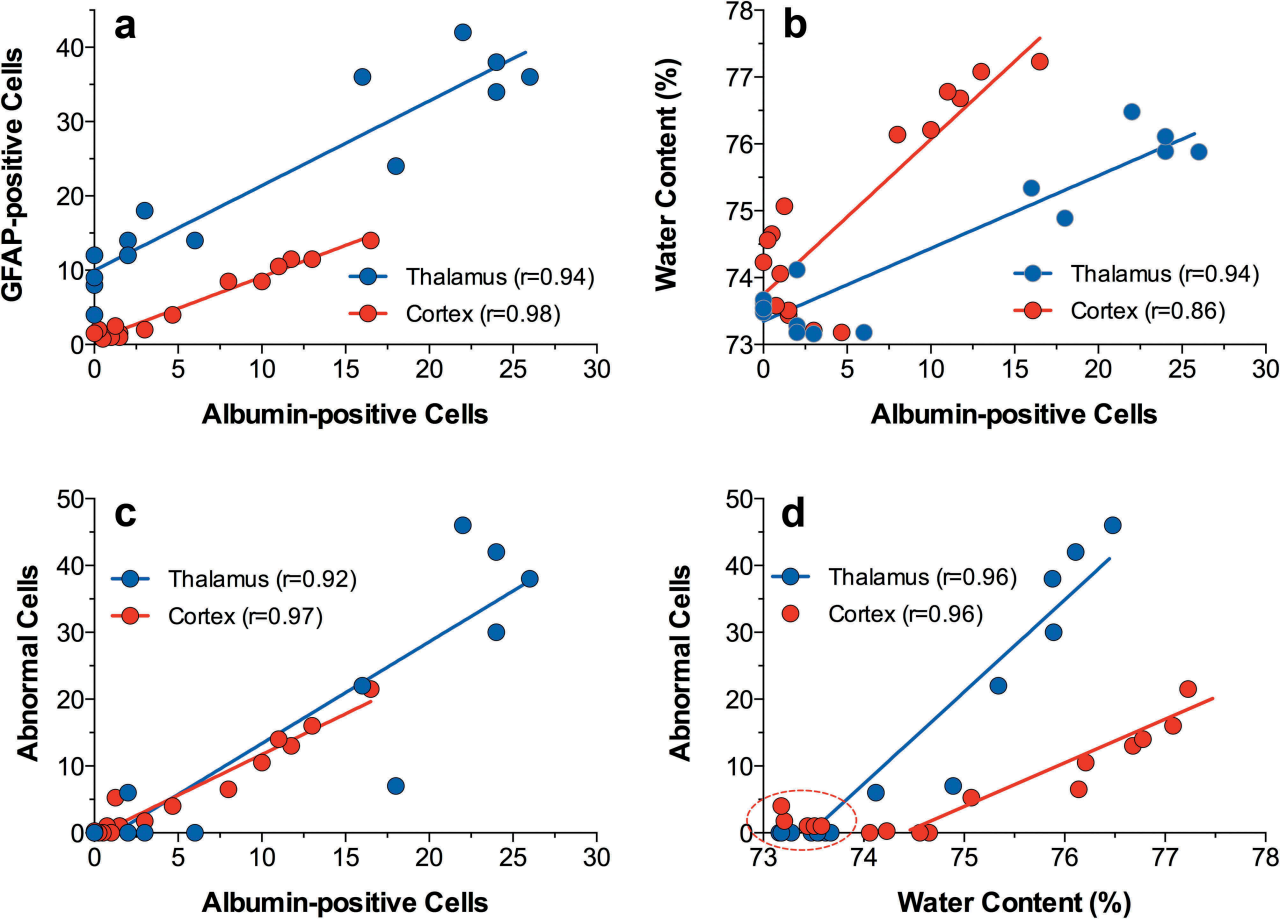
Correlative relationships between individual brain parameters in pentobarbital-anesthetized rats passively warmed to different temperatures. A = relationships between the counts of albumin- and GFAP-positive cells evaluated in the thalamus and cortex; B = relationships between the counts of albumin-positive cells and tissue water content; C = relationships between the counts of albumin-positive cells and structurally abnormal cells; D = relationships between tissue water content and the counts of albumin-positive cells. Each correlation coefficient value is highly significant (p < 0.001). See the text for other explanations. Original data were published in reference [158] and re-plotted for this article.

After assessing the temperature dependence of several neural parameters, we examined their relationships in two brain structures: the thalamus and cortex (Figure 15). As can be seen, the number of albumin-positive cells directly correlated with the number of GFAP-positive cells, suggesting tight relationships between temperature-dependent leakage of the BBB and acute glial activation (Figure 15(a)). However, the change was quantitatively larger for GFAP-positive cells, as their numbers were higher at low temperatures when only a few albumin-positive cells were found. The numbers of albumin-positive cells and tissue water content were also closely interrelated in both structures (Figure 15(b)), suggesting that leakage of the BBB is associated with water accumulation in brain tissue. This correlation was almost linear in the thalamus, but some divergence was found in the cortex at temperatures that correspond to extreme hypothermia. Despite the presence of a few albumin-positive cells, cortical water was relatively low, suggesting that the tight correlation between brain albumin and water, which exists within the entire range of normal and high temperatures, could be distorted at very low temperatures. Therefore, it appears that not only extreme hyperthermia but also extreme hypothermia result in BBB leakage, but “hypothermic” brains appear to be dehydrated compared to “normothermic” brains taken from both awake and anesthetized animals at normal temperatures. It is unclear, however, whether these disturbances in water homeostasis are related to hypothermia *per se* or to pentobarbital’s inhibiting effects on metabolism [120,162]. As shown above, temperature in deep brain structures never drops below 34–35°C under any physiological conditions and such extreme hypothermia can only be seen during general anesthesia, near-lethal environmental temperature drops, or overdose with powerful sedative drugs.

Strong temperature dependence was also found with respect to leakage of the BBB and the appearance of morphologically abnormal cells (Figure 15(c)). In both the cortex and thalamus, structural cell abnormalities were absent at low and normal temperature, but their numbers linearly increased during hyperthermia. Finally, the numbers of abnormal cells in both structures closely correlated with tissue water content suggesting a link between the development of vasogenic edema and structural abnormalities of brain cells (Figure 15(d)). Abnormal cells were absent when tissue water content was low (see a circle in Figure 15(d)), but their number grew exponentially when water content was high.

These data indicate that robust brain hyperthermia is a strong factor eliciting breakdown of the BBB, with subsequent changes in multiple brain parameters. Importantly, albumin immunoreactivity appeared in brain tissue relatively quickly (20–80 min) and within the range of physiological hyperthermia (38.5–39.5°C), suggesting that increased BBB permeability is not solely pathological but also a normal physiological phenomenon transiently occurring and disappearing during various conditions associated with hyperthermia. While the number of albumin-positive cells plateaued at high temperatures, morphological abnormalities linearly increased and peaked at the maximum detected temperature (42.4°C), thus confirming multiple *in vitro* observations suggesting that brain cells are exceptionally sensitive to thermal damage. Similar to other parameters, the structural abnormalities of brain cells occurred relatively quickly and were closely related to BBB leakage and increased tissue water content. Therefore, even with passive warming, morphological damage reflects not only the effect of temperature *per se*, but also BBB leakage and associated edema.

**Figure 16.**
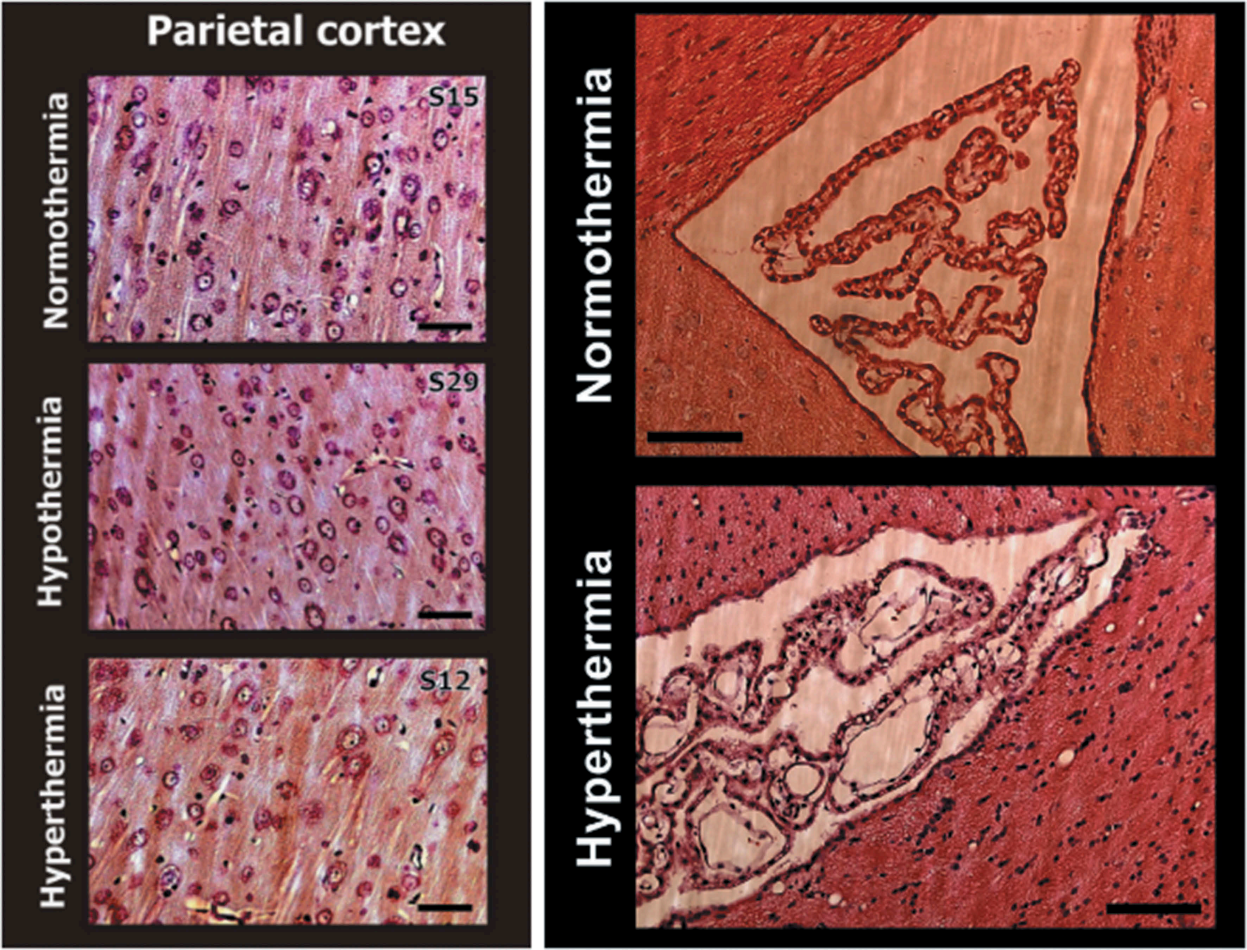
Temperature dependence of structural brain changes. Left pictures show Nissl-stained sections from similar areas of the deep parietal cortex obtained from rats maintained at different levels of NAc temperature (S15 = 36.05°C; S29 = 32.30°C; S12 = 41.80°C). Brain cells during hyperthermia have larger somata and wider axons (arrows) compared to those in normothermia. In contrast, during hypothermia cells are slightly smaller in size, staining is more condensed, and axons are smaller in diameter compared to normothermic conditions (arrows). Bar for each graph is 100 μm. Right pictures show profound changes in structural integrity of the choroid plexus during extreme hyperthermia. All slices were made over lateral ventricles, stained with Hematoxylin-Eosin, and images are shown with equal magnification (bar = 200 μm). In contrast to the healthy structure in normothermic conditions, robust disintegration of epithelial cells and profound vacuolization were typical of hyperthermic conditions. Modified from reference [158].

Several examples of temperature-dependent structural changes in brain cells are shown in Figure 16. The left panel shows Nissl-stained sections from similar areas of the parietal cortex obtained from rats at different levels of brain temperature. As can be seen, brain cells during extreme hyperthermia (S12 = 41.80°C) have large somata and wider axons compared to those in normothermic conditions (S15 = 36.05°C). In contrast, during hypothermia (S29 = 32.30°C) cells are slightly smaller in size, staining is more condensed, and axons are smaller in diameter compared to normothermic conditions. Examples in the right panel show profound changes in structural integrity of the choroid plexus, a structure located inside of brain ventricles which produces cerebrospinal fluid, during extreme hyperthermia. In contrast to the healthy structure in normothermic conditions, robust disintegration of ependymal cells (a subtype of epithelial cells) and profound vacuolization were typical of hyperthermic conditions. In contrast to compact and dense ependymal cells with distinct nuclei in normothermic and hypothermic conditions, marked degeneration with no distinction between individual cells, dense nuclear staining and vacuolization were seen in brain slices obtained at extreme hyperthermia. Robust but possibly reversible structural changes of the choroid plexus could be related to high metabolic activity of ependymal cells, which abundantly express water channels [163,164] and are involved in high energy-consuming active transport of various substances into and out of the cerebrospinal fluid [165]. Robust morphological abnormalities found in this structure could explain, at least in part, between-structural differences in albumin and GFAP immunoreactivity, water content, and structural changes, especially in regions proximal to the ventricles.

## Brain temperature as a factor affecting neurochemical evaluations in behavioral experiments

Functioning of the CNS depends on dynamic changes in multiple neuroactive substances in localized areas of the brain. The recent progress in understanding brain neurochemistry has been achieved primarily by using *in vivo* microdialysis and voltammetry – two neurochemical techniques, which allow the detection of changes in different endogenous substances in the brain’s extracellular space in awake, freely moving animals. Microdialysis is widely used to study changes in extracellular levels of different substances, but it is essentially slow, placing significant constraints for revealing rapid fluctuations in these substances. In contrast, electrochemical techniques have excellent temporal and better spatial resolution, but, as with any indirect technique, selectivity of electrochemical measurements remains a traditional troublesome problem. While electrochemistry is usually used for assessment of electroactive substances that are oxidized by an applied potential [166–169], many important brain substances are not electroactive, requiring alternative strategies for their detection in brain tissue. One strategy is the use of enzyme-based biosensors, which convert the non-oxidizable substance of interest into an oxidizable product that is subsequently quantified based on changes in oxidation current [170–173]. While this approach is based on the premise that changes in detected oxidation currents are overwhelmingly due to changes in extracellular levels of the measured compound of interest, naturally occurring changes in brain temperature (if they are not acknowledged or controlled for via additional tests) could significantly affect measurement precision and sometimes make them unreliable. This issue was important in our recent electrochemical studies on physiological and drug-induced changes in brain glutamate (GLU) and glucose [174–179].

### Electrochemical evaluations of dynamic changes in brain glutamate and glucose in freely moving rats: “unexpected” temperature influences

**Figure 17.**
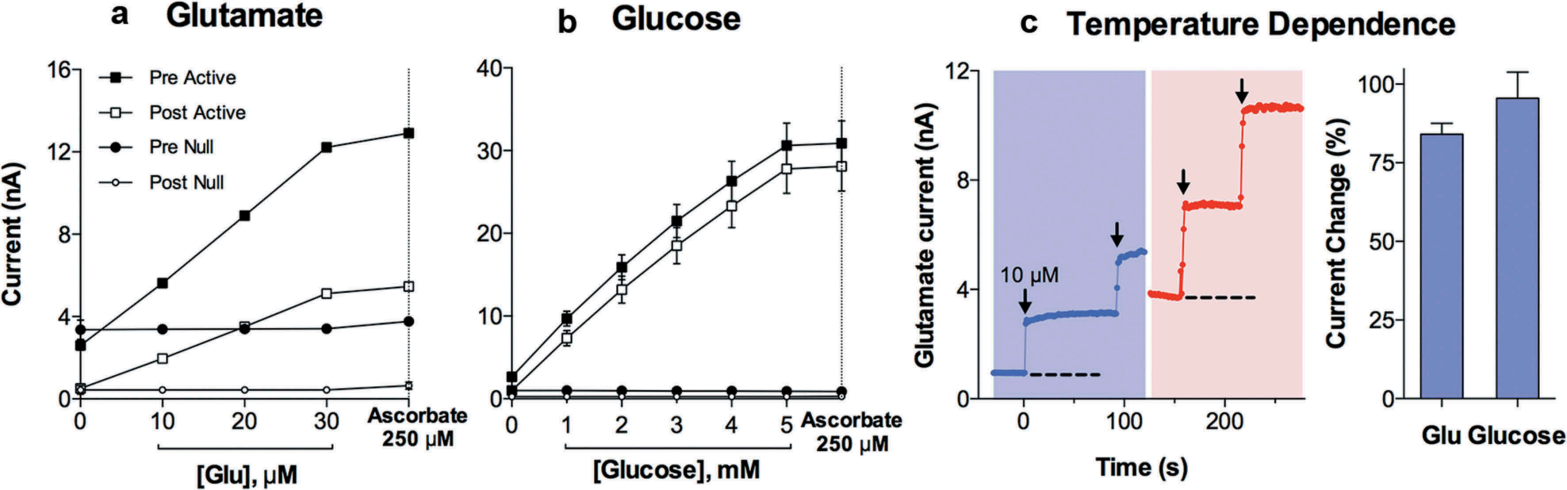
In vitro current responses of GLU- (A) and glucose- (B) selective (Active) and substrate-null (Null) biosensors to different concentrations of substrates before (Pre) and after (Post) an *in vivo* recording. (C) Current response of an example GLU biosensor at 20°C versus 37°C. Dashed lines represent the baseline current *in vitro* at each temperature, each arrow indicates the time when 10 µM GLU was added. Note that the baseline at 37°C is approximately equivalent to the current response to 10 µM GLU at 20°C. Right bar graph indicates the percent increase in current between 20°C and 37°C for the GLU and glucose sensors tested. Original data were published in [174,177]; the picture was reproduced in [187] (ACS Chemical Neuroscience; open access publication).

GLU is a major excitatory neurotransmitter essential for maintaining and regulating central activational processes and glucose is the primary source of energy for the active brain [6,180]. Since GLU and glucose are not natively oxidizable, enzyme-based biosensors overcome this problem through chemically conversing of each non-oxidizable compound into a product plus H_2_O_2_, which can be detected amperometrically as an oxidation current. Therefore, the change in oxidation current detected by these sensors can be viewed as a measure of change in extracellular levels of GLU and glucose. Tested in vitro, GLU and glucose biosensors used in our studies were highly sensitive to these substances and showed high selectivity against other possible chemical interferents (i.e. other readily oxidizable neurochemicals that can evoke current changes such as ascorbate, DA, 3,4-dihydroxyphenylacetic acid or DOPAC, and urate; see [174,175] (Figure 17(a,b)). The sensitivity values of these sensors are sufficient to detect physiological levels of extracellular GLU and glucose, which, based on microdialysis estimates, vary within 0.5–3.0 μM [181–183] and 0.5–2.0 mM [37,184–186], respectively.

Although enzyme-based GLU and glucose biosensors were highly selective with respect to most chemical interferents, they were also temperature sensitive. By comparing temperature sensitivity assessed at standard ambient temperature (22–23°C) and 37°C, the average brain temperature, we found that the sensitivity of both GLU and glucose biosensors determined at 37°C is about two-fold larger than that determined at room temperature. For GLU sensors, the current response grew 84.1 ± 3.5% with a 14°C temperature increase and for glucose sensors grew 95.6 ± 8.3% for a 15°C temperature increase (see Figure 17(c)). Therefore, substrate sensitivity of both types of sensors is temperature-dependent, growing by ~6.2% per 1°C. While these differences could be viewed as small, they result in an almost two-fold overvalue in basal concentrations and concentration changes in both substances during *in vivo* experiments. Therefore, the calculation of concentrations from raw current data in our studies was conducted with a correction of sensitivity values by these temperature coefficients, increasing sensor sensitivity to both substances almost two-fold.

Basal electrochemical currents generated by GLU and glucose biosensors were also strongly temperature-dependent. To quantify this dependence, we monitored background electrochemical currents *in vitro* following slow increasing and subsequent decreasing of temperature in the testing solution within 3–5°C (33–38°C). During these tests, we found that basal electrochemical currents from both types of sensors change ~0.091 ± 0.009 nA with each 0.5°C temperature increase. Therefore, if brain temperature during *in vivo* recording increases 0.5°C, electrochemical currents detected by the sensors could increase up to 0.09 nA, creating an artifactual rise in concentration, which for GLU could reach ~152 nM and for glucose ~6.5 μM. By correlating these values with reported basal levels of extracellular GLU and glucose (~1 μM and ~1 mM, respectively), it is evident that the temperature contribution during *in vivo* recording should be much stronger for GLU (up to 15% of basal levels for 1°C increase) than for glucose (0.5–1.0%).

**Figure 18.**
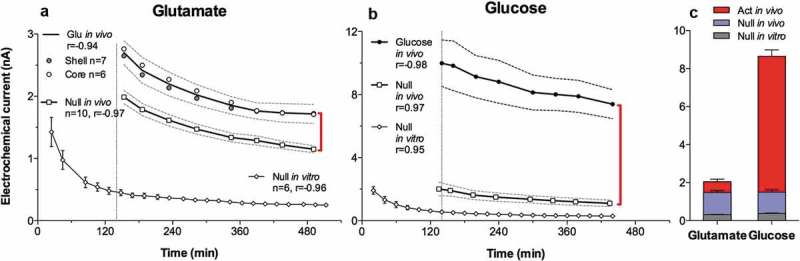
Slow changes (mean±sem) in electrochemical currents detected in the NAc by substrate-specific and substrate-null sensors during long-term recordings in freely moving rats. Both GLU (A) and glucose (B) currents slowly decreased during ~8-hour in vivo recording. A similar decrease, but at lower absolute levels, occurred with both types of null sensors recorded during the same time *in vitro* at 22–23°C. Since active sensors differ from null sensors only by the presence of a specific enzyme (glutamate oxidase or glucose oxidase), the difference in currents detected by these sensors reflects the GLU or glucose contribution (red vertical lines in A and B). Open and close circles in A show current values in the NAc shell and core, while a solid line shows their average. C compares the proportion of the specific contribution of GLU and glucose (in red) with respect to nonspecific contributions (gray and blue) to the overall recorded current. While these nonspecific contributions are similar for both glutamate and glucose sensors, the specific component is much larger for glucose than for glutamate. Original data were published in [174,177]; the picture was reproduced in [187] (ACS Chemical Neuroscience; open access publication).

Taking into account the possibility of a strong confound of nonspecific chemical and physical interferents to currents detected by GLU and glucose sensors *in vivo*, we conducted multiple *in vitro* and *in vivo* recordings with null sensors that are identical to the active sensors but lack specific enzymes, which render them fully insensitive to either substrate (see Figures 17,18). The null sensors provided the best possible control for substrate specificity because they were equally sensitive to everything (i.e. temperature, ascorbate, DA, etc.) except the substance of interest. Therefore, the difference in currents detected by active and null sensors under the same conditions can provide a much more accurate measure of true stimulus-induced fluctuations in extracellular levels of GLU and glucose.

#### Basal levels of glutamate and glucose in the NAc extracellular space

It is generally believed that electrochemical techniques cannot provide reliable information on basal levels of neuroactive substances in the brain’s extracellular space. Typically, data are shown as either current or concentration changes with respect to undefined basal levels. However, the use of null sensors provided us with crucial data to resolve this issue.

To evaluate basal levels of extracellular GLU, we examined basal electrochemical currents detected by GLU and null sensors during long-duration *in vivo* recording (~8 hrs) in freely moving rats. As can be seen in Figure 18(a), the mean values of both currents recorded from the NAc changed similarly but currents detected by GLU sensors were consistently larger than those detected by null sensors. Taking into account this difference and the substrate sensitivity of GLU sensors at 37°C (~0.59 nA/1 μM), it appears that mean basal levels of extracellular GLU in the NAc are ~0.96 μM. Although basal currents for both GLU and null sensors slowly decreased during the session, reflecting a well-known property of electrochemical baseline, the decrease was proportional in both groups, suggesting that extracellular levels of NAc GLU remain relatively stable during long-term *in vivo* recordings. The absolute values of oxidation currents generated by null sensors at 22–23°C were much lower than those recorded by the same electrodes *in vivo*. This current difference reflects total nonspecific (non-GLU-related) chemical and physical *in vivo* contributions to electrochemical currents. As shown in Figure 18(c), these nonspecific contributions to the current (blue and gray areas) are proportionally much greater than those contributed by GLU (red area). By comparing this current difference with current increase that should occur *in vitro* with a 15°C temperature increase (~2.7 nA), it is evident that temperature alone makes much larger contribution to electrochemical currents than all other chemical factors (i.e. DA, DOPAC, ascorbate, urate, etc.) combined.

A similar analysis, a comparison of currents detected by glucose and null sensors during long-duration *in vivo* recording, was conducted to evaluate basal levels of extracellular glucose (Figure 18(b)). In this case, basal currents detected by glucose sensors in the NAc were also consistently larger than those detected by null sensors. Taking into account these differences and *in vitro* sensor sensitivity corrected for 37°C, mean values of basal glucose concentrations in the NAc were ~540 μM. These current differences remained similar (<10% difference) during long-term *in vivo* recording, indicating relative stability of extracellular glucose levels in awake, freely moving rats. A similar analysis conducted for data obtained from the *substantia nigra pars reticulata* revealed similar, albeit slightly lower, mean values of extracellular glucose (~407 μM [175]). Both these values are in line with microdialysis estimates, which vary in different structures from 0.35 to 1.5 mM [37,184–186]. As shown in Figure 18(c), actual glucose (red area) contributes much more to the measured glucose current than do all other nonspecific chemical and physical factors.

#### NAc glutamate response elicited by tail-pinch: Specific and nonspecific contributions

**Figure 19.**
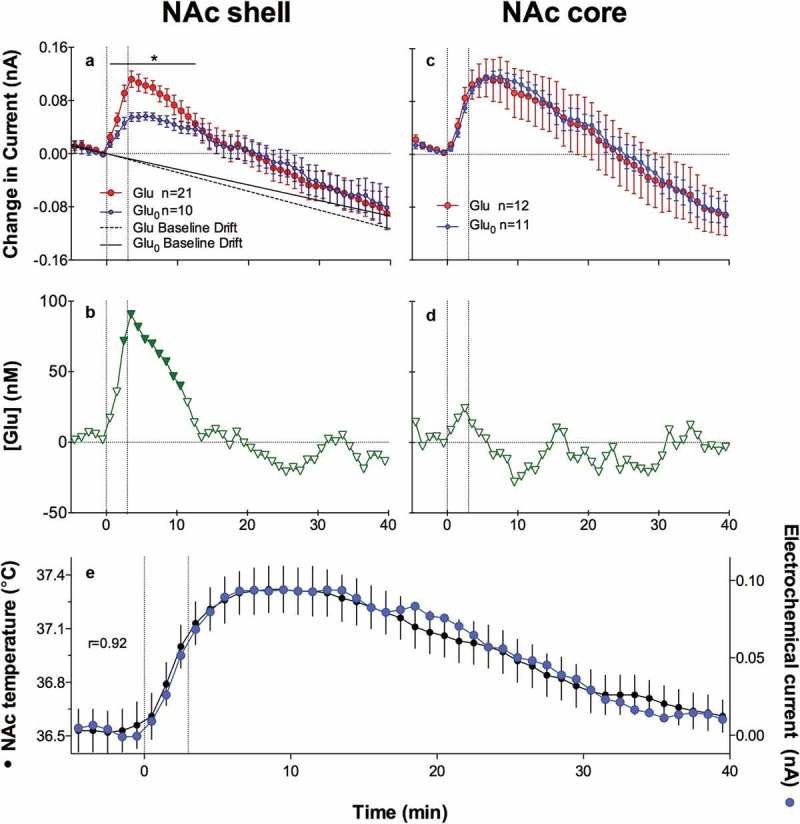
Slow changes in electrochemical currents detected by GLU and GLU-null sensors in the NAc shell (left panel) and NAc core (right panel) during a 3-min tail-pinch in freely moving rats. Top row (A and C) shows mean (±SEM) changes in total currents detected by both types of sensors. Middle row (B and D) shows current differences (active – null) calibrated in nM GLU concentrations. The two vertical hatched lines in each graph show onset and offset of tail-pinch and horizontal hatched lines show baselines. Two diagonal lines in A show the trend in current baselines calculated for GLU and GLU-null sensors. Asterisks in A show the period of significant differences in currents (current x time interaction assessed with two-way ANOVA) detected by active and null sensors. Significant differences in currents (active – null) are shown in B as filled symbols. E shows mean(±SEM) changes in NAc temperature and electrochemical current detected by GLU-null sensors during 3-min tail-pinch. Original data were published in [174,177]; the picture was reproduced in [187] (ACS Chemical Neuroscience; open access publication).

As shown in Figure 19(a), a 3-min tail-pinch elicited a significant increase in oxidation current detected by GLU sensors in the NAc shell. However, oxidation currents detected in this structure by null sensors also increased, confirming that only a certain portion of a total current change can be attributed to GLU. The subtraction procedure revealed (Figure 19(b)) that the actual change in GLU is much weaker in terms of concentration (90–100 nM or ~10% increase over baseline), more phasic, and significant for only ~10 min from the stimulus onset. Similar tests conducted in the NAc core revealed that currents detected by GLU-active and null sensors are virtually identical, with no significant between-group differences (Figure 19(c,d)). Therefore, in this structure tail-pinch did not induce significant changes in extracellular GLU levels. As shown in Figure 19(e), current changes detected in the NAc shell by a null sensor during tail-pinch were tightly related to changes in NAc temperature (r = 0.92). A similarly strong correlation was found for other arousing stimuli, including social interaction and novel object presentation. Therefore, the null sensors could be a critical tool for excluding nonspecific contribution to electrochemical currents recorded by enzyme-based GLU sensors *in vivo*. This approach reveals that real changes in extracellular GLU levels are much weaker and more phasic than those evaluated based on total currents detected by GLU sensors. This procedure also makes it possible to eliminate the negative trend in baseline currents that is evident with any type of long-term electrochemical recording.

#### NAc glucose response to arousing stimuli

**Figure 20.**
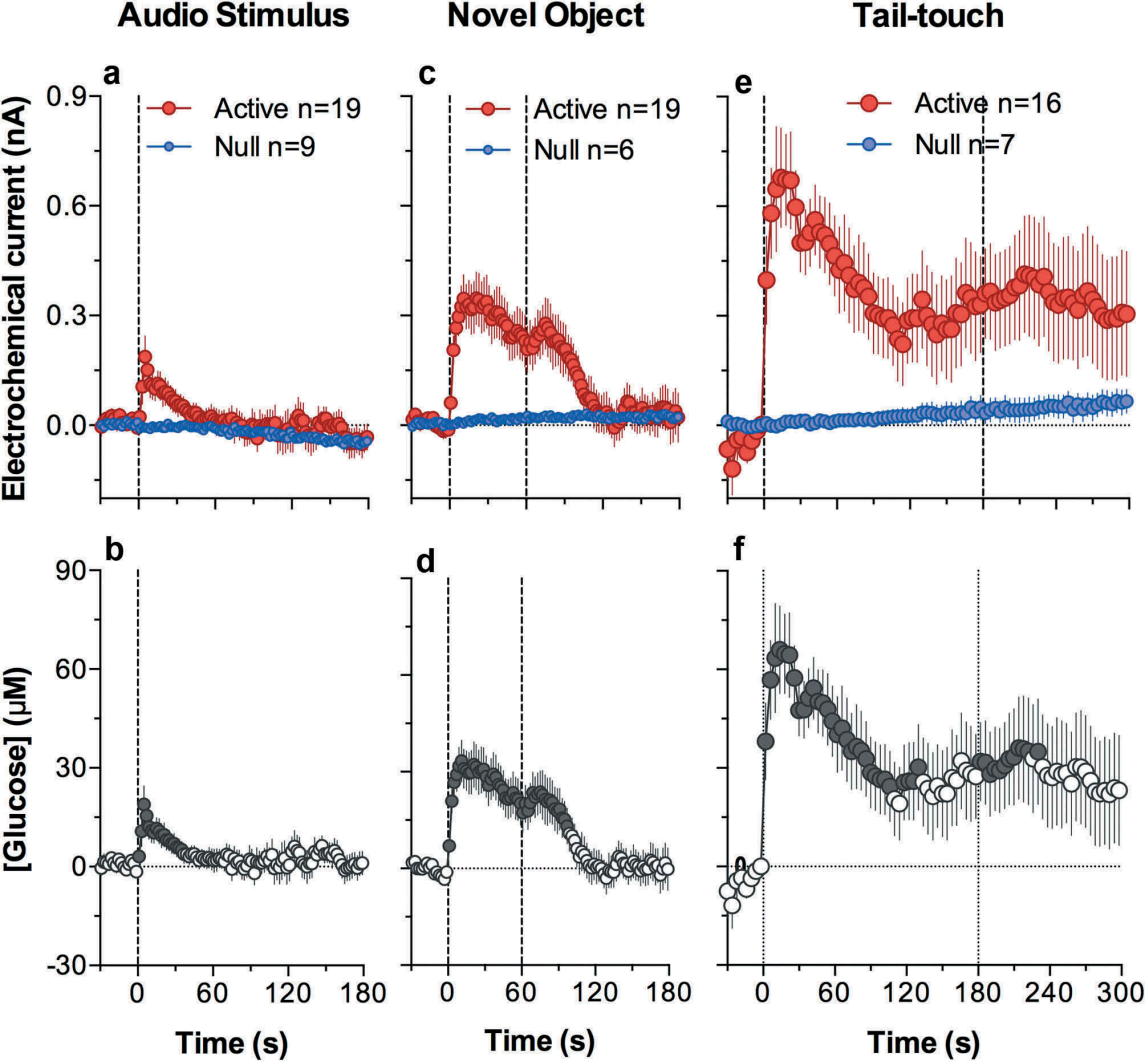
Mean (±SEM) changes in electrochemical currents detected by glucose and glucose-null sensors (A, C, E) and resulting changes in [glucose] (B, D, F induced by different sensory stimuli in freely moving rats. Data for audio stimulus and novel object are shown with 2-s time resolution, and tail-touch shown with 4-s time resolution. Original data were published in [175]; the picture was reproduced in [187] (ACS Chemical Neuroscience; open access publication).

A similar approach was used to examine physiological changes in extracellular glucose levels [175,176,188]. Figure 20 shows changes in electrochemical currents detected by glucose and null sensors in the NAc shell elicited by several types of arousing stimuli: a 0.5-s sound tone, presentation of a novel object, and tail-pinch. In contrast to the relatively weak and slow current increases detected by null sensors, current increases detected by glucose sensors were much larger in magnitude and much more phasic. Subtraction of these nonspecific currents revealed that NAc glucose levels rapidly jump within seconds after the onset of each stimulus but show different dynamics thereafter.

Therefore, the glucose-specific contribution to electrochemical currents detected by glucose biosensors greatly exceeds nonspecific contributions as detected by null sensors. In this case, the use of null sensors appears less critical compared to studies of GLU because of the much higher basal levels of glucose in extracellular space (~500 μM vs. ~1 μM for GLU) and their relatively large changes induced by arousing stimuli (50–100 μM vs. 50–100 nM). Due to the high glucose levels in the brain’s extracellular space, current changes induced by its enzymatic oxidation are much larger than those induced by all other physical and chemical contributors.

### Temperature influences on microdialysis evaluations of brain neurochemistry

The technique of *in vivo* microdialysis is based on diffusion as a form of passive transport, during which particles travel down a concentration gradient between the brain’s extracellular space and the content of perfusate flowing in the dialysis probe [189,190]. While diffusion rate depends on multiple factors (i.e. the nature of the membrane and particles, concentration gradient, rate of dialyzate flow), temperature appears to be an important factor affecting the entry of substances from the brain’s extracellular space into dialyzate (i.e. recovery). Careful *in vitro* studies have revealed that recovery of monoamines strongly depends on temperature of the perfusate. With perfusate at 37.5°C, the recovery was 49%, 48% and 37% for norepinephrine, DA, and serotonin, but it dropped to 18%, 20%, and 17% when the temperature of perfusate was 24°C [191]. This influence could be minimized when the temperature of solution flowing in the dialysis probe is close to 37°C–average brain temperature. However, this temperature factor cannot be fully eliminated when brain temperature naturally fluctuates in freely moving animals following exposure to arousing stimuli or drugs. While this contribution could be viewed as small (for DA 18%/1°C for recovery rate [191];), physiological increases in brain temperature could vary within 1–2°C, producing a measurement artifact, which for DA could be within 18–36%. These values are comparable with DA increases induced in the NAc by tail-pinch (+20% [192],; +25% [193], +30–40% and 25% [194,195]), which elicits a ~ 1°C brain temperature increase (see above). Therefore, it appears that brain temperature increases could be a serious contributor to reported increases in extracellular levels of DA and other neuroactive substances induced by different arousing stimuli and drugs that increase brain temperature. Importantly, the influence of this factor cannot be eliminated, and it is unclear how concomitant changes in brain temperature can be controlled during microdialysis experiments.

Further evidence for the role of brain temperature as a factor affecting microdialysis measurements was obtained in experiments employing forced-swim stress in mice and rats. While in some studies animals are immersed in warm water, in others animals are exposed to water at room or colder temperature (22–25°C) that results in robust decreases in their body [196,197] and brain [198] temperatures. These decreases depend on the temperature of the water and duration of exposure, but they are always robust and fluctuate from 12°C to 15°C [196,199] to 6–8°C at warmer water temperatures and shorter exposures [198,199]. Assessment of NAc DA during the forced-swim test in cold water (22°C) revealed a three-fold drop in its dialyzate concentration (~30% of baseline), while swimming in warmer water (32°C) did not reveal significant changes in DA levels [200]. Similar, even more profound, qualitative changes were found with GABA, as its levels in the hippocampus decreased (to 70% of baseline levels) during swimming in 25°C water and clearly increased (+50% above baseline) during swim in 35°C-water [197]. In contrast to cold-water swimming, which induces robust brain and body hypothermia, warm-water swimming did not result in evident changes body temperature changes [197] or it induced a slight (0.5°C) increase in NAc temperature [198].

### Conclusions

The basic premise of any neurochemical measurement technique is its ability to provide a precise quantitative evaluation of changes in concentrations of a substance of interest, without any contribution of other, nonspecific chemical and physical factors. While this goal could be approached by the creation of highly sensitive and selective electrochemical sensors and sophisticated analyses to detect minute amounts of substances in dialyzates, there are natural limitations of these measurements when conducted in awake, behaving animals. These limitations could be especially serious when physiological or drug stimuli induce strong changes in brain temperature, which can cause an undervalue or overvalue of the measured neurochemical parameters. When not given due consideration, regardless of the sensor’s chemical sensitivity and selectivity, exceptional analytical potential of current-state microdialysis, and rapidity of measurement, the detected changes could be erroneously attributed to increases or decreases in extracellular levels of the substance of interest.

## Brain hyperthermia due to environmental heating, excessive bodily heat production, and insufficiency of heat dissipation

In contrast to physiological increases in brain temperature that are triggered by neural activation and have an adaptive significance, brain temperature can also increase during excessive physical activity and/or under conditions that prevent proper heat dissipation to the rest of the body and then to the external environment. In this case, brain hyperthermia results from a rise in body core temperature and subsequent warming of arterial blood, thus decreasing the efficiency of heat dissipation from brain tissue and subsequently increasing brain temperature.

It is well established that intense physical activity is associated with robust energy consumption and significant heat production. As assessed in human volunteers, transition from quiet rest to intensive running results in up to a ten-fold rise in whole-body oxygen consumption [5,201], corresponding to about a ten-fold increase in whole-body heat production [202]. However, due to a well-developed ability to sweat and a dynamic range of blood flow rates in the skin [203], this enhanced heat production is compensated for by enhanced heat loss. Sweat rates during intense physical activity may reach up to 2.0 l/h, providing a potential evaporative rate of heat loss in excess of 1 kW (or ~14 W/kg), i.e. more than the maximal possible heat production. While these compensatory mechanisms can provide relative stability of brain and body temperature, they become progressively less effective in hot and humid conditions, resulting in progressive heat accumulation in the organism. Body temperatures measured at the end of a marathon run on a warm day could increase as high as 40°C [204] and cases of fatigue during marathon running are associated with even higher body temperatures [205]. Body temperature increased less than 1°C during intense cycling in experienced healthy cyclists at normal environmental temperatures, but much larger, 2.0–2.5°C (or 39.5–40.0°C), increases occurred when the same cycling was done in water-impermeable suits that restrict heat loss via skin surfaces [86].

Body hyperthermia associated with intense physical activity under conditions that restrict heat dissipation is a known phenomenon, but the impact of these conditions on the brain is a matter of controversy [206–209]. By using tympanic temperature as an indirect measure of brain temperature [210,211], it was assumed that brain temperatures during intense body heat production remain lower than body temperatures, suggesting selective brain cooling as a mechanism that prevents brain over-heating during extreme body hyperthermia. However, direct measurements of brain temperature in neurological patients [64,122–124] and applications of more sophisticated physiological recordings [86,212] called the existence of this protective brain cooling mechanism in humans into question. Direct temperature measurements in the carotid artery and internal jugular vein in human volunteers revealed that at rest, venous blood exiting the brain is ~0.3°C warmer than arterial blood entering the brain. Although both temperatures increased about 1°C during intense cycling, the increase was stronger in the carotid artery than in the jugular vein, and the venous-arterial difference always remained positive. When the cycling was done in water-impermeable suits, the increases were more dramatic (up to 39.3°C and 39.5°C for arterial and venous blood, respectively), but the difference still remained positive. After termination of exercise, arterial blood temperature rapidly dropped, while venous temperature decreased more slowly. Simultaneous measurements of tympanic temperatures under these conditions revealed that their values are consistently lower than, and independent of, arterial and venous temperatures. Therefore, during intense physical exercise coupled with restriction of heat dissipation, brain temperature in humans may also gradually increase, reaching pathological levels. Although arterial blood always remains cooler than brain tissue and removes metabolic heat from the brain, progressive intra-brain heat accumulation occurring during intense physical activity reflects a failure of this natural cooling mechanism due to an inability to dissipate metabolic heat to the external environment and the gradual warming of arterial blood arriving to the brain.

Since direct measurements of brain temperature in humans are usually not possible and rare, temperatures of venous blood exiting the brain may be viewed as the best indirect measure of brain temperature. However, direct measurements from the brain, jugular vein, and body core (rectum) in neurological patients revealed unexpected differences between these areas [64,122–124]. These studies showed that temperature in the cortex is 0.2–0.8°C higher than in the rectum and ~1.0°C higher than in the jugular vein. Although jugular vein temperatures are often viewed as the best proxy of brain temperature in humans, jugular temperatures correlated more strongly with rectal but not brain temperature. Finally, these relationships were valid not only for normothermic conditions but also during fever, further suggesting that the human brain has no specific protection against thermal impact.

The relative independence of brain temperature from core body temperature appears to be of clinical importance for ischemic and traumatic brain damage in humans. In contrast to normal conditions, in which brain temperature is close to core body temperature, cortical temperature (measured intraparenchimally) in patients with head trauma is about 1°C higher and relatively independent of both rectal and jugular temperatures [64]. Because of the dorso-ventral temperature gradient [62,63], deep brain structures may be 0.6–1.0°C warmer than superficial structures, or almost two degrees warmer than the body core. Importantly, this relative brain hyperthermia is maintained despite a decrease in brain metabolism and intra-brain heat production, presumably because of serious impairment of systemic and local cerebral blood flow, which normally removes excessive heat from brain tissue. Since both traumatic and ischemic brain injuries are strongly enhanced at higher temperatures [213,214], this relative brain hyperthermia, coupled with a diminished ability for temperature regulation and no specific mechanisms for human brain protection against thermal stress [123], makes these patients especially sensitive to fever and even modest environmental heating.

## Brain temperature fluctuations induced by neuroactive drugs of abuse

In contrast to natural arousing stimuli, which induce neural activation via stimulation of specific sensory receptors and rapid neural transmission to the CNS via somatosensory pathways, neuroactive drugs induce their central effects via direct interaction with peripherally and centrally located neural substrates that are also involved in detection of natural stimuli and the triggering of adaptive responses to these stimuli. In contrast to therapeutic neuroactive drugs, which are taken voluntarily or given by medical professionals to alleviate specific pathological symptoms, individuals also take some drugs recreationally to induce pleasurable, novel, or unusual psycho-emotional effects. Due to repeated use of these drugs and the fixation in memory of their neural effects, drug intake becomes the critical event of motivated drug-seeking and drug-taking behavior and the drugs are self-administered at higher doses and often via more aggressive (i.e. iv) routes.

In addition to individual predisposition, each drug of abuse has its own potential for the development of addictive behavior. Below I will consider some findings obtained using multi-site thermorecording with cocaine and heroin – two drugs with high addictive potential. I will also present our data regarding thermogenic effects of MDMA, a drug with much weaker addictive potential, and the dependence of these effects on activity state and environmental conditions. MDMA is considered a “club drug,” which is typically used by young individuals at “drug parties,” often in high activity states and in warm environments that diminish natural abilities for heat dissipation. This drug at low doses induces weak physiological effects and thus appears to be physiologically safe, and it is considered a possible tool to treat post-traumatic stress disorder (PTSD) and some types of depression in humans [215,216]. However, heavy use of MDMA in the context of drug parties often results in serious health complications, including pathological hyperthermia and death.

### State-dependent effects of cocaine: Cocaine as an arousing stimulus and drug reinforcer

Cocaine is a widely used drug of abuse, which acts on multiple neural substrates both in the CNS and periphery [217,218]. Early neurochemical studies revealed that cocaine is a nonselective inhibitor of reuptake of monoamines [219-221] and cocaine’s action on monoamine transporters is generally viewed as the critical mechanism mediating its psychoactive and reinforcing properties. Due to its effects on reuptake of monoamines, neural effects of cocaine should depend on the ongoing activity of monoamine neurons and ongoing release of monoamines, which are affected by an organism’s functional state and various environmental stimuli. The psycho-physiological activation appears to be an essential part of cocaine-seeking behavior and it could dramatically alter the effects of this drug observed in drug-naive animals during quiet resting conditions. In our studies on cocaine, we first examined how brain temperature is affected by cocaine administered to quietly resting animals at an optimal self-administering dose [222]. Then, we examined patterns of temperature fluctuations occurring during cocaine self-administration in drug-experienced, trained rats [223]. Finally, we conducted a yoked-control experiment, in which cocaine was delivered passively at the doses and in the pattern seen during drug self-administration [224]. This experiment allowed us to distinguish the relative contributions of the drug itself vs. behavior (drug seeking and taking) to temperature changes occurring during cocaine self-administration.

**Figure 21.**
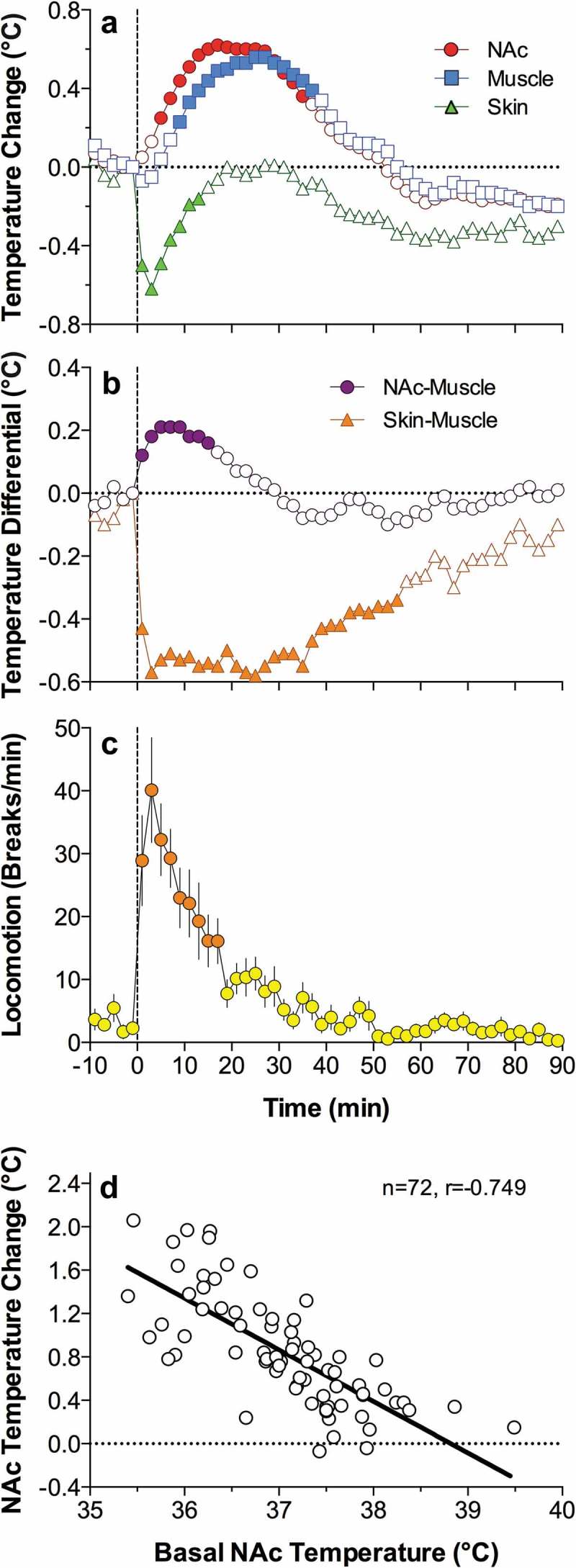
Changes in NAc, muscle, and skin temperature induced by iv cocaine (1 mg/kg) in freely moving rats. A = temperature changes vs. pre-injection baseline; B = temperature differentials; C = locomotor activity; D = relationships between the magnitude of cocaine-induced NAc temperature elevation and basal temperature; n = number of analyzed responses; r = coefficient of correlation. Regression line crosses the line of no effect at ~39.5°C. Data were replotted from reference [222].

When delivered to quietly resting rats intravenously at the dose (1 mg/kg) optimal for self-administration, cocaine moderately increased brain and muscle temperatures and induced a biphasic, down-up fluctuation in skin temperature (Figure 21(a–c)) – the response pattern similar to that induced by arousing stimuli (see above). The temperature increase in the NAc was more rapid and stronger than in the temporal muscle, suggesting intra-brain heat production due to metabolic brain activation as a contributor to hyperthermic effects of cocaine. Due to distinct changes in skin and muscle temperatures, the skin-muscle differential rapidly decreased, suggesting peripheral vasoconstriction as another major contributor to brain and body temperature increases. Similarly as with natural arousing stimuli, the hyperthermic effects of iv cocaine were strongly dependent on baseline temperatures (Figure 21(d); r = −0.75). At lower basal NAc temperatures (35.5–37.0°C), cocaine increased them strongly, but the effect became progressively weaker when basal temperatures were higher. Based on the results of regression analyses, cocaine increases brain temperature when it is lower than ~38.5°C° and its ability to increase it disappears at values larger than 38.5–39.0°C. Therefore, under certain conditions when brain temperature before cocaine administration is high, cocaine can decrease brain temperatures – a response that is opposite to that occurring under quiet resting conditions at low brain and body temperatures.

Much more complex temperature changes were found during cocaine self-administration [223,224]. Rats in these experiments were trained to press a lever that delivered iv cocaine (1 mg/kg). Each lever-press was paired with a combined sensory stimulus (light + sound) and triggered a ~ 20-s cocaine injection. Consistent self-administration was typically developed within 3–4 daily sessions and temperature recordings were conducted in trained rats. After a ~ 2-h habituation to the recording chamber when the lever was blocked, rats were exposed to a sensory stimulus and the lever became accessible and operational. Subsequent behavior was determined by the rat. Typically, the recording session continued for ~4 h and rats received ~20–30 self-injections with 5-8-min inter-injection intervals. After the last self-injection, the lever was blocked, cocaine became unavailable, and the recording continued for at least two more hours. Within a session, one lever-press resulted in a doubled injection (2 mg/kg) and one lever-press was not reinforced. Another group of rats (yoked control) received the same drug treatment but all injections (paired with light + sound stimulus) were delivered passively with the same inter-injection intervals (5–8 min).

**Figure 22.**
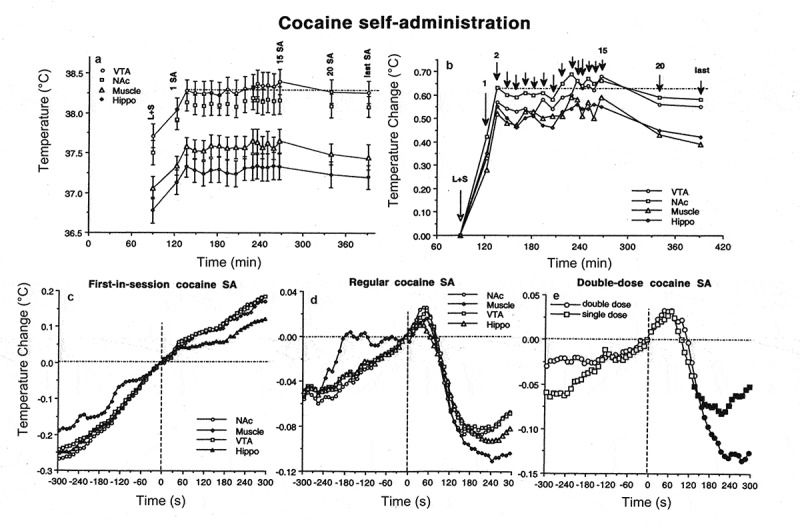
Changes in brain (NAc, nucleus accumbens; VTA, ventral tegmental area of midbrain, Hippo, hippocampus) and muscle temperatures during cocaine self-administration in trained rats. A and B = changes in absolute and relative temperatures averaged for each consecutive cocaine self-injection. L + S, the moment of light+sound presentation, when the lever became accessible and the rat could press a lever. C = temperature changes associated with the first-in-session cocaine self-injection. D = temperature changes associated with regular cocaine self-injections. E = differences in NAc temperature changes after a typical single-dose (1.0 mg/kg) and double-dose (2.0 mg/kg) cocaine self-injections. Data were replotted from [223].

During the first stage of our analyses, we determined absolute and relative changes in brain (NAc, ventral tegmental area of the midbrain or VTA, and hippocampus) and muscle temperature during the entire session of cocaine self-administration (Figure 22(a–b)). Each point in these graphs represents mean temperature values immediately before the presentation of the light + sound sensory cue and each subsequent cocaine self-injection of a session. As can be seen, brain temperature in each location increased after exposure to the cocaine-related cue and further increased after the first self-injection of a session. While the rat continued to self-inject cocaine, brain temperature stabilized at ~38.3°C and was maintained at these levels until the moment of the last self-injection. Despite differences in basal temperature, the pattern of tonic changes was similar in each of three brain structures.

**Figure 23.**
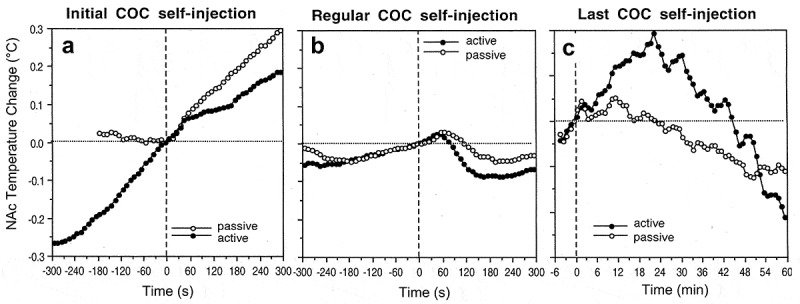
Changes in NAc temperature associated with the initial cocaine self-injection of a session (A), subsequent, regular self-injections (B) and the last self-injection of a session, when the operant level was blocked and drug was unavailable (filled symbols). Each graph also shows the changes associated with the same events when cocaine was injected passively (yoked-control) (open circles). Data were replotted from [223,224].

A more complex picture of brain temperature fluctuations was revealed when data were analyzed with high temporal resolution preceding and following each of the critical behavioral events (light + sound presentation, the initial self-injection of a session, regular self-injections, double-dose self-injection, non-reinforced lever-press, last self-injection of a session). As can be seen in Figure 22(c–e), the cocaine-induced temperature increase following the first self-injection of a session was preceded by an equally strong temperature increase that was induced by a cocaine-related sensory cue that triggered drug-seeking behavior. This gradual temperature increase that preceded the initial self-injection was absent in yoked-control animals that passively received the initial cocaine injection (Figure 23(a)). However, this passive injection induced an equally strong and even slightly larger post-injection temperature increase, with more rapid and stronger changes in each brain structures vs. temporal muscle. Therefore, a gradual increase in brain temperature elicited in behaving rats by the sensory stimulus previously associated with cocaine could be viewed as both a conditioned temperature response and a correlate (or driving source) of cocaine-seeking behavior elicited by this conditioned stimulus.

In contrast to monophasic temperature increases induced by the initial self-injection of a session, clear biphasic fluctuations of much smaller magnitude (~0.1°C) were typical of all subsequent self-injections in the session (Figure 22(d)). In these cases, brain temperature gradually increased before the lever-press for cocaine, peaked within 60 s after the injection onset, and transiently decreased thereafter. Despite their relatively small magnitude, these biphasic fluctuations were highly significant and occurred at higher but relatively stable tonically increased brain temperatures (Figure 22(a)). When instead of a regular 1.0 mg/kg injection, the injection’s dose was doubled, the response pattern remained the same, but the post-injection decrease became stronger and more prolonged (Figure 22(e)). Interestingly, similar biphasic temperature fluctuations also occurred during repeated passive cocaine injections, but they were half as large in magnitude (Figure 23(b)). Similarly as with the self-injection, brain temperature gradually increased preceding passive cocaine self-injection, peaked within ~60 s from the injection start, and decreased thereafter. Therefore, the observed biphasic fluctuations in brain temperature occurring during cocaine self-administration are determined not only by drug-seeking and drug-taking behavior but also by superimposition of repeated drug effects. Importantly, the post-injection decrease in brain temperatures began 40–60 s after the start of cocaine injections, obviously reflecting the time necessary for the drug to travel to the brain, cross the BBB, diffuse to centrally located neural substrates and interact with these substrates [225]. Although brain temperature increases for ~20 min after a single passive injection (see Figure 21), trained rats self-administer this drug at the same, 1 mg/kg dose, with a mean inter-injection interval of 7–8 min, meaning that each subsequent injection occurs within the time frame of previous drug effect. Therefore, this temperature decrease appears to be a true central effect of cocaine occurring due to its interaction with centrally located neural substrates, presumably monoamine transporters. Importantly, this effect appears following repeated drug administration with inter-injection intervals that are chosen by a behaving rat (~7 min) and after basal temperature is strongly increased (>38.0–38.5°C or 1.5–2.0°C above quiet resting baseline) by previous cocaine injections.

Finally, brain temperature strongly increased after the last cocaine self-injection of the session, when the lever was blocked and the rat could not receive drug injection despite multiple attempts to activate the blocked lever (Figure 23(c)). This effect was seen only in behaving animals and was absent in rats that received cocaine passively. In this latter case, NAc temperature slowly returned to the initial baseline. Therefore, this increase in brain temperature following blockade of the operant lever is primarily a correlate of drug-seeking activity aimed to receive cocaine.

**Figure 24.**
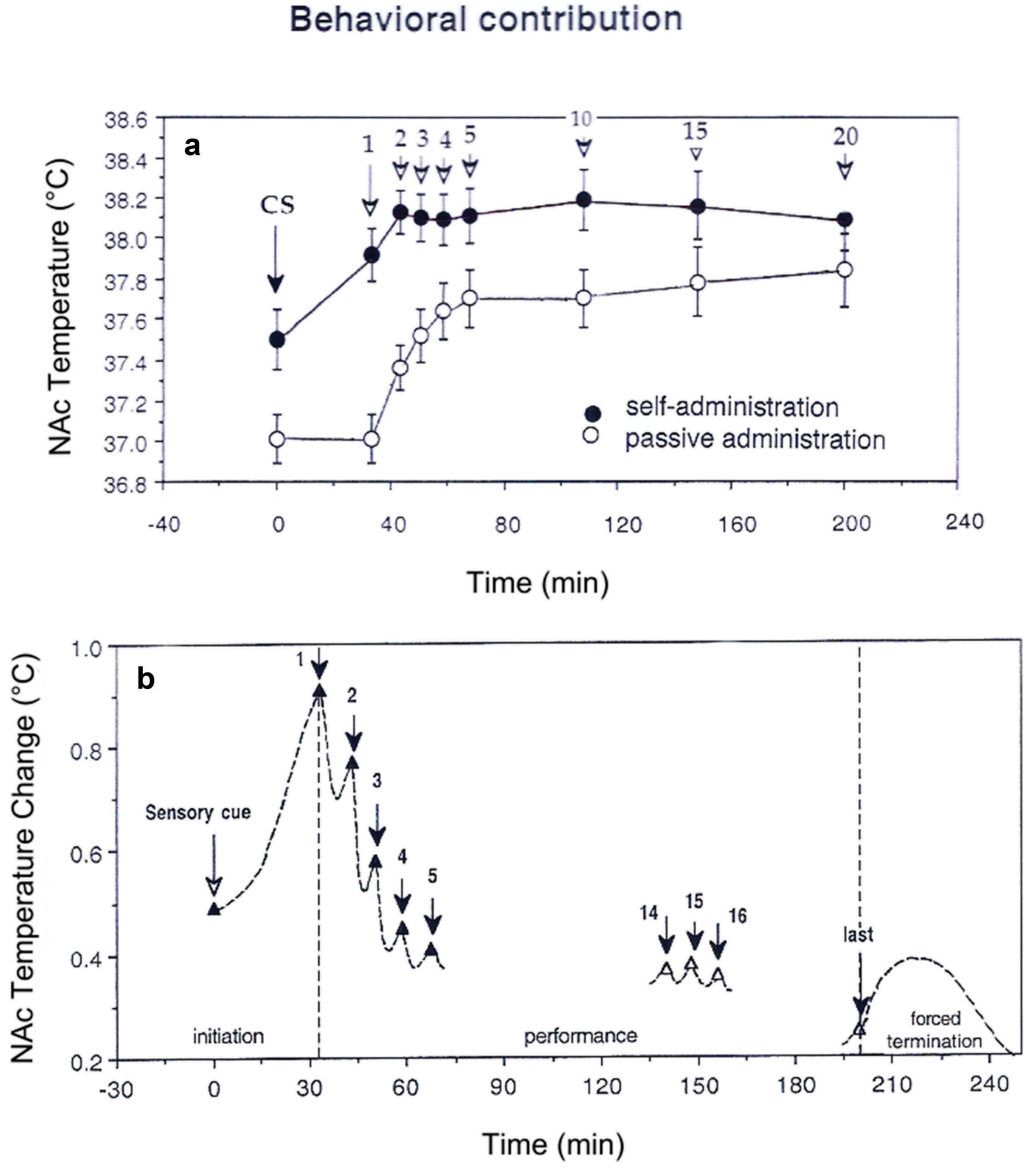
Behavioral contribution to NAc temperature changes during cocaine self-administration in trained rats. Top graph (A, black symbols) shows mean values of NAc temperature immediately before each critical behavioral event (CS, light+sound conditioned cue, arrows with numbers are consecutive cocaine self-injections. White symbols shown NAc temperature values associated with the same events in yoked-control animals. B shows differences in NAc temperature between active and passive administration of cocaine. Filled triangles show values with significant between-group differences. Picture is based on re-analysis of original data presented in references [223,224].

To determine the contribution of behavior to changes in brain temperature occurring during cocaine self-administration, we compared NAc values associated with critical behavioral events in rats self-injecting cocaine and receiving this drug passively (Figure 24). These data are shown as changes in absolute temperatures (**A**) and as a temperature difference between active and passive rats (**B**). As can be seen, animals with previous self-administration experience had significantly higher NAc temperature before the presentation of the cocaine-related sensory cue (or conditioned stimulus, CS) than rats exposed to the same cocaine treatment passively. Active rats also showed stronger temperature elevation after exposure to this cue, while passive animals did not show significant changes. The largest, almost one degree, difference in NAc temperature between active and passive animals was found immediately before the first cocaine injection, which in active rats occurred due to seeking behavior and in passive rats occurred 30 min after the presentation of the light + sound cue. While temperature slightly increased after the first cocaine self-injection, it was stabilized following subsequent self-injections, which were associated with biphasic down-up temperature fluctuations. In passive animals, NAc temperature also increased following the first cocaine injection and slightly increased following several subsequent injections, but then also stabilized and was maintained at the same levels despite further injections. However, the difference between active and passive animals slowly decreased within a session and eventually almost disappeared at the session end. Active animals also showed an additional temperature increase after the lever was blocked and the rat tried to press the blocked lever.

Therefore, to the extent that these brain temperature fluctuations reflect alterations in brain metabolic activity, our data suggest that cocaine in the context of drug-taking behavior is able to both induce generalized neural activation and to transiently inhibit the preceding drug- and behavior-related neural activation. While internal factors and conditioned influences appear to play a crucial role in triggering drug-seeking behavior that results in the first lever-press and initial cocaine delivery, further drug-taking behavior appears to be primarily pharmacologically determined, resulting from the biphasic, time-dependent effects of injected cocaine on neural activity. Therefore, intravenously delivered cocaine is able to both transiently inhibit drug seeking by abruptly blocking preceding drug- and behavior-related neural activation and promote drug seeking by inducing activation again, thus driving the subject to a new cycle of drug-seeking, finally resulting in the next drug intake.

### State-dependent effects of heroin: Heroin as a drug reinforcer

**Figure 25.**
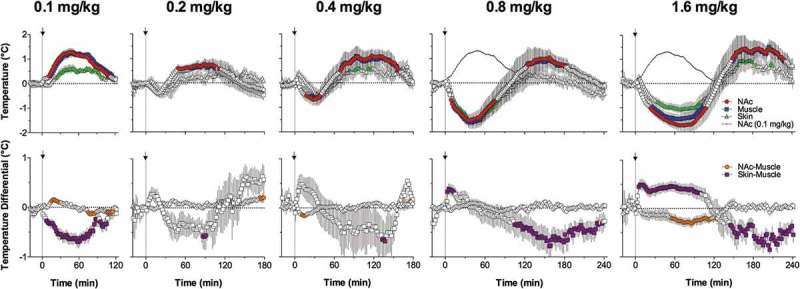
Changes in NAc, muscle, and skin temperature induced by iv heroin at different doses in freely moving rats. At the smallest dose that is optimal for drug self-administration, heroin induces monophasic increases in brain and muscle temperature. However, with increase in doses, the response became biphasic, with the initial, dose-dependent temperature decreases and subsequent temperature increases. Data were replotted from [228].

While cocaine is a psychostimulant drug, which induces sympathetic activation and locomotor hyperactivity, heroin causes profound sedation, locomotor hypoactivity and, at higher doses, a comatose state that can result in death [226,227]. Therefore, it could be assumed that heroin should induce brain hypothermia – a response opposite to that induced by cocaine and other psychostimulants. However, we found that iv heroin passively administered to awake, quietly resting rats at a low self-administering dose (0.1 mg/kg) induces moderate increases in NAc and muscle temperatures (Figure 25) that were comparable in magnitude but longer in duration than those induced by iv cocaine [228]. While the increases in brain and muscle temperatures were generally correlative, their difference, the NAc-muscle differential, significantly increased between ~10 and ~50 min post-injection, with a peak at ~20 min (Figure 25). This change suggests weak and delayed metabolic activation and differs from much stronger and more rapid increases in this parameter seen with iv cocaine and natural arousing stimuli. Heroin also increased skin temperature, but this effect was weaker than the increases seen in the brain and muscle. This skin temperature increase was also preceded by a transient temperature drop that occurred immediately after injection. Due to distinct temperature dynamics in the temporal muscle and skin, the skin-muscle differential rapidly and robustly decreased for ~100 min post-injection (Figure 25), suggesting strong peripheral vasoconstriction as a major cause of heroin-induced brain hyperthermia. This effect appeared very rapidly (0–2 min), peaked at 40–50 min, and was maintained for ~100 min. Similar to that seen with cocaine, the extent of heroin-induced NAc temperature increases was dependent on basal brain temperature, showing larger increases at low and smaller increases at high basal temperatures. This correlation was significant (r = −0.37, p < 0.05) but much weaker than that seen with cocaine.

The pattern of temperature effects of heroin differed depending on the drug’s dose (Figure 25). At low doses (within the optimum range for rat self-administration), heroin induced a monophasic hyperthermic effect, but with increases in doses, the NAc temperature response became biphasic, with rapid transient decreases after injection and subsequent more prolonged increases. Importantly, the initial hypothermic effect became substantially larger with dose increases, but the hyperthermic phase remained relatively stable. Similarly, the monophasic decrease in skin-muscle differentials seen at small doses became biphasic, with a prominent increase following injection, suggesting peripheral vasodilation as an immediate effect of high-dose iv heroin administration. Dose-dependent changes in brain-muscle differentials were less evident but this parameter significantly decreased at the 1.6 mg/kg dose. Therefore, similar to cocaine, which under different conditions is able to either increase or decrease brain temperature, iv heroin at different doses is able to induce opposite temperature responses.

**Figure 26.**
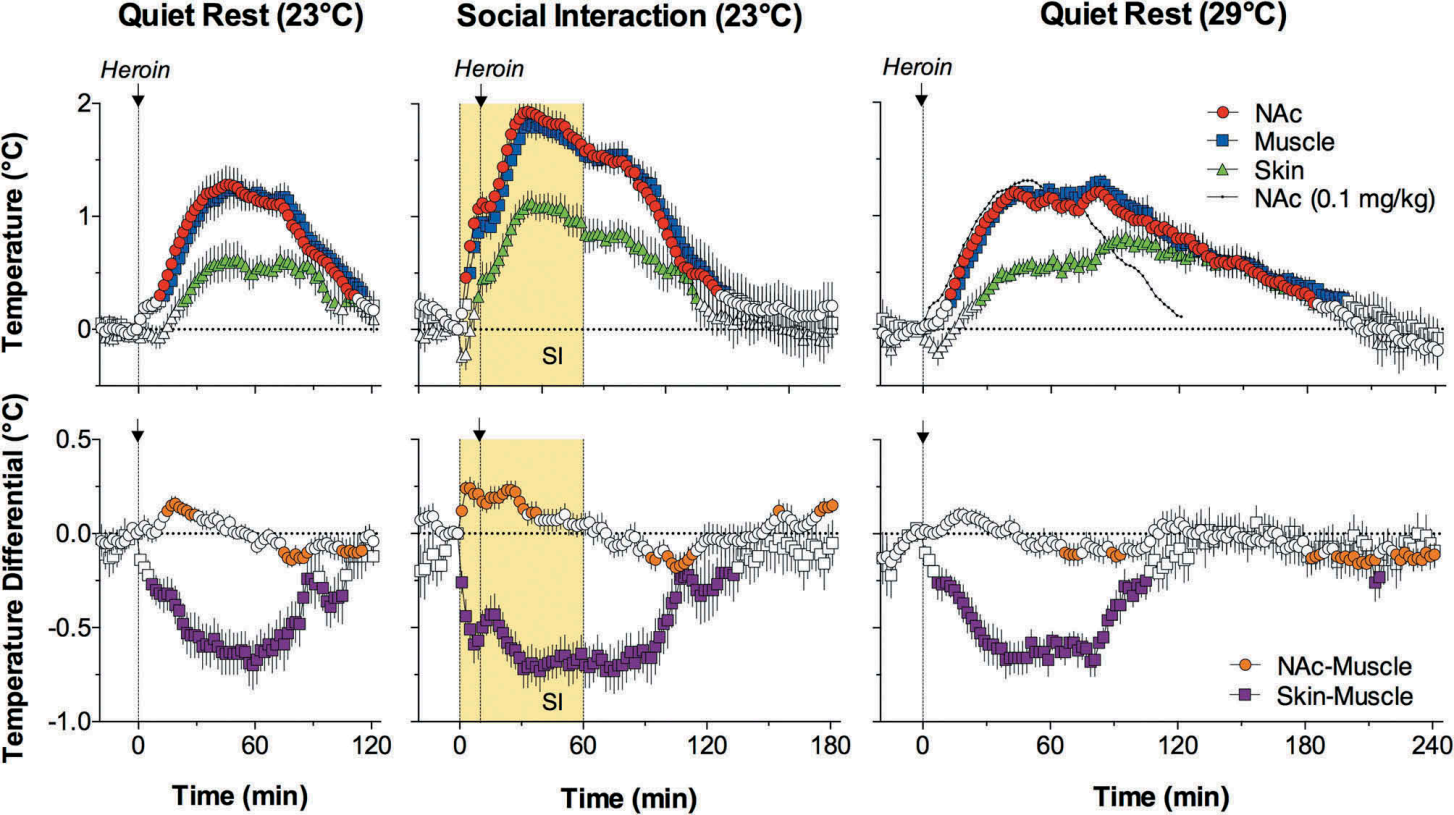
State-dependency and environmental modulation of temperature responses induced by iv heroin (0.1 mg/kg) in freely moving rats. When heroin as injected during social interaction (yellow area from 0 to 60 min), temperature elevation and decrease in skin-muscle differentials became larger compared to the effects induced by this dug in quiet resting conditions. When heroin was injected at 29·C ambient temperatures, drug-induced temperature elevation became more prolonged. Data were replotted from [228].

Brain temperature effects of iv heroin also depend on the animal’s activity state and environmental conditions (Figure 26). When heroin (0.1 mg/kg, iv) was administered during social interaction, a procedure that induces brain hyperthermia, the temperature increase became almost two-fold larger in magnitude and longer in its duration. Comparison of these data with those obtained with saline injections during social interaction revealed that the effect of heroin *per se* remains very similar and the increased temperature response reflects a summation of the two positive effects induced by social interaction and heroin. The temperature effect of heroin was also altered when it was injected in warm ambient temperatures (29°C), which is close to normothermia. In this case, the temperature elevation was similar in magnitude to that seen under standard laboratory temperatures (23°C), but its duration became much longer, obviously reflecting diminished heat dissipation in warm environments.

**Figure 27.**
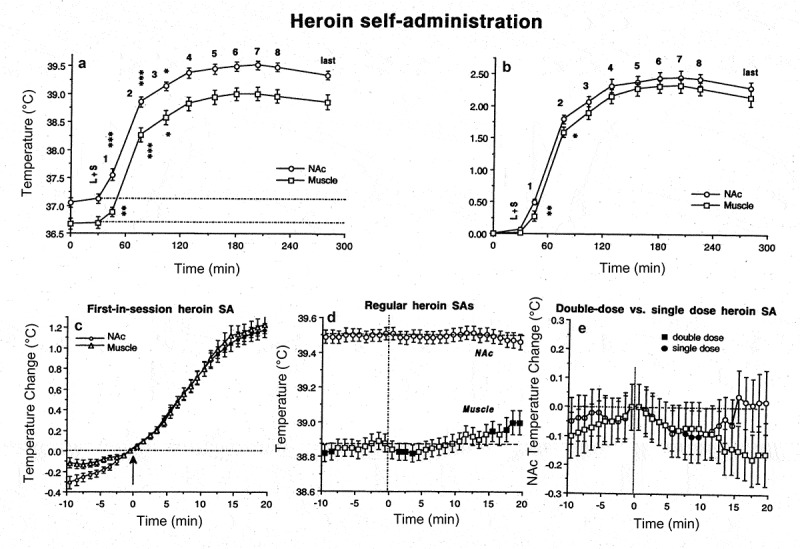
Changes (mean±SEM) in brain (NAc, nucleus accumbens) and muscle temperatures during heroin self-administration in trained rats. A and B = changes in absolute and relative temperatures averaged for each consecutive drug self-injection of a session. L + S = the moment of light+sound presentation, when the lever became accessible and the rat could press a lever. Asterisks show values significantly different from the previous value. C = temperature changes associated with the first-in-session heroin SA (arrow). D = temperature changes associated with regular heroin self-injections (filled symbols indicate values significantly different from the last pre-lever-press value (hatched line). E = differences in temperature changes after a typical single-dose (0.1 mg/kg) and double-dose (0.2 mg/kg) heroin self-administrations. Data were replotted from [229].

Similar to cocaine, the effects of heroin were strongly dependent upon the behavioral context of drug administration, showing a complex pattern of changes during iv heroin self-administration [229]. Similar to our cocaine self-administration study (see above), rats were trained to press a lever which resulted in delivery of a 0.1 mg/kg dose of heroin coupled with a light + sound stimulus. Temperature recordings were conducted in moderately trained animals (4–6 previous sessions). In the beginning of a session, after ~2-h habituation to the recording chamber (when the lever was blocked and inaccessible), rats received the light + sound stimulus and subsequent behavior was entirely determined by the rat. As shown in Figure 27(a), NAc and muscle temperatures in trained rats strongly increased after the initial heroin self-injection of a session. Similar as with cocaine, this increase was preceded by a temperature increase that occurred after the rat was exposed to a heroin-related sensory cue that triggered drug-seeking behavior (Figure 27(c)). A weaker increase occurred after the second self-injection but then temperature stabilized at elevated levels (39.0–39.5°C or 2.0–2.5°C above the baseline) that maintained during subsequent self-administration behavior. In contrast to temperature increases during the initial self-injections of a session, subsequent regular self-injections did not result in significant temperature changes, although there was a tendency toward temperature decreases within the first 10 min post-injections (Figure 27(d)). These decreases became more evident and significant when the rat received a double-dose heroin injection instead of the regular 0.1 mg/kg injection (Figure 27(e)).

**Figure 28.**
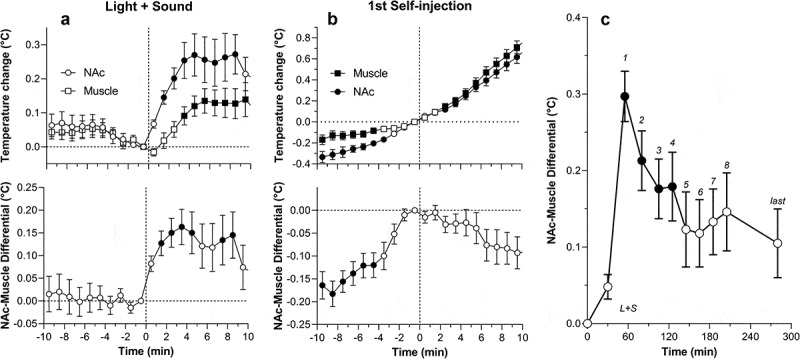
A and B. Mean (±SEM) changes in NAc and muscle temperature and NAc-muscle temperature differentials following exposure to light+sound stimulus that was previously paired with heroin self-injections (conditioned sensory cue A) and preceding the first heroin self-injection of a session (B) in trained rats. Filled symbols show values significantly different from baseline (the last minute before the event). **C. Changes in NAc-muscle temperature differentials during heroin self-administration session in trained rats**. L + S = presentation of a conditioned sensory cue, symbols with numbers = consecutive heroin self-injections. Filled symbols show values significantly different from baseline (0 min). Data were replotted from [229].

Similar as with cocaine self-administration, maximal brain temperature changes during heroin self-administration occurred immediately before the first lever-press of a session. As can be seen in Figure 28(a), the light + sound stimulus that was repeatedly paired with heroin self-injections, induced a significant increase in NAc and muscle temperature, with rapider and stronger changes in the brain vs. the temporal muscle. These changes suggest that this stimulus, due to its repeated pairing with heroin injection, gained a conditioned salience and induces metabolic neural activation. On the other hand, this stimulus triggers drug-seeking behavior that eventually results in the first lever-press. When the data were analyzed retrospectively from the moment of the first lever-press, temperature gradually increased with a significant rise in NAc-muscle differentials immediately prior to this lever-press (Figure 28(b)). Therefore, the gradual brain temperature increase reflects accelerated neural activation that could be viewed as a correlate or driving force of heroin-seeking behavior. Importantly, NAc-muscle differentials did not change more after the first lever-press despite a robust, drug-induced increase in NAc temperature. In fact, the brain-muscle differential slightly but significantly decreased at the moment of the second lever-press and continued to decrease during subsequent drug-taking behavior (Figure 28(c)). While it may seem paradoxical, but neural activation occurring in trained rats self-administering heroin is maximal immediately before the rat makes the first lever-press of a session and receives the first drug injection.

### Pathological hyperthermia induced by MDMA: State and environmental modulation

MDMA (“Ecstasy”, “Molly”) is a typical “club drug” that is usually used by young adults under conditions of physical and emotional activation, often in warm and humid environments [230]. Similar to other psychostimulant drugs, MDMA increases metabolism and induces hyperactivity coupled with hyperthermia [231–235]. MDMA also induces strong peripheral vasoconstriction [231,236], thus diminishing heat dissipation from body surfaces and enhancing heat accumulation in the brain and body.

**Figure 29.**
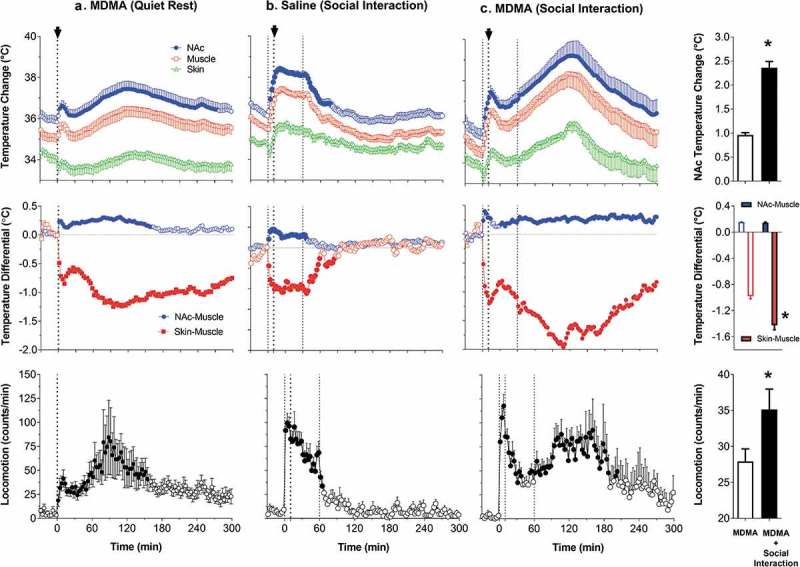
Potentiation of brain and body hyperthermia induced by MDMA (9 mg/kg, sc) during social interaction. When MDMA was injected during quiet rest, it induced a relatively modest increase in NAc and muscle temperature. This response was strongly potentiated when MDMA was injected 10-min after the start of social interaction, which by itself induced strong but transient increases in NAc and muscle temperatures. MDMA in each condition induced increases in NAc-muscle and decreases in skin-muscle differentials. During social interaction, both effects increase, with especially strong and prolonged decrease in skin-muscle differentials, suggesting sustained skin vasoconstriction. Original data were published in [237] (Journal of Neuroscience, open access).

To mimic certain aspects of “party” conditions in rats, we examined temperature effects of MDMA in rats under normal laboratory conditions (quiet rest at 22°C) and compared them to those during neural activation (social interaction with another rat) and in a moderately warm environment (29°C) [237,238]. To mimic the pattern of oral consumption in humans, MDMA was delivered sc at 9 mg/kg. This dose is 2-3-fold larger than the typical consumed dose, but it is only 15–20% of the LD50 [239] and does not induce lethality in rats under standard laboratory conditions (quiet rest at 22–23°C). When tested under these conditions, MDMA induced strong locomotor activation, modestly increased brain and muscle temperature, and rapidly decreased skin temperature (Figure 29(a)). MDMA also induced a strong and prolonged increase in NAc-muscle differentials, suggesting metabolic brain activation, and a strong decrease in skin-muscle differentials, indicating sustained skin vasoconstriction. All of these effects, especially the temperature differential changes, were prolonged and some changes persisted beyond the 5-h recording period. This pattern of temperature response was similar to that induced by social interaction (placement of another male rat in the cage with the recorded male rat for 1 h), but the changes in the latter case were weaker, less prolonged, and clearly dependent on the duration of social interaction (Figure 29(b)).

When MDMA was administered during social interaction, 10 min after another rat was introduced to the cage, increases in brain and muscle temperatures were significantly greater than those in control conditions (~2.4°C vs. ~1°C), with mean values for the NAc exceeding 39°C at their peaks (Figure 29(c)). The overall effect of MDMA during social interaction was the most prolonged, exceeding the entire 5-hr observation window. Despite strong increases in brain temperature that exceeded the upper limits of physiological increases, 6 of 7 rats tolerated this treatment well, but one rat, which showed large temperature elevation, died overnight after the recording session. Social interaction also potentiated metabolic brain activation and skin vasoconstriction, which greatly exceeded physiological changes in these parameters in terms of magnitude and duration.

**Figure 30.**
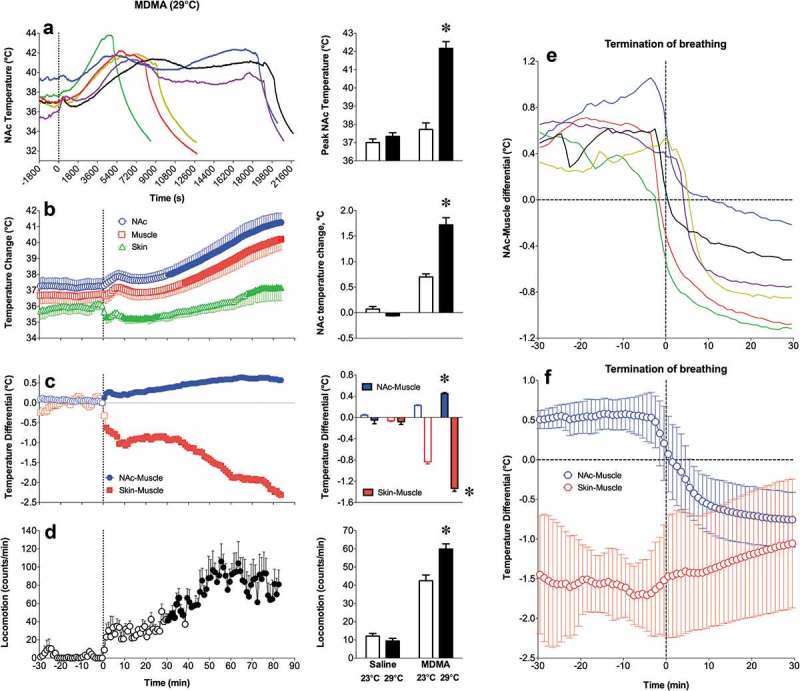
MDMA injected at a dose ~1/5 of the LD50 (9 mg/kg) at normothermic conditions (29°C) induces lethality in all tested rats. A = NAc temperature changes in individual animals; B = changes in NAc, muscle, and skin temperatures for the first 90 min from MDMA injection, when all rats were still alive; C = changes in NAc-muscle and skin-muscle differentials; D = changes in locomotor activity, E = changes in NAc-muscle differentials preceding and following termination of breathing in individual rats; F = mean changes in NAc-muscle and skin-muscle differentials preceding and following termination of breathing. Bar graphs show differences in temperature and locomotor effects for 90 min after MDMA injection at 23°C and 29°C. Original data were published in [237] (Journal of Neuroscience, open access).

Although 29°C is considered a comfortable temperature for rats [240], MDMA administered under these warm conditions induced robust increases in NAc temperatures that reached fatal values (>41°C) and all six tested rats died within 6-h post-injection (Figure 30(a–b)). Peak values of NAc temperatures after MDMA administration ranged from 41.4°C to 43.8°C, and their means were more than 4°C larger than in the control condition. When calculated with respect to the pre-lethality temperature peak, the mean increase in NAc temperature was ~2.3°C, significantly larger than the maximum temperature elevation induced by MDMA under quiet resting conditions at 23°C (~1.0°C). MDMA administered in warm temperatures also induced more prolonged and stronger increases in NAc-muscle differentials, suggesting intense heat production, and much stronger and more prolonged decreases in skin-muscle differentials, indicating sustained skin vasoconstriction (Figure 30(c)).

Along with the robust, accelerated rise in brain and muscle temperature preceding termination of breathing, brain-muscle differentials also increased abnormally, but then rapidly decreased within 2–4 min around the moment of death (Figure 30(e–f)). This change is a critical feature of brain death, which occurs within several minutes after full termination of breathing. This change, when the dying brain becomes colder than the still alive body, appears to be typical to humans. Decreases in brain temperature following brain death far exceeded that of esophageal and rectal temperatures and the brain at this moment becomes the coldest location in the body [241].

Powerful enhancement of the hyperthermic effects of MDMA found in rats during social interaction and in warm environments may help to explain the exceptionally strong, sometimes fatal, responses of humans to this drug used at moderate doses under “rave party” conditions [230,242]. Therefore, pathological brain hyperthermia induced by overdose of psychomotor stimulants under rave conditions results not only from excessive heat production due to drug-induced and associated psycho-physiological activation, but also from strong and sustained peripheral vasoconstriction that prevents proper heat dissipation to the external environment. Since partygoers are often engaged in intense physical interactions with other individuals (dancing, sexual activity, etc.), the effects of MDMA are additionally potentiated by the psycho-physiological activation typical to these conditions. Similar potentiation of temperature effects by social interaction and warm ambient temperatures was also seen with METH, another psychostimulant drug, which is often taken under conditions of rave parties [150,243].

## Conclusions and functional implications

The brain is simultaneously a part of the body and the center for regulating physiological functions. However, the brain has some degree of autonomy from the rest of the body and differs from it in terms of regulating its basic parameters (i.e. metabolism, cerebral blood flow, temperature, water and ion content). Although the concepts of regulation and homeostasis are basic ideas of physiology, it is unclear whether this logic of regulation can be applied to the temperature of the brain itself and whether brain temperature can be considered a true homeostatic parameter. Data presented in this review demonstrate that brain temperature fluctuates profoundly (3–4°C) within a normal physiological and behavioral continuum. While hypothalamic temperature directly measured in rats during day-time sleep in a familiar environment can transiently fall to 35.4–35.6°C [113] it transiently peaks at 39.6–39.8°C during male copulatory behavior at the time of ejaculation [59,92]. Therefore, brain temperature fluctuations within this range can be viewed as a part of normal brain functioning and not an index of pathology. Although most of the data presented in this review were obtained in rats and direct human data are limited, a general similarity in brain temperature responses in rats and monkeys [30], as well as robust hyperthermia induced in humans during intoxication with psychomotor stimulants and during intense physical exercise in adverse environmental conditions, suggest the applicability of basic data obtained in rats to human conditions. Although humans have more efficient mechanisms of heat dissipation than rats that allowed them to lose more heat than can be maximally produced, under specific real-life conditions these mechanisms fail, resulting in pathological hyperthermia. Brain temperature can also be shifted above or below its physiological range by different neuroactive drugs that affect metabolism and the tone of central and peripheral blood vessels.

While in quietly resting rats basal temperatures in different structures follow the dorso-ventral gradient and in deep structures they are close to or slightly higher than in the body core, brain temperature rapidly increases following exposure to various environmental stimuli and these increases are faster and stronger than those in the arterial blood and peripheral bodily locations. Brain temperature also tonically increases during different motivated behaviors, showing rapid biphasic fluctuations associated with critical behavioral events. Since arterial blood arriving to the brain is always cooler than brain tissue, metabolic brain activation with subsequent intra-brain heat production appears to be a critical factor determining physiological brain temperature increases. As shown through multiple examples, this contributor to brain temperature response can be assessed by calculating temperature differentials between a brain site and arterial blood or a temporal muscle as its proxy. These parameters transiently increased following exposure to all environmental stimuli and tonically decreased during general anesthesia. Neural activation elicited by arousing stimuli is also accompanied by sympathetic activation, affecting bodily heat production, the tone of cerebral and peripheral blood vessels, and heat exchange between brain tissue and arterial blood. As assessed through changes in skin-muscle differentials, arousing stimuli induce peripheral vasoconstriction, thus diminishing heat loss to the external environment and contributing to increases in brain temperature. Brain temperature is also affected by changes in cerebral blood flow, which is enhanced during neural activation via a neurovascular mechanism [244] and due to peripheral vasoconstriction that re-distribute blood from the periphery to the brain [245]. Therefore, increased cerebral blood flow not only provides oxygen and nutrients for enhanced brain metabolism but also removes potentially dangerous metabolic heat from neural tissue, thus affecting the patterns of brain temperature fluctuations. While intra-brain heat production is an obvious by-product of cerebral metabolism, physiological fluctuations in brain temperature affect various neural parameters ranging from the activity of single ionic channels to transmitter release and uptake. Thus, physiological brain hyperthermia has adaptive significance, altering the dynamics of neural functions and making them more efficient for achieving behavioral goals.

Due to the physical nature of temperature and continuous heat exchange within the entire brain, temperature fluctuations occurring in different brain structures share basic similarity when analyzed with slow time-resolution. Slow time-resolution analysis also minimizes differences in temperature responses between individual brain structures and the body core, thus making these changes correlative. However, high-resolution data analysis reveals between-structure differences in temperature dynamics, but these differences are minor and quantitative in nature. While it can be hypothesized that distinct temperature changes detected in each brain structure reflect differences in neuronal activity state, the major component of these changes appears to be nonspecific, involving the brain as a whole. However, this does not mean that these distinct changes are unimportant, or that they should be ignored. To some extent, physiological brain temperature fluctuations are similar to changes in integral electrical activity evaluated by EEG recordings. The changes in EEG signal (i.e. total power, distribution of frequency waves) induced by various sensory stimuli in animals and humans show basic similarity in different brain structures and only sophisticated analyses reveal minor differences depending on the stimulus nature and the area of recording.

As discussed in this review, physiological brain temperature fluctuations can be considered from two standpoints. From one standpoint, they reflect changes in neural activity and general activational processes, but from another standpoint they affect neural activity, neural functions, and multiple electrophysiological and neurochemical evaluations conducted in awake, freely moving animals. While our initial goal was to learn how brain temperature fluctuates under different experimental conditions and clarify the mechanisms underlying these fluctuations, the findings obtained in these studies allowed us to learn more about basic neural mechanisms involved in organization and regulation of goal-directed behavior.

Most current studies aimed at understanding the organization and regulation of motivated behaviors focus on specific changes in neuronal activity in different brain structures as the components of neural circuits underlying these behaviors. However, some basic changes in neuronal activity occurring in awake, freely moving animals share general similarity and they appear to be common for different brain structures. This commonality of changes occurring on different levels of the CNS can be viewed in terms of an arousal as a nonspecific neural activation common to quite different physiological and behavioral responses to various environmental challenges. This arousal-related neural activation is part of a general arousal response that also involves the autonomic nervous system, endocrine system, and multiple physiological functions. The concept of stress, as a nonspecific adaptive response to any activating (“stress-inducing” or potentially dangerous) stimulus, is another way to explain a similar pattern of neural and physiological responses to quite different stimuli. While the arousal concept is focused on generalized neural activation as an electrophysiological event, the concept of stress is focused on common changes in autonomic and hormonal responses induced by stimuli of different nature (sensory, chemical, psychological, etc.). As shown in this review, this common pattern was typical for brain temperature responses elicited by quite different arousing stimuli. However, these responses varied in magnitude and duration, obviously reflecting the salience or arousing potential of each stimulus. In each case, however, the brain temperature response was dependent on basal temperature, reflecting the basic relationship between the response to an activating stimulus and basal activity state (Wilder’s law of basal values).

In contrast to monophasic and transient brain temperature increases elicited by arousing stimuli, more complex multi-phasic temperature fluctuations occurred during several types of natural motivated behaviors and drug self-administration. Despite differences in nature of the reinforcer (food, sugary drink, opposite-sex partner, neuroactive drug), the means for obtaining it (free access, searching activity, lever-press that triggers drug injection) and associated locomotor activity, the observed brain temperature fluctuations shared basic similarities, which could reflect common changes in neural activity, brain activational processes and physiological parameters associated with any type of goal-directed behavior.

As shown in this review, drugs of abuse, through affecting metabolism and vascular tone, induce distinct changes in brain and body temperature that depend on the nature of the drug, its dose, an organism’s activity state and the environmental conditions associated with its administration. While the knowledge of temperature effects of drugs of abuse is important for understanding how they affect neural activity and physiological functions, our studies of cocaine and heroin self-administration allowed us to reveal some common features in both behaviors and their general similarity with natural goal-directed behaviors. Drug-induced temperature changes also affect multiple neural functions and could be the cause of or a critical contributor to serious health complications during drug overdose. This issue can be especially important for psychostimulant drugs of abuse such as MDMA and METH that are often taken at high doses, during high activity states and in hot and humid environments that prevent proper heat dissipation to the external environment. While the hyperthermic effects of heroin become inverted with increases in dose and the effects of cocaine become progressively weaker at higher basal temperatures, both MDMA and METH induce dose-dependent brain hyperthermia, which is strongly potentiated during physiological activation and under specific environmental conditions that restrict heat dissipation. Because high temperatures exacerbate drug-induced toxicity and are destructive to neural cells and brain functions, pathological brain hyperthermia induced by METH and MDMA overdose is an important contributor to both acute life-threatening complications and chronic destructive CNS changes induced by these drugs.

Pathological hyperthermia can also result from impaired heat dissipation during intense physical activity in a hot, humid environment. While under these conditions temperature in arterial blood remains cooler than in brain tissue [86], this natural cooling mechanism becomes less and less effective and finally fails because of progressive body temperature increases (and subsequent arterial temperature increases) due to an inability to dissipate metabolic heat to the external environment. In this case, brain hyperthermia is a critical part of a general over-heating (heat shock) syndrome, which negatively affects neural functions and brain structure and can potentially result in lethality. This issue could be especially important for specific groups of people, including Marathon runners, football players, and military personnel, who are engaged in intense physical activity in hot and warm environments and have body protection that prevents heat dissipation to the external environment.

Brain thermorecording was introduced in the early years of experimental physiology as a tool to assess neural activity and psychic functions. Although many interesting studies were conducted since the initial experiments of Broca, Lombard and Schiff in the XIX century, brain thermorecording is still rarely used and brain temperature remains out of interest for most physiologists and neuroscientists. The goal of this review was to shed light on brain temperature as an important physiological parameter and to consider the mechanisms and functional significance of physiological brain temperature fluctuations and their effects on neurophysiological and neurochemical evaluations conducted in awake, behaving organisms. I also stressed the factors and conditions that could induce pathological shifts in brain temperature, with dramatic alterations of brain homeostatic parameters, damaging effects on neural cells and decompensation of an organism’s physiological functions. While the brain regulates the activity of the whole organism, maintaining the stability of body temperature, it is also a part of the body and its temperature also depends on body temperature and heat exchange between the organism and its physical environment. Being a part of an organism’s natural functioning, brain temperature fluctuations have global effects on neural activity and neural functions. My hope is that brain temperature will be better accounted for and studied further in the pursuit of understanding its intricate role in every aspect of physiology and neuroscience.

## Abbreviations

BBB: blood-brain barrier
CNS: central nervous system
DA: dopamine
DOPAC: 3,4-Dihydroxyphenylacetic acid
EEG: electroencephalogram
GFAP: glial fibrillary acidic protein
GLU: glutamate
Hippo: hippocampus
ip: intraperitoneal
iv: intravenous
METH: methamphetamine
MDMA: 3,4-methylenedioxymethamphetamine
mPOA: medial preoptic area of hypothalamus
NAc: nucleus accumbens
sc: subcutaneous
VTA: ventral tegmental area of midbrain
